# Targeting melanoma’s MCL1 bias unleashes the apoptotic potential of BRAF and ERK1/2 pathway inhibitors

**DOI:** 10.1038/s41467-019-12409-w

**Published:** 2019-11-14

**Authors:** Matthew J. Sale, Emma Minihane, Noel R. Monks, Rebecca Gilley, Frances M. Richards, Kevin P. Schifferli, Courtney L. Andersen, Emma J. Davies, Mario Aladren Vicente, Eiko Ozono, Aleksandra Markovets, Jonathan R. Dry, Lisa Drew, Vikki Flemington, Theresa Proia, Duncan I. Jodrell, Paul D. Smith, Simon J. Cook

**Affiliations:** 1 Signalling Programme, The Babraham Institute, Babraham Research Campus, Cambridge, CB22 3AT UK; 2 Oncology R&D, AstraZeneca, One Medimmune Way, Gaithersburg, MD 20878 USA; 3 Pharmacology and Drug Development Group, Cancer Research UK Cambridge Institute, University of Cambridge, Li Ka Shing Centre, Robinson Way, Cambridge, CB2 0RE UK; 4 Oncology R&D, AstraZeneca, 35 Gatehouse Drive, Waltham, MA 02451 USA; 5 Oncology R&D, AstraZeneca, Cancer Research UK Cambridge Institute, University of Cambridge, Li Ka Shing Centre, Robinson Way, Cambridge, CB2 0RE UK; 6 CRUK Therapeutic Discovery Laboratories, Jonas Webb Building, Babraham Research Campus, Cambridge, CB22 3AT UK

**Keywords:** Targeted therapies, Cell death, Cell signalling

## Abstract

BRAF and MEK1/2 inhibitors are effective in melanoma but resistance inevitably develops. Despite increasing the abundance of pro-apoptotic BIM and BMF, ERK1/2 pathway inhibition is predominantly cytostatic, reflecting residual pro-survival BCL2 family activity. Here, we show that uniquely low BCL-X_L_ expression in melanoma biases the pro-survival pool towards MCL1. Consequently, BRAF or MEK1/2 inhibitors are synthetic lethal with the MCL1 inhibitor AZD5991, driving profound tumour cell death that requires BAK/BAX, BIM and BMF, and inhibiting tumour growth in vivo. Combination of ERK1/2 pathway inhibitors with BCL2/BCL-w/BCL-X_L_ inhibitors is stronger in CRC, correlating with a low MCL1:BCL-X_L_ ratio; indeed the MCL1:BCL-X_L_ ratio is predictive of ERK1/2 pathway inhibitor synergy with MCL1 or BCL2/BCL-w/BCL-X_L_ inhibitors. Finally, AZD5991 delays acquired BRAFi/MEKi resistance and enhances the efficacy of an ERK1/2 inhibitor in a model of acquired BRAFi + MEKi resistance. Thus combining ERK1/2 pathway inhibitors with MCL1 antagonists in melanoma could improve therapeutic index and patient outcomes.

## Abstract (web summary)

BRAF or MEK1/2 inhibitors are cytostatic in melanoma and the surviving cells develop drug resistance. This study shows that the pro-survival pool is biased towards MCL1 in melanoma so that BRAF or MEK1/2 inhibitors are synthetic lethal with the MCL1 inhibitor AZD5991, improving tumour growth inhibition.

## Introduction

BRAF inhibitors (BRAFi, vemurafenib and dabrafenib) and MEK1/2 inhibitors (MEKi, trametinib) improve the outcomes of patients with BRAF^V600E/K^-mutant melanoma^1–3^. However, disease relapse typically occurs due to acquired resistance that reinstates ERK1/2 signalling^4,5^. Although combining BRAFi and MEKi improves responses^6,7^, acquired resistance and disease relapse are only delayed and again typically arise through reactivation of ERK1/2^4,5^. Thus, strategies are required that boost the primary efficacy of BRAFi and MEKi to delay or prevent the emergence of resistance.

The cell intrinsic apoptotic pathway is regulated by BCL2 family proteins, which are frequently deregulated in cancer, typically through aberrant expression, activity or deregulation of oncogenic signalling cascades. ERK1/2 can promote cell survival in BRAF- or KRAS-mutant tumour cells^8^ by increasing expression of pro-survival BCL2 proteins. ERK1/2 stabilise MCL1 through phosphorylation of T163 and indirectly through RSK-mediated inhibition of GSK3^9^. ERK1/2 signalling also drives transcription of *MCL1*, *BCL2* and *BCL-X*_*L*_^8^. In addition, ERK1/2 signalling inhibits the pro-apoptotic BH3-only proteins BIM, BMF, PUMA and BAD. ERK1/2 directly phosphorylate BIM, targeting it for ubiquitination and proteasomal degradation^10^. ERK1/2 signalling also represses BMF protein expression^11^, and promotes BAD inactivation through MSK/RSK-catalysed phosphorylation and sequestration by 14-3-3 proteins^8^. Although ERK1/2 have been proposed to regulate BIK in a manner analogous to BIM, this is disputed^12,13^. ERK1/2 signalling also represses *BIM* and *PUMA* transcription by destabilising FOXO3A^14–17^. As a result, inhibition of ERK1/2 signalling in tumour cells invariably promotes the expression of pro-apoptotic BIM, BMF and/or PUMA^11^. Despite this, apoptotic responses to ERK1/2 pathway inhibitors are typically weak because of residual activity of pro-survival BCL2 proteins.

Among the agents developed to inhibit pro-survival proteins and drive tumour cell apoptosis^18^, drugs that mimic the BH3 domains of BH3-only proteins (BH3-mimetics) are the most advanced. Venetoclax (ABT-199), a BCL2-selective inhibitor, has been approved for clinical use. Navitoclax (ABT-263) and AZD4320 target BCL2, BCL-w and BCL-X_L_ but not MCL1 or A1^19–22^. AZD4320 has nanomolar affinity for BCL2 and BCL-X_L_ and physicochemical properties suitable for intravenous administration, which may avoid toxicities observed with oral administration of navitoclax^21,23,24^. BCL2/BCL-w/BCL-X_L_ inhibitors are showing promise in haematological malignancies such as chronic lymphocytic leukaemia (CLL), but will require combination to be effective in solid tumours. Indeed, ERK1/2 pathway inhibitors combine with navitoclax, or the close analogue ABT-737, to induce colorectal cancer (CRC) apoptosis and tumour regression in vivo^11,25,26^. This combination may also be effective in non-small cell lung cancer (NSCLC) and pancreatic tumours; however, we and others have noted more limited synergy between ERK1/2 pathway inhibitors and navitoclax/ABT-737 in melanoma^11,26–28^. BCL-X_L_ and MCL1 are the major pro-survival proteins in solid tumours (Cancer Cell Line Encyclopaedia (CCLE; https://portals.broadinstitute.org/ccle) and The Cancer Genome Atlas (TCGA; https://cancergenome.nih.gov/)), but development of MCL1 inhibitors (MCL1i) has lagged behind that of BCL2/BCL-w/BCL-X_L_ inhibitors due to challenges associated with targeting the MCL1 BH3-binding groove^8,29,30^. However, since potent and selective MCL1i are now in clinical development, including S63845^31^, AMG 176^32^ and AZD5991^33^, it is imperative to identify drug combinations and disease stratification criteria to maximise their impact.

Here, we show that the pro-survival BCL2 family pool is biased towards MCL1 in melanoma compared to CRC, NSCLC and pancreatic tumour lineages, due to low BCL-X_L_ expression. Thus, MCL1 is critical in restraining pro-apoptotic BH3-only proteins induced by ERK1/2 inhibition in melanoma. Consequently, combined inhibition of ERK1/2 signalling and MCL1 is synthetic lethal, inducing profound, synergistic BAK/BAX-, BIM- and BMF-dependent apoptosis and tumour regression. Finally, combining ERKi and MCL1i overcomes acquired resistance to combined BRAFi + MEKi. Thus, exploiting specific inhibition of ERK1/2 signalling and apoptotic priming in BRAF-mutant cells coupled with the pro-survival bias towards MCL1 could afford a large therapeutic window and further improve patient outcomes in melanoma.

## Results

### The melanoma pro-survival BCL2 family pool is MCL1 biased

**Fig. 1:**
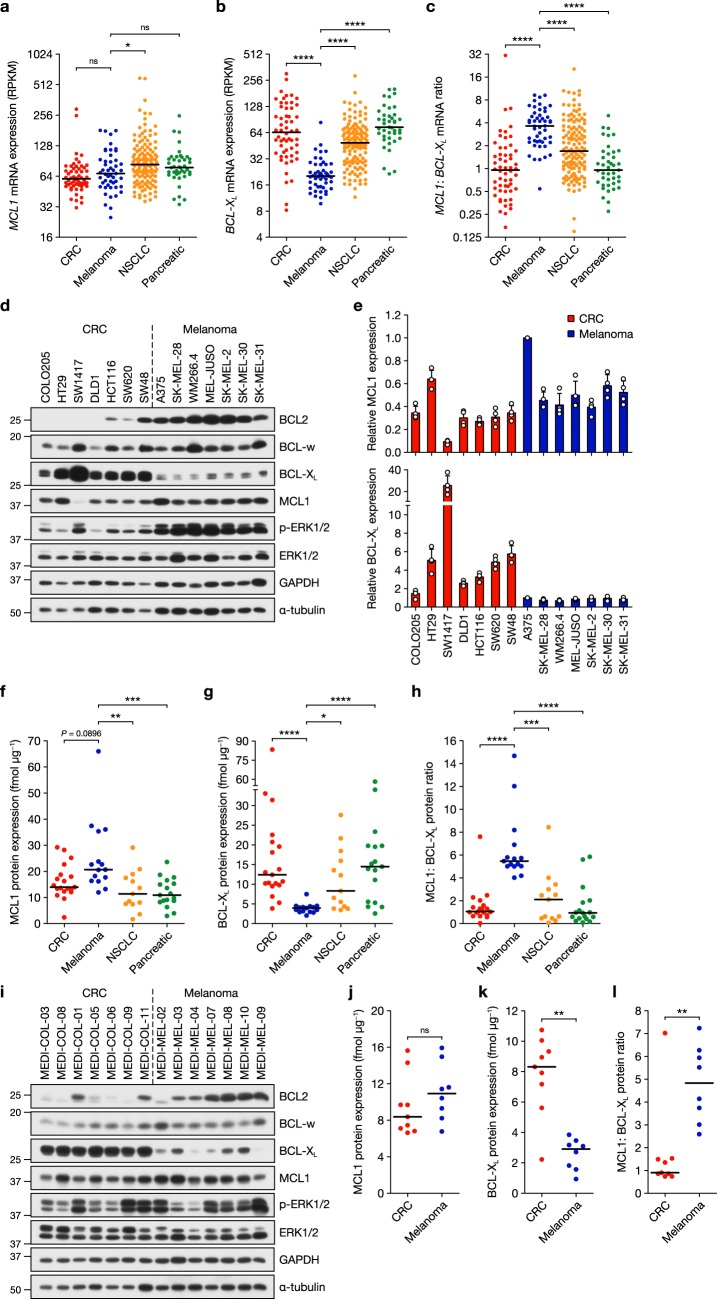
Low BCL-X_L_ expression in melanoma biases the pro-survival pool towards MCL1. **a**–**c** RNA-sequencing mRNA expression data obtained from the Cancer Cell Line Encyclopaedia for *MCL1* (**a**) *BCL-X*_*L*_ (*BCL2L1*) (**b**) and *MCL1*:*BCL-X*_*L*_ ratio (**c**) in colorectal cancer (CRC), melanoma, non-small cell lung cancer (NSCLC) and pancreatic tumour cells. Expression values are reads per kilobase million (RPKM) and are presented on a log_2_ scale. Black lines indicate median values. **d**, **e** Seven CRC (left) and seven melanoma (right) cell lines were cultured for 24 h before lysis and western blotting with the indicated antibodies. Quantification (**e**) of MCL1 and BCL-X_L_ levels relative to A375 cells was performed by quantitative western blotting with fluorescently labelled secondary antibodies. **f**, **g** MCL1 (**f**) and BCL-X_L_ (**g**) expression were quantified absolutely in CRC, melanoma, NSCLC and pancreatic tumour cell lines using recombinant protein standards and quantitative western blotting with fluorescently labelled secondary antibodies. Each point represents mean expression in a cell line from three independent experiments and is expressed in fmol per µg of total cellular protein (fmol  µg^−1^). Black lines indicate median values. **h** Ratio of MCL1 to BCL-X_L_ protein calculated from **f** and **g**, with black lines indicating median values. **i** Seven CRC (left) and seven melanoma (right) patient-derived xenograft (PDX) tumours were homogenised in lysis buffer and western blotted with the indicated antibodies. **j**, **k** Absolute quantification of MCL1 (**j**) and BCL-X_L_ (**k**) expression in CRC and melanoma PDX tumour samples using recombinant protein standards and quantitative western blotting with fluorescently labelled secondary antibodies. Each point represents mean expression in a cell line from at least five technical replicates and is expressed in fmol per µg of total cellular protein (fmol µg^−1^). Black lines indicate median values. **l** Ratio of MCL1 to BCL-X_L_ protein calculated from (**j**) and (**k**), with black lines indicating median values. Results (**e**–**h**, **j**–**l**) are the mean of three or more independent experiments, and error bars indicate SD. *P* ≤ 0.0001 (****), *P* ≤ 0.001 (***), *P* ≤ 0.01 (**), *P* ≤ 0.05 (*), ns (not significant) as determined by Kruskal–Wallis and Dunn’s multiple comparisons tests (**a**–**c**, **f**–**h**) or two-tailed Mann–Whitney test (**j**–**l**)

We first examined RNA-sequencing (RNA-seq) data available in the CCLE for transcripts encoding pro-survival BCL2 proteins^34^. While *MCL1* expression in the CCLE data set was broadly similar in CRC and melanoma cells, and slightly higher in NSCLC and pancreatic, levels of *BCL2L1* (encoding BCL-X_L_) were strikingly lower in melanoma relative to the other lineages (Fig. 1a, b). Consequently, the *MCL1*:*BCL-X*_*L*_ mRNA ratio, encoding the major pro-survival proteins in solid tumours, was two- to four-fold higher in melanoma than in the other lineages (Fig. 1c). Indeed, of all the tumour lineages in the CCLE, melanoma exhibited one of the highest median *MCL1*:*BCL-X*_*L*_ mRNA ratios (Supplementary Fig. 1a). Notably autonomic ganglia tumour cells which, like melanocytes, have neural crest developmental origin also exhibited a high *MCL1*:*BCL-X*_*L*_ ratio. While *BCL2* expression was higher in melanoma, *BCL2* mRNA levels (RNA-seq read number) were very low in each lineage relative to *MCL1* and *BCL-X*_*L*_ (Supplementary Fig. 1b). *BCL2L2* (encoding BCL-w) levels were similar in each lineage (Supplementary Fig. 1c) and *BCL2A1* (encoding A1/BFL1), a MITF target gene^35^, exhibited melanoma-selective expression (Supplementary Fig. 1d). Similar observations have been made in TCGA patient samples (https://cancergenome.nih.gov/). We confirmed these trends at the protein level in seven melanoma and seven CRC cell lines (Fig. 1d, e). All melanoma cell lines exhibited strikingly lower BCL-X_L_ expression than the CRC cell lines whereas MCL1 was, on the whole, marginally higher in melanoma (Fig. 1d, e). BCL2 expression was markedly elevated in melanoma (Fig. 1d and Supplementary Fig. 2a), consistent with *BCL2* being a MITF target gene^36^. BCL-w levels were similar between CRC and melanoma (Fig. 1d and Supplementary Fig. 2b). We failed to detect A1 protein expression using a variety of commercially available antibodies, even in melanoma cells with high *BCL2A1* mRNA.

Next, we used recombinant protein standards (Supplementary Fig. 2c, d) to absolutely quantify MCL1 and BCL-X_L_ in 64 cell lines (Supplementary Fig. 2e–h), and thereby determine the MCL1:BCL-X_L_ protein ratio (Fig. 1f–h). MCL1 levels were similar across the four lineages but higher in melanoma (~20 vs. <14 fmol per μg total cellular protein; Fig. 1f and Supplementary Fig. 2f). Consistent with mRNA levels, BCL-X_L_ protein was strikingly lower in melanoma (Fig. 1g and Supplementary Fig. 2f) compared to CRC, NSCLC and pancreatic lineages (median value of <4 fmol vs. >11 fmol per μg total cellular protein). Consequently the MCL1:BCL-X_L_ ratio was substantially biased towards MCL1 in melanoma (Fig. 1h); the median ratio was 5–6 in melanoma vs. ~1 in CRC and pancreatic tumour cells, and ~2 in NSCLC (Fig. 1h). The same analysis in CRC and melanoma patient-derived xenograft (PDX) samples (Fig. 1i–l) demonstrated markedly low BCL-X_L_ expression in melanoma PDXs vs. CRC (Fig. 1i, k), whereas MCL1 expression was broadly similar (Fig. 1i, j). Consequently, the MCL1:BCL-X_L_ protein ratio was ~5 times higher in melanoma (Fig. 1l). Thus, the ratio of the two major pro-survival proteins in solid tumours, MCL1 and BCL-X_L_, is substantially biased towards MCL1 in melanoma cell lines and patient samples.

### BRAFi or MEKi combine with MCL1i to selectively kill melanoma cells

**Fig. 2:**
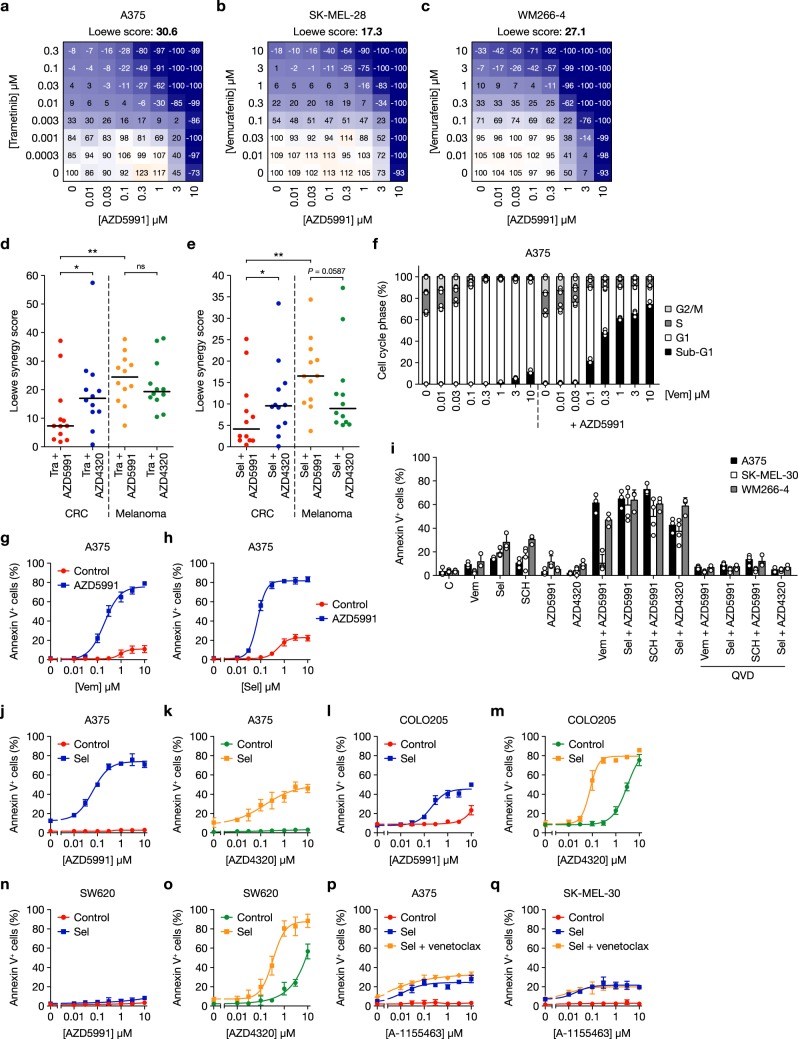
ERK1/2 pathway inhibitors combine with the MCL1 inhibitor AZD5991 to kill melanoma cells. **a**–**c** A375 (**a**), SK-MEL-28 (**b**) and WM266-4 (**c**) cells were treated with the indicated concentrations of trametinib or vemurafenib in combination with AZD5991 for 5 days. The number of viable cells was determined at the point of treatment (day 0) and at the end of the experiment using Sytox Green. One-hundred percent represents the number of viable cells in the control wells at day 5, 0% is equivalent to the number of viable cells on day 0 and −100% indicates that no viable cells remained on day 5. **d**, **e** Twelve melanoma and 12 colorectal cancer (CRC) cell lines were treated with trametinib (Tra) or selumetinib (Sel) and AZD5991 or AZD4320 in an 8 × 8 concentration matrix for 5 days. Viable cell number (determined as above) was used to calculate Loewe synergy scores using the Loewe additivity model. *P* ≤ 0.01 (**), *P* ≤ 0.05 (*), ns (not significant) as determined by unpaired or paired two-tailed *t*-test. **f** A375 cells were treated with increasing concentrations of vemurafenib (Vem) with or without 1 μM AZD5991 for 48 h. Cell cycle profile was determined by propidium iodide staining and flow cytometry. **g**, **h** A375 cells were treated with increasing concentrations of vemurafenib (Vem) (**g**) or selumetinib (Sel) (**h**) with or without 1 μM AZD5991 for 48 h. **i** The indicated tumour cell lines were treated with DMSO control (C), 1 μM vemurafenib (Vem), 1 μM selumetinib (Sel) and 0.3 μM SCH772984 (SCH) alone or in combination with 1 μM AZD5991, 1 μM AZD4320 and 20 μM Q-VD-OPh (QVD) for 48 h. **j**–**o** A375 (**j**, **k**), COLO205 (**l**, **m**) or SW620 (**n**, **o**) cells were treated with increasing concentrations of AZD5991 (**j**, **l**, **n**) or AZD4320 (**k**, **m**, **o**) with or without 1 μM selumetinib (Sel) for 48 h. **p**, **q** A375 (**p**) and SK-MEL-30 (**q**) cells were treated with increasing concentrations of A-1155463 with or without 1 μM selumetinib (Sel) and 3 μM venetoclax for 48 h. **g**–**q** Apoptosis was assessed by Annexin V positivity using flow cytometry. Results (**a**–**q**) are the mean of at least three independent experiments and error bars show SD

Combining BRAFi or MEKi with pan-BCL2/BCL-w/BCL-X_L_ inhibitors causes strong synergistic cell death of CRC, NSCLC and pancreatic cells and tumour regressions^11,25,26^. However, we and others observed more modest synergy in melanoma^11,26–28^. This difference could be explained by the bias towards MCL1 in melanoma (Fig. 1 and Supplementary Figs. 1 and 2). We therefore tested ERK1/2 pathway inhibitors in combination with the novel MCL1i AZD5991^33^ using high-throughput combination assays (HTCA) in an 8 × 8 concentration matrix and determining Loewe synergy scores in 12 melanoma and CRC cell lines. These responses were compared to those with the BCL2/BCL-w/BCL-X_L_ inhibitor AZD4320, which has nanomolar affinity for these pro-survival proteins, similar to navitoclax^21,23,24^. Combination of MEKis trametinib or selumetinib (AZD6244, ARRY-142886) with AZD5991 or AZD4320 was performed in all CRC and melanoma cell lines to allow comparison of BRAF^MUT^, RAS^MUT^ or RAS/RAF^WT^ cells (Supplementary Table 1); combinations with the BRAFi vemurafenib (only effective in BRAF^V600^-mutant cells) or ERK1/2 inhibitor SCH772984 were tested in selected cell lines. Data from these combination assays are shown in Fig. 2a–c and Supplementary Figs. 3–6 where trametinib, selumetinib or vemurafenib combined with AZD5991 to cause strong cell death in BRAF^V600E^-mutant A375, SK-MEL-28, WM266-4, A2058 or SK-MEL-5 melanoma cells. MEKis or ERKi, also combined well with AZD5991 in NRAS-mutant MEL-JUSO, SK-MEL-2, SK-MEL-30 and WM852 melanoma cells (Supplementary Figs. 3–6). In these melanoma cells, ERK1/2 pathway inhibitors typically combined less well with AZD4320 than AZD5991, whereas trametinib or selumetinib combined better with AZD4320 than AZD5991 in HCT116 and HT29 CRC cells (Supplementary Fig. 5). Loewe synergy score calculation for all 24 cell lines (Supplementary Table 2) revealed that both AZD5991 and AZD4320 synergised (score ≥ 5) with trametinib and selumetinib in the majority of cell lines (Fig. 2d, e). However, synergy with AZD5991 was typically far stronger in melanoma: median synergy scores for trametinib or selumetinib combined with AZD5991 were >3 times higher in melanoma than CRC, whereas combination with AZD4320 was more effective than with AZD5991 in CRC (>2-fold higher median synergy score).

We confirmed these high-throughput results in selected cell lines by examining cell cycle profiles. Vemurafenib (Fig. 2f) or selumetinib (Supplementary Fig. 7a) resulted in a strong G1 arrest in BRAF-mutant A375 cells but only limited cell death (<10% sub-G1 DNA fraction) at higher concentrations. 1 μM AZD5991 had no effect on cell cycle or cell death; however, combination of AZD5991 with 1 μM vemurafenib or selumetinib elicited 65–75% cell death (Fig. 2f and Supplementary Fig. 7a). In A375 cells, 10 µM AZD5991 had no effect on cell cycle profile, whereas concentrations as low as 100 nM combined with selumetinib to induce substantial synergistic cell death (Supplementary Fig. 7b). Very similar results were observed in NRAS^Q61K^-mutant SK-MEL-30 melanoma cells (Supplementary Fig. 7c, d). Further experiments focused on annexin V-positive, apoptotic cells. In A375 cells both vemurafenib and selumetinib combined synergistically with AZD5991 to drive ~80% apoptosis following 48 h treatment (Fig. 2g, h); similar results were observed with vemurafenib in BRAF-mutant WM266-4 (Supplementary Fig. 7e) and selumetinib in NRAS-mutant SK-MEL-30 cells (Supplementary Fig. 7f). BRAFi (vemurafenib), MEKi (selumetinib) or ERKi (SCH772984) combined to kill BRAF-mutant A375 and WM266-4 cells in a caspase-dependent fashion (Fig. 2i) but only selumetinib or SCH772984 were effective in NRAS^Q61K^-mutant SK-MEL-30 cells, consistent with vemurafenib efficacy being confined to BRAF-mutant tumour cells. In contrast, and consistent with results from the HTCA experiments, A375 (Fig. 2j, k) and SK-MEL-30 cells (Supplementary Fig. 7g, h) both exhibited markedly weaker apoptotic responses when selumetinib was combined with the BCL2/BCL-w/BCL-X_L_ inhibitor AZD4320 rather than AZD5991.

The inverse was true for the CRC cell lines COLO205 and SW620. While selumetinib and AZD5991 combined to induce apoptosis in COLO205 cells (Emax ~ 45%), far stronger synergistic apoptosis was apparent with AZD4320 (Emax ~ 80%) (Fig. 2l, m). SW620 cells were an even more striking example: combination of selumetinib with 10 µM AZD5991 resulted in <10% apoptosis, whereas ~90% apoptosis was achieved when selumetinib was combined with AZD4320 (Fig. 2n, o). Indeed, in COLO205 and SW620, concentrations of AZD4320 >1 µM elicited considerable apoptosis alone (Fig. 2m, o), suggesting a stronger dependency upon BCL2, BCL-w and/or BCL-X_L_ for basal viability in CRC. Combination of navitoclax with selumetinib promoted only modest apoptosis at near-identical potency and maximal response as AZD4320 in A375 and SK-MEL-30 (Supplementary Fig. 7i, j), consistent with AZD4320 and navitoclax having similar pharmacology^21,23,24^.

To investigate the role of the individual pro-survival proteins inhibited by AZD4320/navitoclax in melanoma, we used selective inhibitors of BCL2 (venetoclax, ABT-199) and BCL-X_L_ (A-1155463)^37^. Selumetinib combined weakly with A-1155463 in A375 or SK-MEL-30 (Fig. 2p, q) to induce a similar apoptotic response as with AZD4320 or navitoclax (Fig. 2k and Supplementary Fig. 7h–j). Despite higher levels of BCL2 in melanoma relative to CRC (Fig. 1d and Supplementary Fig. 1b) venetoclax monotherapy had no effect on viability and did not combine with selumetinib to induce apoptosis, although venetoclax marginally enhanced the combination effect of A-1155463 and selumetinib in A375 cells (Fig. 2p and Supplementary Fig. 7k, l). Thus, melanoma cells were not addicted to BCL2 for viability when the ERK1/2 pathway was inhibited. BCL2 inhibition may, however, slightly enhance the effect of BCL-X_L_ inhibition when the ERK1/2 pathway is inhibited in some melanoma cells. These results indicate that different tumour lineages exhibit selective dependencies upon distinct BCL2 proteins for survival, with melanoma exhibiting a strong bias towards MCL1 and CRC an apparent bias towards BCL-X_L_.

**Fig. 3:**
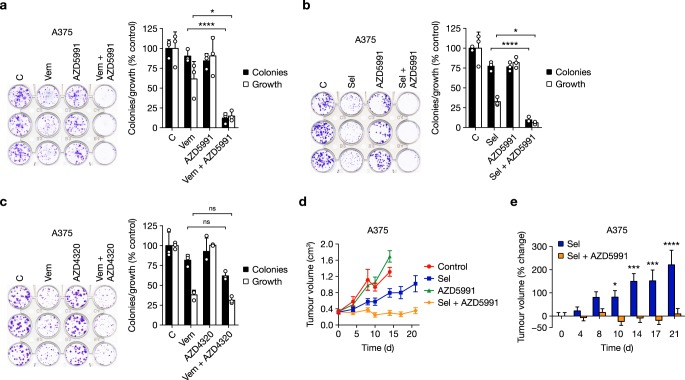
BRAFi or MEKi combine with AZD5991 to inhibit melanoma cell survival and tumour growth. **a** A375 cells seeded at low density were treated with DMSO control (C), 2 μM vemurafenib (Vem), 1 μM AZD5991 or the combination for 72 h. **b** A375 cells seeded at low density were treated with DMSO control (C), 1 μM selumetinib (Sel), 1 μM AZD5991 or the combination for 72 h. **c** A375 cells seeded at low density were treated with DMSO control (C), 2 μM vemurafenib (Vem), 1 μM AZD4320 or the combination for 72 h. **a**–**c** Following treatment cells were washed and grown in inhibitor free medium for a further four days. Colonies were then visualised by crystal violet staining and counted and then growth assessed following solubilisation. Results are mean ± SD of three or more independent experiments. *P* ≤ 0.0001 (****), *P* ≤ 0.05 (*) or ns (not significant) as determined by one-way ANOVA and Sidak’s multiple comparisons test. **d**, **e** Female nude athymic mice were implanted subcutaneously with A375 cells and randomised 21 days later for dosing with either vehicle-only (Control, *n* = 5), 25 mg kg^−1^ selumetinib (Sel, *n* = 7) twice daily with an 8 h interval, 60 mg kg^−1^ AZD5991 (*n* = 7) intravenously once weekly, or the combination of 25 mg kg^−1^ selumetinib and 60 mg kg^−1^ AZD5991 (Sel + AZD5991, *n* = 8) for a further 21 days. Tumour growth was recorded twice weekly and results are mean ± SEM (**d**) or mean % change in tumour volume ± SEM (**e**). *P* ≤ 0.0001 (****), *P* ≤ 0.001 (***) or *P* ≤ 0.05 (*), as determined by two-way ANOVA and Holm-Sidak’s multiple comparisons test

Combining vemurafenib or selumetinib with AZD5991 also inhibited the clonogenic survival of A375 cells (Fig. 3a, b). However, combined vemurafenib and AZD4320 or navitoclax did not significantly inhibit colony formation (Fig. 3c and Supplementary Fig. 8a).

The striking synergy between ERK1/2 pathway inhibition and AZD5991 in melanoma was also observed in vivo. While selumetinib moderately inhibited the growth of A375 tumour xenografts, and AZD5991 had no effect, selumetinib + AZD5991 caused regression of established tumours and durable full growth inhibition (Fig. 3d, e and Supplementary Fig. 8b, c). Given that AZD5991 is cleared quickly from blood plasma, within 2–6 h^33^, this efficacy is particularly striking in the context of a once weekly AZD5991 dosing schedule that likely results in MCL1 inhibition for just a few hours per week. Throughout dosing there was little change in mouse body mass indicating that these treatments were well tolerated (Supplementary Fig. 8d).

**Fig. 4:**
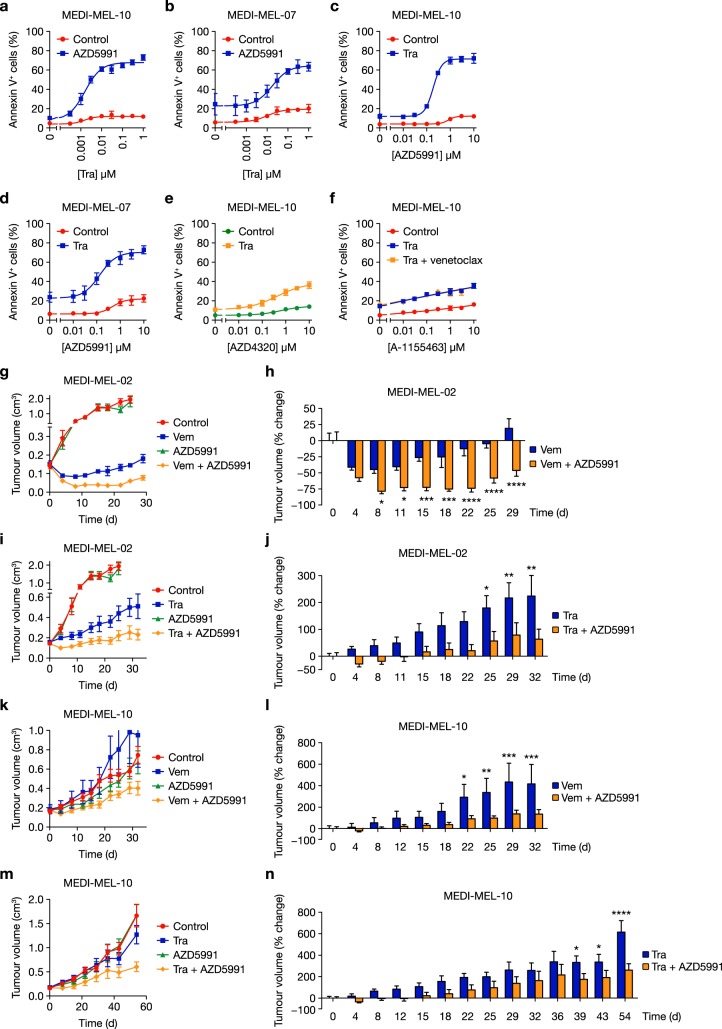
BRAFi or MEKi combine with AZD5991 to inhibit growth of patient-derived xenografts. **a**, **b** MEDI-MEL-10 (**a**) and MEDI-MEL-07 (**b**) PDX cell lines were treated with increasing concentrations of trametinib (Tra) with or without 1 μM AZD5991 for 72 h. **c**, **d** MEDI-MEL-10 (**c**) or MEDI-MEL-07 (**d**) cells were treated with increasing concentrations of AZD5991 with or without 0.3 μM trametinib (Tra) for 72 h. **e** MEDI-MEL-10 cells were treated with increasing concentrations of AZD4320 with or without 0.3 μM trametinib (Tra) for 72 h. **f** MEDI-MEL-10 cells were treated with increasing concentrations of A-1155463 with or without 0.3 μM trametinib (Tra) and 3 μM venetoclax for 72 h. **a**–**f** Apoptosis was assessed by Annexin V positivity using flow cytometry. Results are mean ± SD of three or more independent experiments. **g**–**n** NOD SCID mice were implanted subcutaneously with MEDI-MEL-02 (**g**–**j**) or MEDI-MEL-10 (**k**–**n**) tumour pieces. Upon reaching 0.15–0.2 cm^3^ mice were randomised by tumour volume and body mass for dosing (*n* = 10 per group for MEDI-MEL-02, *n* = 9 per group for MEDI-MEL-10) with vehicle-only (Control), 20 mg kg^−1^ vemurafenib (Vem) twice daily with an 8 h interval, 1 mg kg^−1^ trametinib (Tra) daily, and/or 60 mg kg^−1^ AZD5991 three times per week, as indicated. Mice were dosed on these schedules throughout the duration of the experiment, except in **m** and **n** for which dosing ceased after day 32. Tumour growth was recorded twice weekly and results are mean ± SEM (**g**, **i**, **k**, **m**) or mean % change in tumour volume ± SEM (**h**, **j**, **l**, **n**). *P* ≤ 0.0001 (****), *P* ≤ 0.001 (***), *P* ≤ 0.01 (**) or *P* ≤ 0.05 (*), as determined by two-way ANOVA and Holm-Sidak’s multiple comparisons test

We next examined BRAF^V600E^-mutant primary patient-derived melanoma cells. Consistent with the results described above, trametinib and AZD5991 combined to kill MEDI-MEL-10 and MEDI-MEL-07 primary melanoma cells (Fig. 4a–d), whereas combination with AZD4320 or A-1155463 was far less effective (Fig. 4e, f). Thus, dependency on MCL1 when the ERK1/2 pathway was inhibited was observed in melanoma cell lines and primary patient-derived melanoma cells.

BRAFi or MEKi combinations with AZD5991 were also effective in BRAF-mutant PDX models (Fig. 4g–n and Supplementary Fig. 9a–f). While the BRAFi vemurafenib induced moderate initial regressions of BRAF^V600E^-mutant MEDI-MEL-02 PDX tumours, combination with AZD5991 caused sustained near-complete tumour regressions (Fig. 4g, h and Supplementary Fig. 9a). Similarly, AZD5991 substantially enhanced tumour growth inhibition by trametinib (Fig. 4i, j and Supplementary Fig. 9b). In another PDX model, BRAF^V600E^-mutant MEDI-MEL-10, AZD5991 again enhanced the efficacy of vemurafenib or trametinib despite this model being refractory to these ERK1/2 pathway inhibitors as monotherapies (Fig. 4k–n). These PDX models represented extremes of response to ERK1/2 pathway inhibition; MEDI-MEL-02 tumour growth was halted by vemurafenib or trametinib, whereas MEDI-MEL-10 tumours were unresponsive (Fig. 4g–n). However, AZD5991 was able to combine with BRAFi or MEKi to regress or inhibit tumour growth and these combinations were well tolerated (Supplementary Fig. 9g–j).

Altogether these results indicate that the melanoma bias towards MCL1 provides a therapeutic opportunity for combination with multiple ERK1/2 pathway inhibitors, including the clinically approved BRAF^V600^-mutant selective inhibitor vemurafenib and MEKi such as selumetinib or trametinib, thereby promoting striking tumour cell apoptosis and enhancing tumour growth inhibition in vivo.

### BRAFi or MEKi prime melanoma for MCL1i-induced death

**Fig. 5:**
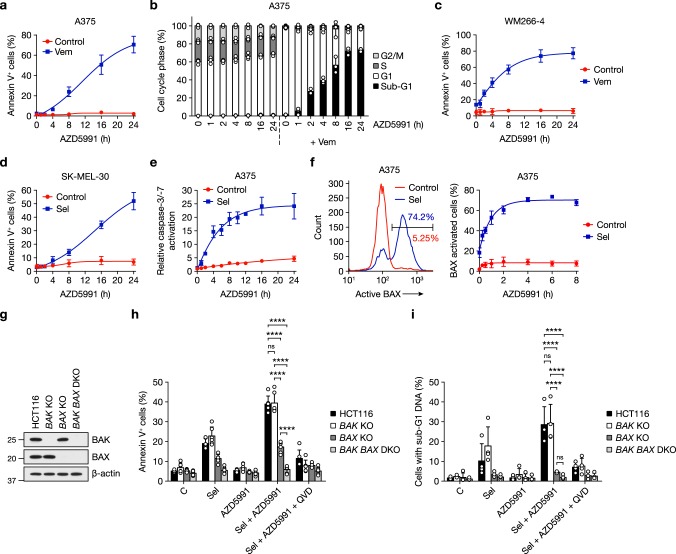
ERK1/2 pathway inhibitors prime melanoma cells for rapid apoptosis following MCL1i. **a**, **b** A375 cells were treated with DMSO vehicle-only (Control) or 2 μM vemurafenib (Vem) for 24 h as indicated. One micromolar AZD5991 was then added for the indicated times. Apoptosis was assessed by Annexin V flow cytometry (**a**), or cells fixed, stained with propidium iodide and cell cycle profile determined by flow cytometry (**b**). **c**, **d** WM266-4 (**c**) and SK-MEL-30 (**d**) cells were treated with DMSO vehicle-only (Control), 1 μM vemurafenib (Vem) or 1 μM selumetinib (Sel) for 24 h as indicated. One micromolar AZD5991 was then added for the indicated times and apoptosis assessed by Annexin V staining and flow cytometry. **e** A375 cells were treated with DMSO vehicle-only (Control) or 1 μM selumetinib (Sel) for 24 h. One micromolar AZD5991 was then added for the indicated times and relative caspase-3/-7 activity measured. **f** A375 cells were treated with DMSO vehicle-only (Control) or 1 μM selumetinib (Sel) for 24 h followed by combination with 1 μM AZD5991 for the indicated times. BAX activation was determined using anti-BAX antibody clone 6A7, an N-terminal antibody specific for an active conformation of BAX, and flow cytometry. A representative flow cytometer trace is shown (left) alongside pooled results (right). **g** HCT116, HCT116 *BAK* KO, HCT116 *BAX* KO and HCT116 *BAK BAX* DKO isogenic cell lines were lysed and western blotted with the indicated antibodies. **h**, **i** HCT116, HCT116 *BAK* KO, HCT116 *BAX* KO and HCT116 *BAK BAX* DKO isogenic cell lines were treated with DMSO control (C), 2 μM selumetinib (Sel), 1 μM AZD5991 or 2 μM selumetinib plus 1 μM AZD5991 (Sel + AZD5991) with or without 20 μM Q-VD-OPh (Sel + AZD5991 + QVD) for 48 h, and cell death assessed by Annexin V (**h**) or PI (**i**) staining and flow cytometry. Results (**a**–**f**, **h**, **i**) are mean ± SD of three or more independent experiments. *P* ≤ 0.0001 (****) and ns (not significant) as determined by two-way ANOVA and Tukey’s multiple comparisons test

To define the mechanisms underpinning synergy between ERK1/2 pathway inhibition and AZD5991 we assessed the kinetics of the apoptotic response. Addition of AZD5991 to A375 cells pre-treated with vemurafenib or selumetinib for 24 h resulted in a time-dependent increase in annexin V-positive cells (Fig. 5a and Supplementary Fig. 10a). This rapid induction of apoptosis was even more apparent at the level of DNA cleavage, suggesting that DNA fragmentation preceded phosphatidylserine membrane leaflet flipping (Fig. 5b). Similar rapid apoptosis also occurred following addition of AZD5991 to WM266-4 or SK-MEL-30 cells pre-treated with vemurafenib or selumetinib, respectively (Fig. 5c, d).

Accumulation of annexin V-positive cells was preceded by caspase activation. Selumetinib pre-treatment of A375 cells for 24 h, or treatment with AZD5991 alone, had little effect on caspase-3/-7 activity. However, in cells pre-treated with selumetinib, caspase-3/-7 activity increased ~5-fold after 2 h AZD5991 treatment, reaching a plateau (~20-fold increase) after 8 h (Fig. 5e). Caspase activation was preceded by activation of the key apoptotic effector BAX, assessed using the 6A7 antibody that recognises an amino-terminal epitope exposed only when BAX is activated (Fig. 5f). In A375 cells pre-treated with selumetinib for 24 h, AZD5991 caused activation of BAX within 15 min that reached a plateau after 2–4 h (Fig. 5f). Thus, ERK1/2 pathway inhibitors primed ERK1/2-dependent tumour cells to undergo an immediate activation of the canonical cell intrinsic apoptotic pathway following MCL1 inhibition.

To examine whether apoptosis required *BAK*, *BAX* or both, we utilised isogenic HCT116 cells lacking either *BAK*, *BAX* or both (*BAK BAX* DKO)^38^ (Fig. 5g). Although HCT116 is a CRC cell line, a combination effect with AZD5991 is still observed, albeit more modest than that observed with A375 or SK-MEL-30. *BAK* knock-out (KO) cells showed no apoptotic defect in response to either selumetinib or combination with AZD5991 (Fig. 5h), whereas cells lacking *BAX* exhibited a 60% reduction in apoptosis. Finally, *BAK BAX* double knock-out (DKO) cells were completely resistant to the modest pro-apoptotic effects of selumetinib or the more striking response to combined selumetinib + AZD5991 (Fig. 5h). So while BAX was the more important effector in HCT116, there was some redundancy with BAK. Regardless, the combination killed cells in a BAK/BAX-dependent manner, confirming the anticipated on-target mechanism of this drug combination. In addition, this combination did not increase the fraction of necrotic cells, only apoptotic cells, and *BAX* knock-out also prevented the accumulation of cells with sub-G1 DNA (Fig. 5i and Supplementary Fig. 10b).

### BRAFi or MEKi plus MCLi drives BIM- and BMF-dependent apoptosis

**Fig. 6:**
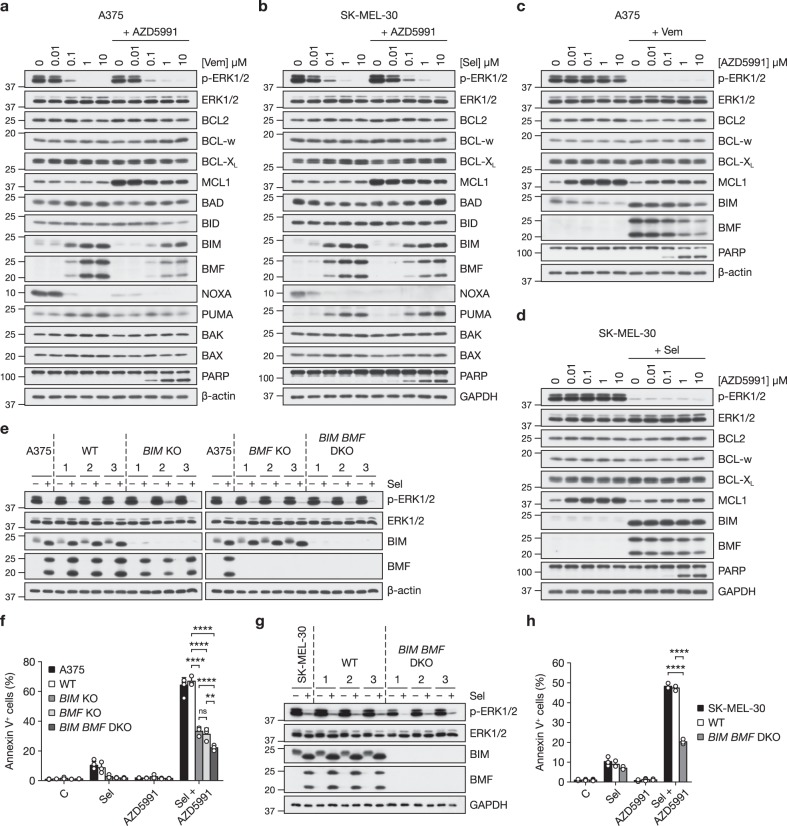
Apoptosis induced by combined ERK1/2 pathway and MCL1 inhibition requires BIM and BMF. **a** A375 cells were treated with the indicated concentrations of vemurafenib (Vem) with or without 1 μM AZD5991 for 24 h. **b** SK-MEL-30 cells were treated with the indicated concentrations of selumetinib (Sel) with or without 1 μM AZD5991 for 24 h. **c** A375 cells were treated with the indicated concentrations of AZD5991 with or without 1 μM vemurafenib (Vem) for 24 h. **d** SK-MEL-30 cells were treated with the indicated concentrations of AZD5991 with or without 1 μM selumetinib (Sel) for 24 h. **a**–**d** Whole-cell lysates were western blotted with the indicated antibodies. **e**, **f** A375 cells and three independent clonally derived WT, *BIM* KO, *BMF* KO and *BIM BMF* DKO A375 CRISPR clones were treated with 1 μM selumetinib (Sel) as indicated for 24 h and BIM and BMF expression assessed by western blotting (**e**) or treated with DMSO-only control (C), 1 μM selumetinib (Sel), 1 μM AZD5991 or 1 μM selumetinib plus 1 μM AZD5991 (Sel + AZD5991) as indicated for 48 h and apoptosis assessed by Annexin V staining and flow cytometry (**f**). Results (**f**) show mean ± SD of three independent experiments. *P* ≤ 0.0001 (****), *P* ≤ 0.01 (**) and ns (not significant) as determined by two-way ANOVA and Tukey’s multiple comparisons test. **g**, **h** SK-MEL-30 cells and three independent clonally derived WT and *BIM BMF* DKO SK-MEL-30 CRISPR clones were treated with 1 μM selumetinib (Sel) as indicated for 24 h and BIM and BMF expression assessed by western blotting (**g**) or treated with DMSO-only control (C), 1 μM selumetinb (Sel), 1 μM AZD5991 or 1 μM selumetinib plus 1 μM AZD5991 (Sel + AZD5991) as indicated for 48 h and apoptosis assessed by Annexin V staining and flow cytometry (**h**). Results (**h**) show mean ± SD of three independent experiments. *P* ≤ 0.0001 (****) as determined by two-way ANOVA and Tukey’s multiple comparisons test

We next examined how ERK1/2 pathway inhibition and/or AZD5991 influenced expression of the BCL2 protein family, the upstream regulators of BAK and BAX. Treatment of A375 cells with vemurafenib (Fig. 6a) or selumetinib (Supplementary Fig. 11a) increased expression of the pro-apoptotic BH3-only proteins BIM and BMF in a concentration-dependent manner as expected^11^. Inhibition of ERK1/2 also abolished expression of NOXA, consistent with observations that *NOXA* mRNA expression is promoted by ERK1/2 signalling^39,40^. Similar results were observed in SK-MEL-30 cells with the MEKi selumetinib (Fig. 6b) and these cells also exhibited PUMA induction. BRAFi or MEKi had more modest effects on other pro-apoptotic and pro-survival BCL2 proteins (Fig. 6a, b and Supplementary Fig. 11a). In both cell lines, 1 µM AZD5991 increased the abundance of its target, MCL1, and caused loss of NOXA expression (Fig. 6a, b). The increase in MCL1 expression likely reflects its stabilisation because MULE, an E3 ubiquitin ligase for MCL1, binds to MCL1 via a BH3 domain^41^ and so will be displaced by AZD5991; thus, increases in MCL1 expression serve as a good marker for MCL1 engagement by AZD5991. The reduction in NOXA may also reflect its displacement from MCL1; certainly some BH3-only proteins are intrinsically disordered and undergo proteasome-dependent turnover when displaced from their binding partners^42,43^. Combination of vemurafenib (Fig. 6c) or selumetinib (Supplementary Fig. 11b) with increasing concentrations of AZD5991 promoted PARP cleavage indicative of caspase-3 activation, and also reduced expression of BIM and BMF slightly compared to ERK1/2 pathway inhibitor alone. In A375 and SK-MEL-30 cells, MCL1 expression increased with AZD5991 concentrations as low as 10 nM (Fig. 6c, d and Supplementary Fig. 11b).

These results suggested that BIM and/or BMF might be responsible for initiating apoptosis in response to combined inhibition of MCL1 and ERK1/2 signalling so we used CRISPR/Cas9 gene editing to generate *BIM* KO, *BMF* KO or double knock-out (*BIM BMF* DKO) A375 cells (Fig. 6e). We selected three independent clones for each genotype and also three wild-type clones that had been through CRISPR/Cas9 editing with guides for *BIM*, *BMF* or both but were negative clones, retaining normal expression of the targeted proteins and serving as controls for off-target effects. These clones were compared with parental A375 cells (Fig. 6f). We found that the striking cell death induced by selumetinib + AZD5991 was inhibited ~50% by knock-out of *BIM* or *BMF* whereas *BIM BMF* DKO cells exhibited a 65% reduction in cell death (Fig. 6f). Thus, BIM and BMF make significant contributions to cell death arising from selumetinib + AZD5991, likely acting through a shared mechanism. The remaining cell death response might reflect contributions from PUMA and BAD, which exhibited modest increases in abundance in response to vemurafenib (Fig. 6a) or selumetinib (Supplementary Fig. 11a). A very similar dependency upon BIM and BMF was observed in SK-MEL-30 cells. Knock-out of *BIM* and *BMF* (*BIM BMF* DKO) (Fig. 6g) inhibited apoptosis induced by selumetinib + AZD5991 by 60–70% compared to parental cells and wild-type clones that, as above, retained normal BIM and BMF expression and induction following the CRISPR/Cas9 process (Fig. 6g, h).

**Fig. 7:**
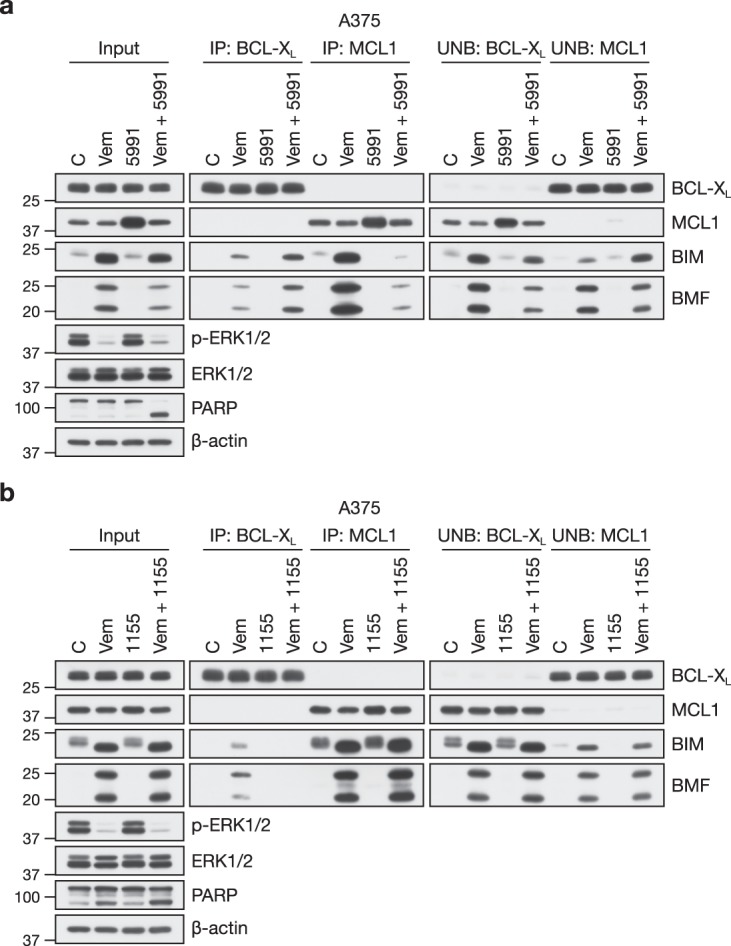
Combined BRAFi and MCL1i redistributes BIM and BMF to other pro-survival proteins. **a** A375 cells were treated with DMSO vehicle-only (C and 5991) or 2 μM vemurafenib (Vem and Vem + 5991) for 24 h followed by DMSO-only (C and Vem) or 1 μM AZD5991 (5991 and Vem + 5991) for a further 4 h. Lysates (input) were subjected to immunoprecipitation with antibodies to BCL-X_L_ or MCL1. Input lysates, immunoprecipitates (IP) and supernatant unbound fractions (UNB) were then western blotted using the indicated antibodies. **b** A375 cells were treated with DMSO vehicle-only (C and 1155) or 2 μM vemurafenib (Vem and Vem + 1155) for 24 h followed by DMSO-only (C and Vem) or 0.1 μM A-1155463 (1155 and Vem + 1155) for a further 4 h. Lysates (input) were subjected to immunoprecipitation with antibodies to BCL-X_L_ or MCL1. Input lysates, immunoprecipitates (IP) and supernatant unbound fractions (UNB) were then western blotted using the indicated antibodies

Binding of AZD5991 to MCL1 should displace BH3-only proteins such as BIM and BMF from MCL1, allowing them to inhibit other pro-survival proteins to drive apoptosis. To test this we treated A375 cells with vemurafenib, AZD5991 or the combination and then immunoprecipitated (IP) either MCL1 or BCL-X_L_ before blotting for the presence of BIM and BMF (Fig. 7a). Vemurafenib increased expression of BIM and BMF and while these bound to both MCL1 and BCL-X_L_, far more bound to MCL1 than to BCL-X_L_ (Fig. 7a), consistent with the high MCL1:BCL-X_L_ ratio (~8) in these cells (Fig. 1 and Supplementary Fig. 2). AZD5991 increased MCL1 abundance, as seen in all experiments, but did not affect BIM or BMF expression. Combination with AZD5991 slightly reduced the vemurafenib-driven increase in BIM and BMF abundance. Most strikingly, vemurafenib + AZD5991 promoted a loss of BIM and BMF from MCL1 and their binding to BCL-X_L_ (Fig. 7a); given the decrease in total BIM and BMF this is likely an underestimate of the redistribution of these proteins to BCL-X_L_. Very similar results were observed when selumetinib and AZD5991 were combined in A375 and SK-MEL-30 cells (Supplementary Fig. 11c, d). Thus, AZD5991 acted on-target to drive redistribution of BIM and BMF from MCL1 on to other pro-survival BCL2 proteins, including BCL-X_L_, which are not themselves targets of AZD5991. Notably, since BIM may activate BAX directly as well as indirectly through inhibition of pro-survival proteins^44,45^, combination of vemurafenib or selumetinib with AZD5991 also increased the amount of free unbound BIM (Fig. 7a and Supplementary Fig. 11c, d).

In contrast to MCL1 inhibition by AZD5991, we observed minimal redistribution of BIM and BMF when BCL-X_L_ was inhibited by A-1155463 in conjunction with ERK1/2 signalling in A375 cells, consistent with the high MCL1:BCL-X_L_ ratio in these cells (~8) (Figs. 1 and 7b and Supplementary Fig. 2). The BCL-X_L_ inhibitor A-1155463 displaced BIM and BMF from BCL-X_L_, but caused little accumulation on to MCL1 or increase in the fraction of free BIM and BMF not bound to BCL-X_L_ (Fig. 7b). This was not due to loss of BIM and BMF expression in the presence of the combination; indeed, BIM and BMF expression was only reduced in combinations that caused strong PARP cleavage and cell death (Fig. 7a, b and Supplementary Fig. 11c, d). Thus, this is consistent with a more minor role for BCL-X_L_ in these melanoma cells.

Taken together these results indicate AZD5991 acts on-target in melanoma to inhibit MCL1 and strongly synergises with ERK1/2 pathway inhibitors that drive BIM and BMF expression to promote BAK/BAX-dependent apoptosis; this arises through redistribution of BIM and BMF from MCL1 on to other pro-survival proteins and an increase in free BIM, which may directly activate BAX.

### ERKi plus MCL1i overcomes BRAFi and/or MEKi resistance

**Fig. 8:**
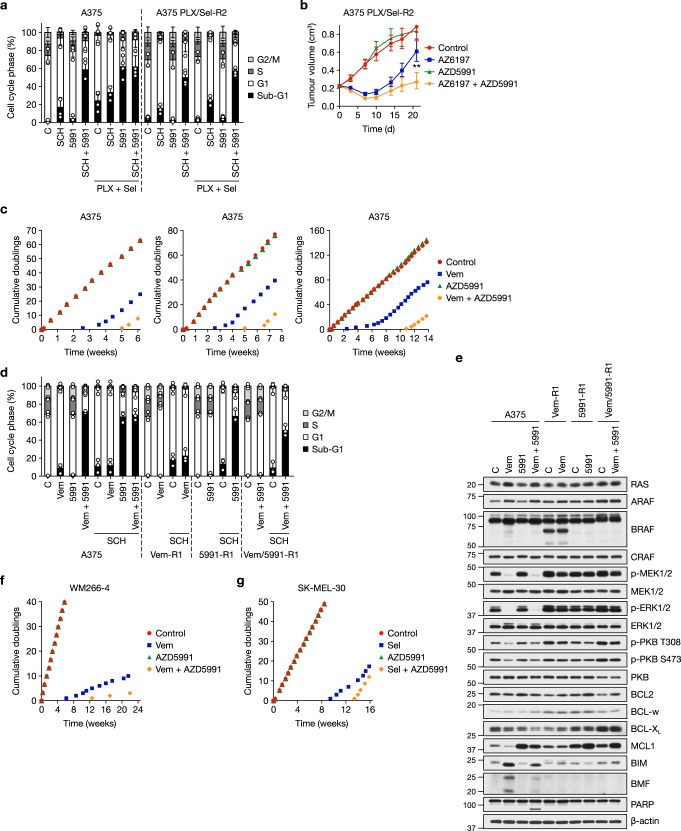
AZD5991 can delay and overcome acquired resistance to BRAFi and/or MEKi. **a** A375 and PLX4720/selumetinib-resistant A375 (A375 PLX/Sel-R2) cells were washed and treated with 0.3 μM SCH772984 (SCH), 1 μM AZD5991 (5991) or the combination (SCH + 5991) for 48 h, either in the presence (PLX + Sel) or the absence of 0.66 μM PLX4720 and 0.5 μM selumetinib, their normal growth medium. Cell cycle profile was determined by propidium iodide staining and flow cytometry. Results show mean ± SD of four independent experiments. **b** Female athymic nude mice were injected subcutaneously with A375 PLX/Sel-R2 cells. Once tumours reached an average of 0.2 cm^3^_,_ mice were randomised in to treatment groups and dosed with vehicle-only (Control, *n* = 10), 50 mg kg^−1^ AZ13776197 (AZ6197, *n* = 10) orally once daily, 60 mg kg^−1^ AZD5991 (AZD5991, *n* = 10) once weekly intravenously, or the combination of 50 mg kg^−1^ AZ6197 and 60 mg kg^−1^ AZD5991 (AZ6197 + AZD5991, *n* = 10). Tumour volume growth results are geometric mean ± SEM. *P* ≤ 0.01 (**) as determined by two-way ANOVA and Holm-Sidak’s multiple comparisons test. **c** A375 cells were cultured in the presence of DMSO-only (Control), 2 μM vemurafenib (Vem), 1 μM AZD5991 or the combination (Vem + AZD5991) and the number of cumulative doublings recorded. Three independent experiments are shown. **d** A375, vemurafenib-resistant A375 (Vem-R1) cells, AZD5991-resistant A375 (5991-R1) cells and vemurafenib/AZD5991-resistant A375 (Vem/5991-R1) cells were treated with 2 μM vemurafenib (Vem), 1 μM AZD5991 (5991) with or without 1 μM SCH772984 (SCH) as indicated for 48 h. Cell cycle profile was determined by propidium iodide staining and flow cytometry. Results show mean ± SD of three independent experiments. **e** A375, Vem-R1, 5991-R1 and Vem/5991-R1 were washed and treated with 2 μM vemurafenib (Vem) and/or 1 μM AZD5991 (5991) as indicated for 24 h. Whole-cell lysates were western blotted with the indicated antibodies. **f** WM266-4 cells were cultured in the presence of DMSO-only (Control), 1 μM vemurafenib, 1 μM AZD5991 or Vem + AZD5991 and the number of cumulative doublings recorded. **g** SK-MEL-30 cells were cultured in the presence of DMSO-only (Control), 1 μM selumetinib (Sel), 1 μM AZD5991 or Sel + AZD5991 for 16 weeks and the number of cumulative doublings recorded

Our results indicated that AZD5991 would combine very well with BRAFi and/or MEKi as a primary, up-front drug combination but we also assessed activity in models of acquired resistance. BRAFi + MEKi is now the standard for consistency care for melanoma with BRAF^V600E/K^ ^6,7^; however, acquired resistance still emerges, typically through reactivation of ERK1/2, and this is fuelling the development of ERK1/2 inhibitors^46^. We generated A375 sub-lines with acquired resistance to PLX4720 (a vemurafenib analogue) (Supplementary Fig. 12a), selumetinib (Supplementary Fig. 12b) or the combination of PLX4720 + selumetinib at five- or 10-times the IC_50_ concentrations (Fig. 8a and Supplementary Fig. 12c). Next-generation sequencing revealed that PLX4720/selumetinib-resistant A375 sub-lines exhibited NRAS^Q61R^ and MEK1^Q56P^ mutations (Supplementary Fig. 12d–g). Whereas BRAF copy number and expression were unchanged in both PLX4720/selumetinib-resistant A375 sub-lines, selumetinib-resistant A375 cells harboured *BRAF* amplification and upregulation of BRAF expression, whereas PLX4720-resistant cells expressed a putative splice variant of BRAF^V600E^ (Supplementary Fig. 13a–f); these are well defined mechanisms of resistance to MEKi or BRAFi, respectively^5,47–49^. In each case these mechanisms reinstated ERK1/2 signalling in the presence of the respective inhibitor (Supplementary Fig. 13e); however, ERK1/2 signalling in these resistant sub-lines remained sensitive to the selective ERK1/2 inhibitor SCH772984 (Supplementary Fig. 13f). This ERK1/2 inhibitor again combined well with AZD5991 to kill parental A375 cells (Fig. 8a and Supplementary Fig. 12a–c). Significantly, this cell death response was fully maintained in PLX4720/selumetinib-resistant A375 cells (Fig. 8a and Supplementary Fig. 12c), as well as the cell lines resistant to PLX4720 (Supplementary Fig. 12a) or selumetinib (Supplementary Fig. 12b) monotherapy.

To see if this activity of ERKi + MCL1i was maintained in vivo we employed tumour xenografts using the PLX4720/selumetinib-resistant A375 cells. For these studies we used the ERKi AZ6197^50^, due to the poor pharmacokinetic properties of SCH772984. AZD5991 had no activity as monotherapy against the PLX4720/selumetinib-resistant A375 cells (Fig. 8b), as was seen in cell culture studies (Fig. 8a). AZ6197 caused significant inhibition of tumour growth, consistent with ERKi being able to overcome acquired resistance to BRAFi + MEKi, but this effect was transient. AZD5991 combined with AZ6197 to cause a more profound and durable tumour growth inhibition (Fig. 8b). Again this was striking given that AZD5991 was dosed once weekly, a schedule that likely results in MCL1 engagement for just a few hours per week. AZ6197 alone, or in combination with AZD5991, was well tolerated (Supplementary Fig. 13g). These results indicate that, as well as use in an up-front combination with BRAFi and/or MEKi, AZD5991 can be used in combination with an ERKi to overcome acquired resistance to BRAFi and/or MEKi driven by a variety of mutations in ERK1/2 pathway components.

Despite the efficacy of AZD5991 in these combination studies experience shows that acquired resistance is inevitable. To model this we generated A375 cells adapted to grow in vemurafenib (Vem-R), AZD5991 (5991-R) or the combination (Vem/5991-R) in three independent experiments. Cumulative population doublings showed that AZD5991 had no effect on A375 cell proliferation (Fig. 8c). Vemurafenib caused a complete cessation of A375 proliferation for ~2.5-weeks before resistant cells started to emerge and proliferate whereas combination with AZD5991 doubled the time to resistance, with Vem/5991-R-resistant cells emerging after 5–10 weeks (Fig. 8c, d). Vem-R cells exhibited a previously described BRAF splice variant that likely allowed them to maintain p-MEK1/2 and p-ERK1/2 in the presence of vemurafenib^49^ (Fig. 8e). Interestingly 5991-R cells exhibited higher levels of MCL1, though this was reduced slightly following withdrawal of AZD5991, suggesting that this might simply represent AZD5991 binding and stabilisation of MCL1 as described above. Vem/5991-R A375 cells did not express any apparent BRAF splice variant, suggesting that this drug combination selected for reinstatement of ERK1/2 signalling through another mechanism (Fig. 8e). Significantly, all resistant cells, whether to monotherapy or combination, retained p-MEK1/2 and p-ERK1/2, and all Vem-R, 5991-R and Vem/5991-R remained sensitive to ERKi SCH772984 (Fig. 8d and Supplementary Fig. 14a, b). Thus, adaptation or acquired resistance to vemurafenib, AZD5991 or vemurafenib + AZD5991 could be overcome by combination with an ERK1/2 inhibitor.

Combination with AZD5991 also doubled the time taken for vemurafenib resistance to emerge in WM266-4 cells (Fig. 8f), and increased the time to selumetinib resistance in SK-MEL-30 cells by ~50% (Fig. 8g and Supplementary Fig. 14c). As with A375 cells, SK-MEL-30 cells acquired resistance to selumetinib monotherapy and selumetinib combination with AZD5991 by reinstating p-MEK1/2 and p-ERK1/2 in the presence of inhibitor (Supplementary Fig. 14d), and similarly acquired resistance to selumetinib or selumetinib + AZD5991 could be overcome using the ERK1/2 inhibitor SCH772984 (Supplementary Fig. 14c). Thus, AZD5991 can delay acquired resistance to BRAFi or MEKi in BRAF-mutant or NRAS-mutant melanoma cells.

## Discussion

While the BRAFi + MEKi combination has transformed the treatment of BRAF^V600E/K^-mutant melanoma, clinical responses are transient due to the emergence of resistance. This reflects the fact that even strong ERK1/2 inhibition exerts a predominantly cytostatic effect despite causing a substantial increase in pro-apoptotic BIM, BMF and in some cases PUMA. This is also true in other tumour lineages, such as CRC; however, whereas increased BIM and BMF primed CRC cells to undergo substantial apoptosis following treatment with the BCL2/BCL-w/BCL-X_L_ inhibitor navitoclax/ABT-263^11,25,26^, this combination was notably less effective in melanoma^11,27,28^. This prompted us to examine the expression of pro-survival proteins. Interrogation of CCLE and TCGA data sets indicated that the median *MCL1*:*BCL-X*_*L*_ mRNA ratio was two- to fourfold higher in melanoma than in CRC, NSCLC and pancreatic cancer lineages. This was confirmed by western blotting and absolute protein quantification, which revealed that low BCL-X_L_ expression biased the pro-survival protein pool strongly towards MCL1 in melanoma.

Our results reveal that this lineage-selective MCL1 bias predicts the response to the MCL1i AZD5991, but only in combination with agents that inhibit ERK1/2 signalling to drive BIM and BMF expression. MCL1i monotherapy, even at higher concentrations, induced little apoptosis of melanoma (or CRC) tumour cells in vitro and did not inhibit cell division during long-term culture, while in vivo AZD5991 alone did not inhibit the growth of melanoma xenografts. Therefore, melanoma tumour cells are not dependent on MCL1 for survival per se; rather they are dependent on MCL1, to a far greater extent than the other pro-survivals, when the ERK1/2 pathway is inhibited. Presumably, without induction of BIM and/or BMF the basal level of other pro-survival proteins represents a sufficient barrier to prevent apoptosis when MCL1 is inhibited.

These results suggest that melanoma represents a unique opportunity as a disease in which two broad therapeutic windows (ERK1/2 addiction and MCL1 addiction) are inter-dependent, with MCL1 addiction only manifest when ERK1/2 signalling is inhibited. This synthetic lethality will be best leveraged using BRAF inhibitors such as vemurafenib, which specifically inhibit ERK1/2 signalling in BRAF^V600^-mutant tumour cells, but may also transform the potential of MEKi and ERKi in ERK1/2-addicted melanoma tumour cells. This is reflected in striking synergistic cell death in vitro and tumour regression or enhanced growth inhibition in vivo when BRAFi or MEKi and AZD5991 are combined as an up-front combination.

Mechanistic studies demonstrated that ERK1/2 inhibition primes melanoma cells to undergo apoptosis following subsequent MCL1 inhibition. This apoptosis required increased expression of BIM and BMF, which appear to play overlapping roles; the remaining 20–25% cell death in *BIM BMF* DKOs may reflect contributions from BAD and PUMA, both of which increased in expression following ERK1/2 inhibition. Apoptosis following addition of AZD5991 to primed cells was rapid, with maximal BAX activation after 2–4 h, underscoring the extent to which these cells are addicted to MCL1 to restrain the BIM and BMF induced by ERK1/2 inhibition. Consistent with this, when MCL1 was inhibited we observed redistribution of BIM and BMF from MCL1 on to BCL-X_L_ and an increase in free unbound BIM that may activate BAX directly^44,45^. NOXA exhibits highly selective binding to MCL1 (and A1) vs. BCL2, BCL-w or BCL-X_L_^11,51,52^, and consistent with previous reports was dramatically lost when the ERK1/2 pathway was inhibited (Fig. 6). This loss of NOXA presumably dampens the apoptotic response to ERK1/2 pathway inhibitor monotherapy but further primes MCL1 with BIM and BMF. Release of this BIM and BMF upon inhibition of MCL1 may therefore accentuate the synergistic effect of combined ERK1/2 pathway and MCL1 inhibition.

Critically, AZD5991 was also active in melanoma cells with acquired resistance to BRAFi + MEKi, the current standard of care for BRAF^V600E/K^ melanoma. These cells acquired resistance through the emergence of NRAS and MEK1 mutations to reinstate ERK1/2 signalling and remained sensitive to ERKi. Significantly ERKi still combined with MCL1i to kill BRAFi + MEKi-resistant tumour cells in vitro and inhibit their growth as tumour xenografts in vivo.

Finally, AZD4320 (a BCL2/BCL-w/BCL-X_L_ inhibitor) synergised with ERK1/2 pathway inhibitors to induce apoptosis more strongly than AZD5991 in CRC, a disease with common KRAS or BRAF mutations that is as yet poorly served by ERK1/2 pathway inhibition. The reciprocal was true in melanoma, where combination with AZD5991 generally resulted in greater synergy and apoptosis than combination with AZD4320. Despite high expression of BCL2 in melanoma compared to other lineages, the response to AZD4320 was not replicated by selective BCL2 inhibition with venetoclax, but rather was recapitulated using the selective BCL-X_L_ inhibitor A-1155463. Thus, although either BCL2/BCL-w/BCL-X_L_ or MCL1 inhibitors can synergise to some extent with ERK1/2 pathway inhibitors in a broad range of lineages^11,26,27,53,54^, the ability to target individual pro-survival proteins as BH3-mimetic-specificity continues to be refined will allow antagonism of only those critical pro-survival proteins that limit apoptotic responses to ERK1/2 pathway inhibitors in a particular lineage, thereby improving the therapeutic window of these combinations. Whereas BRAFi and MEKi are approved for use in melanoma, current clinical studies are investigating how best to combine BRAFi and/or MEKi with other targeted agents in CRC and other solid tumours such as NSCLC and pancreatic^55,56^. Our results suggest that in CRC ERK1/2 inhibition may combine better with antagonists of BCL-X_L_ than MCL1 due to their relative expression, further underlining the potential for tumour lineage-selective differences that will drive BH3-mimetic combination choices; as we show here, the ratio of pro-survival proteins will be informative in this choice, and represent a potential clinical biomarker.

In summary, our results demonstrate that targeting melanoma’s MCL1 bias unleashes the potential of BRAF and ERK1/2 pathway inhibitors by transforming cytostatic responses into striking apoptotic cell death. This approach results in superior tumour regressions or growth inhibition and could afford a broad therapeutic window in patients. Since BRAFi and MEKi are already approved for treatment of BRAF^V600E/K^-positive melanoma, combination with MCL1i may provide an opportunity to promptly improve patient outcomes.

## Methods

### Reagents and resources

A full list of reagents and resources used in this study is provided in Supplementary Table 3. Further information and requests for resources and reagents should be directed to Simon Cook (simon.cook@babraham.ac.uk).

### Cell lines and culture

Human colorectal cancer (CRC) cell lines Caco-2, COLO205, DLD-1, HT29, LS174T, LS411N, NCI-H747, SK-CO-1, SW48, SW480, SW620, SW1116 and SW1417 were from ATCC (LGC Standards, Middlesex, UK). HCA7, HCT8 and SW837 cells were from ECACC (Culture Collections, Public Health England, Salisbury, UK). CO115 cells were provided by Dr. Richard Hamelin (INSERM, Paris, France) and LoVo by Professor Kevin Ryan (The Beatson Institute for Cancer Research, Glasgow, UK). HCT116 cells and isogenic derivatives lacking *BAK* and/or *BAX* were provided by Bert Vogelstein and Richard Youle (NIH, Bethesda, Maryland, USA).

Human melanoma cell lines A2058, CHL-1, HMCB, MeWo, PMWK, RPMI-7951, SK-MEL-2, SK-MEL-5, SK-MEL-28 and SK-MEL-31 were from ATCC. SK-MEL-30 cells were from DSMZ (Leibniz Institute DSMZ-German Collection of Microorganisms and Cell Cultures, Braunschweig, Germany). WM266-4 and WM852 cells were from ESTDAB (European Searchable Tumour Cell and Data Bank, Centre for Medical Research (ZMF), Tübingen, Germany). A375 cells were provided by Richard Marais (Cancer Research UK Manchester Institute, Manchester, UK). MEL-JUSO cells were provided by Professor Judith Johnson (Institute of Immunology, University of Munich, Munich, Germany). MEDI-MEL-07 and MEDI-MEL-10 primary patient-derived human melanoma cells were provided by AstraZeneca (Gaithersburg, Maryland, USA).

Human non-small cell lung cancer (NSCLC) cell lines A549, Calu-6, NCI-H520, NCI-H727, NCI-H838, NCI-H1299, NCI-H1437, NCI-H1793, NCI-H1869, NCI-H2122 and NCI-H2286 were from ATCC. HARA cells were from JCRB (Japanese Collection of Research Bioresources Cell Bank, National Institutes of Biomedical Innovation, NIBIO, Osaka, Japan) and HCC15 cells were from DSMZ. Human pancreatic adenocarcinoma cell lines AsPC-1, BxPC3, Capan-1, CFPAC-1, HPAC, HPAF-II, Hs 700T, MIA PaCa-2, Panc 02.03, Panc 03.27, Panc 04.03, Panc 08.13, Panc 10.05, PANC-1 and PSN1 were from ATCC. HuP-T4 and PSN1 cells were from ECACC, and YAPC cells were from DSMZ.

Cells were grown in Dulbecco’s modified Eagle’s medium (DMEM) (A375, A549, A2058, CO115, DLD-1, HCA7, HCT116, Hs 700T, LoVo, MEDI-MEL-07, MEDI-MEL-10, MIA PaCa-2, PANC-1), IMDM (CFPAC-1), Leibovitz’s L-15 (SW48, SW480, SW620, SW837, SW1116, SW1417), McCoy’s 5A (HT29), MEM alpha (Calu-6, HMCB, HPAF-II, HuP-T4, LS174T, MeWo, PMWK, RPMI-7951, SK-MEL-2, SK-MEL-5, SK-MEL-28, WM266-4, WM852) or RPMI 1640 (AsPC-1, BxPC3, COLO205, HARA, HCC15, HCT8, LS411N, NCI-H520, NCI-H727, NCI-H747, NCI-H838, NCI-H1299, NCI-H1437, NCI-H1793, NCI-H1869, NCI-H2122, NCI-H2286, PSN1, SK-MEL-30, YAPC) media supplemented with 10% (v/v) fetal bovine serum, penicillin (100 U mL^−1^), streptomycin (100 mg mL^−1^) and 2 mM glutamine. DMEM for MEDI-MEL-07 and MEDI-MEL-10 cells was additionally supplemented with 0.1 mM non-essential amino acids and 25 mM HEPES. Leibovitz’s L-15 media was additionally supplemented with 0.75 mg mL^−1^ sodium bicarbonate. Caco-2 cells were grown in MEM alpha, 20% (v/v) fetal bovine serum, penicillin (100 U mL^−1^), streptomycin (100 mg mL^−1^) and 2 mM glutamine. Capan-1 cells were grown in IMDM, 20% (v/v) fetal bovine serum, penicillin (100 U mL^−1^), streptomycin (100 mg mL^−1^) and 2 mM glutamine. HPAC cells were grown in 1:1 DMEM/Ham’s F12 medium mix, 0.002 mg mL^−1^ insulin (Sigma-Aldrich, Dorset, UK), 0.005 mg mL^−1^ transferrin (Sigma-Aldrich), 40 ng mL^−1^ hydrocortisone (Sigma-Aldrich), 10 ng mL^−1^ human recombinant epidermal growth factor (Sigma-Aldrich) and 5% fetal bovine serum, penicillin (100 U mL^−1^), streptomycin (100 mg mL^−1^) and 2 mM glutamine. Panc 02.03, Panc 03.27, Panc 04.03, Panc 08.13 and Panc 10.05 cells were grown in RPMI 1640 medium, 15% (v/v) fetal bovine serum, 1 U mL^−1^ insulin, penicillin (100 U mL^−1^), streptomycin (100 mg mL^−1^) and 2 mM glutamine. SK-MEL-31 cells were grown in MEM alpha medium, 15% (v/v) fetal bovine serum, penicillin (100 U mL^−1^), streptomycin (100 mg mL^−1^) and 2 mM glutamine. Cells were incubated in a humidified incubator at 37 °C and 5% (v/v) CO_2_. All cell lines were authenticated by short tandem repeat (STR) profiling and confirmed negative for mycoplasma prior to experiments commencing. Unless stated otherwise, the above reagents were from Gibco, Thermo Fisher Scientific (Paisley, UK).

A375 cells with acquired resistance to PLX4720 (A375 PLX-R), selumetinib (A375 Sel-R), or combined PLX4720 and selumetinib (A375 PLX/Sel-R1 and A375 PLX/Sel-R2) were provided by AstraZeneca. Cells were grown in RPMI 1640 media supplemented with 10% (v/v) fetal bovine serum, penicillin (100 U mL^−1^), streptomycin (100 mg mL^−1^), 2 mM glutamine and containing 0.66 µM PLX4720 (A375 PLX-R), 0.5 µM selumetinib (A375 Sel-R), 0.33 µM PLX4720 plus 0.25 µM selumetinib (A375 PLX/Sel-R1) or 0.66 µM PLX4720 plus 0.5 µM selumetinib (A375 PLX/Sel-R2).

### Mice

All animal studies were conducted in accordance with the National Institute of Health guidelines for animal research or with UK Home Office regulations, UK Animals (Scientific Procedures) Act of 1986 and approved by AstraZeneca’s Institutional Animal Care and Use Committee (IACUC). Animals were kept in a pathogen-free, AAALAC (Association for the Assessment and Accreditation of Laboratory Animal Care) or UK Home Office approved facilities on a 12 h light/dark cycle. The ambient temperature was 21 ± 2 °C and the humidity was 55 ± 10%. Mice were housed at 3–5 animals per cage with air exchange and cages were changed weekly. Food and water were provided ad libitum.

### Inhibitor compounds and cell culture treatments

AZD4320 (CAS: 1357576–48–7), AZD5991 (CAS: 2143010–83–5), AZ6197 (compound 35)^50^ and selumetinib (CAS: 606143–52–6) were supplied by AstraZeneca. A-1155463 (CAS: 1235034–55–5) and Q-VD-OPh (CAS: 1135695–98–5) were purchased from Adooq Bioscience (Irvine, California, USA). PLX4720 (CAS: 918505–84–7), SCH772984 (CAS: 942183–80–4), trametinib (CAS: 871700–17–3) and vemurafenib (CAS: 918504–65–1) were purchased from Selleck Chemicals (Houston, Texas, USA). Venetoclax (CAS: 1257044–40–8) was purchased from MedChemExpress (New Jersey, USA). All compounds were dissolved in dimethyl sulfoxide (DMSO) to a stock concentration of 10 mM.

For inhibitor compound treatments, cells were seeded into dishes or plates in their normal growth medium (containing the relevant inhibitors for cells with acquired resistance) and allowed to settle for 24 h. For experiments with resistant cells, all treatment groups were washed with complete media only and then treated with fresh media containing the indicated compounds or media containing vehicle-only. Vehicle DMSO concentrations were normalised so that they were equivalent for all treatments.

### Generation of PLX4720- and/or selumetinib-resistant cells

To generate A375 cells with acquired resistance to either PLX4720 or selumetinib, cells at 50% confluence were treated with a GI_50_ concentration of PLX4720 (66 nM) or selumetinib (50 nM). Upon confluence, the cells were split and the inhibitor concentration increased a 1 × GI_50_ step (i.e., from 66 to 132 nM PLX4720 and 50 to 100 nM selumetinib). This was continued for ~3–4 months until the cells grew normally in 10 × GI_50_ PLX4720 (0.66 µM; A375 PLX-R) or selumetinib (0.5 µM; A375 Sel-R).

To generate cell lines in parallel to those above with acquired resistance to both PLX4720 and selumetinib, A375 cells at 50% confluence were treated with a GI_50_ concentration of selumetinib (50 nM). Upon confluence the cells were split and additionally treated with 1 × GI_50_ PLX4720 (66 nM). Compound concentrations were alternately increased a 1 × GI_50_ step following splitting until after 4 months the cells grew normally in 5 × GI_50_ PLX4720/selumetinib (0.33 µM PLX4720 and 0.25 µM selumetinib; A375 PLX/Sel-R1). The same procedure was used to generate an independent cell line in parallel with resistance to 10 × GI_50_ PLX4720/selumetinib (0.66 µM PLX4720 and 0.5 µM selumetinib; A375 PLX/Sel-R2).

### Generation of vemurafenib-, selumetinib- and/or AZD5991-resistant cells

A375 or WM266-4 cells at 50% confluence and growing in 175 cm^3^ tissue culture flasks were treated with 2 µM (A375) or 1 µM (WM266-4) vemurafenib and/or 1 µM AZD5991 as indicated in the Figure legends. SK-MEL-30 cells at 50% confluence and growing in 175 cm^3^ tissue culture flasks were treated with 1 µM selumetinib and/or 1 µM AZD5991 as indicated in the Figure legends.

Cells were then either split as required or media changed twice a week until all cells grew at a stable rate for >4 weeks. Cumulative doublings tallies were calculated by tracking splitting ratios and using the formula: number of doublings = log_2_(splitting dilution factor).

### CRISPR-mediated gene editing

Guide RNAs (gRNAs) to *BCL2L11* (encoding BIM) and *BMF* were designed using the Zhang laboratory gRNA designing tool (http://crispr.mit.edu/) and cloned into a pSpCas9(BB)-2A-GFP genome editing vector, which was a gift from Feng Zhang (Addgene plasmid #48138). The gRNA sequences were *BIM* gRNA2 5′- caccGCAACCACTATCTCAGTGCAA-3′; 5′- aaacTTGCACTGAGATAGTGGTTGC-3′ and *BMF* gRNA1 5′-caccGAAGAGCTGAAGTCGGCTGA-3′; 5′-aacTCAGCCGACTTCAGCTCTTC-3′. A375 or SK-MEL-30 cells were transfected with the *BCL2L11* and/or *BMF* gRNA containing Cas9 plasmids either individually or in combination using jetPRIME (Polyplus Transfection, Illkirch, France). Transfection was monitored by green fluorescent protein (GFP) expression and single GFP-positive/4′,6-diamidino-2-phenylindole (DAPI)-negative cells were sorted (Supplementary Data 1) in to 96-well plates using a 100 µm nozzle on a BD FACSAria III cell sorter (BD Biosciences, Oxford, UK). Clones of interest were identified by western blot screening for absence of BIM and/or BMF as well as control clones (WT), which still fully expressed the proteins despite transfection with the gRNA containing Cas9 plasmids. Uncropped western blot images of these clones in Fig. 6e are shown in Supplementary Data 2.

To confirm the presence or absence of *BIM* and *BMF* mutations genomic DNA was extracted from A375 (control untransfected) and all WT, BIM and BMF single and double KO clones. Cells were lysed with Tail Lysis Buffer (50 mM Tris pH 8.0, 5 mM EDTA, 0.25% SDS, 200 mM NaCl) and 200 μg mL^−1^ Proteinase K added to the lysate prior to incubation at 55 °C overnight. An equivalent volume of phenol/chloroform/isoamyl alcohol 25:24:1 (v/v) saturated with 10 mM Tris pH 8.0, 1 mM EDTA (Sigma-Aldrich, Dorset, UK) was then added and tubes mixed by inversion. After centrifugation, the DNA-containing aqueous phase was collected and precipitated by adding 0.8 volumes of 100% isopropanol (Sigma-Aldrich, Dorset, UK). The precipitated DNA was pelleted by centrifugation, washed with 70% (v/v) ethanol and solubilised in nuclease-free water.

Genomic DNA flanking the CRISPR guide-binding site was amplified by PCR using OneTaq (NEB, Hitchin, UK) according to the manufacturer’s instructions and using the following primers: *BIM* 5′-CTAACCCCGGGAAGTCAGAG-3′; 5′-TTGACACATCCTCCATTCCC-3′ and *BMF* 5′-CGGCCTAGGTCAGAAAACGTG-3′; 5′-GCAGGTGGAAGTCAAGGAATC-3′. The products generated were then cloned into the TOPO-TA cloning vector (Thermo Fisher Scientific, Loughborough, UK) following the manufacturer’s instructions. The resulting constructs were used to transform chemically competent DH5α (NEB, Hitchin, UK), and 4–5 of the resulting clones derived from DNA for each cell line were sent for sequencing (Genewiz, Bishop’s Stortford, UK).

### Xenograft studies

Compounds were formulated as follows. Selumetinib or trametinib were dissolved in 0.5% (w/v) Methocel hydroxypropyl methylcellulose (HPMC; Colorcon, Pennsylvania, USA)/0.1% (v/v) Polysorbate 80 (VWR, Pennsylvania, USA). Vemurafenib was dissolved in a 50/30/20 mixture of PEG-400 (MilliporeSigma, Massachusetts, USA), Koliphor-ELP (Sigma-Aldrich, St. Louis, USA), and 60% Captisol (Cydex Pharamceuticals, Kansas, USA). Selumetinib, trametinib and vemurafenib were protected from light and stored at room temperature for up to 7 days. AZD5991 was dissolved in 30% (w/v) hydroxypropyl-β-cyclodextrin (HPBCD; CTD Inc, Florida, USA)/1 M Meglumine (Sigma-Aldrich, St. Louis, USA) and brought to pH 9.0. AZD5991 was prepared fresh each week. The ERK1/2 inhibitor AZ13776197 was formulated in a 10% (v/v) DMSO/36% (w/v) HPBCD (Kleptose HPB-LB parenteral grade, Roquette, Corby, UK) vehicle.

Xenografts of A375 cells were performed in female nude athymic (Ncr-*Foxn1*^*nu*^) mice (Taconic Biosciences, Rensselaer, New York, USA). Mice were implanted subcutaneously with 5 × 10^6^ A375 cells in a 50:50 mixture of phosphate buffered saline (PBS):Matrigel (BD Biosciences, San Jose, California, USA). Injection volume was 0.1 mL per mouse. Injections were performed on the right flank of the animal. Once palpable, tumours were measured twice weekly using calipers and animal body masses recorded. Twenty-one days post-implantation mice were randomised into treatment arms based on tumour size. The vehicle-only control group contained five animals. All other arms contained seven to eight animals. Selumetinib (25 mg kg^−1^) was dosed by oral gavage twice daily on an 8 h interval. AZD5991 (60 mg kg^−1^) was injected intravenously once weekly. AZD5991 treatment was started 1 day after selumetinib and given 4 h after the morning dose of the respective combination partner.

All tumour patient-derived xenograft (PDX) models used in this study came from the internal AstraZeneca PDX library and had the appropriate patient consent and Institutional Review Board (IRB) approval. MEDI-MEL-02 melanoma (BRAF^V600E^ mutant) was from a 50-year-old female and tumour tissue taken from the right upper arm. The passage used in this study was P5. MEDI-MEL-10 melanoma (BRAF^V600E^ mutant) was from a 72-year-old male and tumour tissue taken from a lung metastasis. The passage used in this study was P6. Neither patient had previously received BRAFi or MEKi. PDX tumours were initially propagated in seed female NOD SCID (NOD.CB17-*Prkdc*^*scid*^/NCrHsd) mice (Taconic Biosciences, Rensselaer, New York, USA), to generate sufficient tumour material to seed each efficacy study. Once tumours reached 0.8–1.2 cm^3^ the mice were humanely euthanised by CO_2_ asphyxiation. Tumours were isolated under sterile conditions, cut into ~2–3 mm^3^ pieces and implanted subcutaneously into the right flank of individual 10–12-week-old female NOD SCID mice using a 10-gauge trocar needle. Upon reaching an average size of ~0.15–2 cm^3^, mice were randomised (based on tumour volume and body weight) using the multi-task feature in Study Director software (Studylog Systems, California, USA) into the various treatment groups. The method uses a randomised block design to cluster mice into groups using both tumour volume and body mass. Mice were then dosed with vemurafenib (20 mg kg^−1^ by oral gavage twice daily on an 8 h interval), trametinib (1 mg kg^−1^ by oral gavage once daily) and/or AZD5991 (60 mg kg^−1^ intravenously three times per week on Monday, Wednesday and Friday). Tumour volumes were measured twice weekly using calipers and animal body mass and tumour condition also recorded twice weekly.

A375 Sel/PLX-R2 xenografts were performed in female athymic nude mice (Hsd:Athymic Nude-*Foxn1*^*nu*^; Envigo, Huntingdon, UK). Mice were implanted subcutaneously with 5 × 10^6^ A375 Sel/PLX-R2 cells on the left flank in a volume of 0.1 mL per mouse. On day 6 post-implant, mice were randomised into control and treatment groups of 10 mice with an average tumour volume of ~0.2 cm^3^ and dosing commenced the day after randomisation for a duration of 21 days. Mice received 50 mg kg^−1^ AZ13776197 administered once daily by oral gavage starting on day 1 post randomisation and 60 mg kg^−1^ AZD5991 was given once weekly intravenously 2 h after administration of AZ13776197 starting from day 4 post randomisation. Mice in control groups received a combination dose of both vehicles used. Tumour volumes were measured twice weekly using calipers and animal body mass and tumour condition also recorded twice weekly.

In all experiments tumour length (*L*) and width (*W*) were measured using calipers and tumour volume calculated using the formula for the volume of a prolate spheroid *V* = 4/3π(*L*/2)(*W*/2)².

### High-throughput drug combination assays and synergy scores

Cells were seeded in 384-well black wall imaging plates (ViewPlate-384, Perkin Elmer, Buckinghamshire, UK) and allowed to settle for 24 h. Cells were then treated with vemurafenib (top concentration 10 μM), trametinib (top concentration 0.3 μM), selumetinib (top concentration 10 μM) or SCH772984 (top concentration 3 μM) in combination with AZD5991 (top concentration 10 μM) or AZD4320 (top concentration 10 μM) in a half-log 8 × 8 concentration matrix. Drug dispensing was performed using a cell::explorer HCS robot (Perkin Elmer, Buckinghamshire, UK). Following 5 days treatment, cells were incubated with the DNA intercalating fluorescent dye Sytox Green (Thermo Fisher Scientific, Loughborough, UK) at 0.16 μM (diluted in TBS-EDTA buffer: 0.1 M Tris, 0.15 M NaCl, 5 mM EDTA, pH 7.0) for 1 h and dead cells in each well imaged following fluorescence stimulation at 488 nm using a Nikon Eclipse Ti-E microscope (Nikon, Nikon Instruments, Amsterdam, Netherlands). Cells were then permeabilised by addition of 0.04% (w/v) saponin/TBS-EDTA buffer for 4 h and total cells in each well imaged. Dead and total cells in images were counted for each well using NIS-Elements Advanced Research (Nikon, Nikon Instruments, Amsterdam, Netherlands) or Ilastik (http://ilastik.org/). Cell numbers per well were also determined at the point of treatment (day 0) using this methodology in parallel 384-well plates. The number of live cells for both day 0 and day 5 was calculated by subtracting the dead read from the total read. Data are shown using the NCI formulas [(*L* – *L*_0_)/(*C* – *L*_0_)] × 100 if *L* ≥ *L*_0_ and [(*L* – *L*_0_)/*L*_0_] × 100 if *L* < *L*_0_, where *L* represents the number of live cells in the presence of drug, *L*_0_ represents the number of live cells at time zero (day 0) and *C* represents the control (DMSO-only vehicle) live cell number. This gives a 100%, 0, −100% scale of live cell number, in which 100% is the control (DMSO-only vehicle) live cell number, 0 is the day 0 live cell number and negative percentages indicate cell death with −100% meaning no live cells were present. Three independent experiments were performed for each drug combination and cell line. Mean data are presented in heat maps generated using GraphPad Prism 7 (GraphPad Software, La Jolla, California, USA). Synergy scores were generated using the Loewe additivity model and calculated in the combination extension of Genedata Screener 12 (Genedata, Basel, Switzerland). Unless otherwise indicated, reagents were from Sigma-Aldrich, Dorset, UK.

### Preparation of cell lysates for sodium dodecyl sulphate–polyacrylamidegel electrophoresis (SDS-PAGE) and western blotting

Culture medium from cells growing on dishes was either removed or non-adherent cells/cellular material recovered from the medium by centrifugation (400 × *g* at room temperature (RT) for 3 min (min)). Cells were washed with PBS and then lysed for 5 min with ice-cold RIPA lysis buffer (20 mM Tris-HCl (pH 7.4), 137 mM NaCl, 1 mM EGTA, 1% (v/v) Triton X-100, 0.1% (w/v) SDS (Bio-Rad, Watford, UK), 1% (w/v) sodium deoxycholate, 10% (v/v) glycerol, 1.5 mM MgCl_2_, 50 mM NaF, 1 mM Na_3_VO_4_, 5 μg mL^−1^ aprotinin, 10 μg mL^−1^ leupeptin, 1 mM PMSF, 0.025 U mL^−1^ Benzonase). Lysates were detached and collected using a cell scraper and transferred to pre-chilled tubes. Protein concentration was determined by BCA protein assay (Pierce BCA Protein Assay Kit, Thermo Fisher Scientific, Loughborough, UK) and absorbance measured at 562 nm using a PHERAstar FS plate reader (BMG Labtech, Aylesbury, UK). Samples were prepared for SDS-PAGE by boiling for 5 min in 1 × Laemmli sample buffer (50 mM Tris-HCl (pH 6.8), 2% (w/v) SDS (Bio-Rad, Watford, UK), 10% (v/v) glycerol, 1% (v/v) β-mercaptoethanol, 0.01% (w/v) bromophenol blue). Unless otherwise indicated, reagents were from Sigma-Aldrich, Dorset, UK.

### Preparation of tumour tissue homogenates

Colorectal and melanoma PDX tumour tissue was from the internal AstraZeneca PDX library. Snap frozen tumour fragments were homogenised in ice-cold RIPA lysis buffer (20 mM Tris-HCl (pH 7.4), 137 mM NaCl, 1 mM EGTA, 1% (v/v) Triton X-100, 0.1% (w/v) SDS (Bio-Rad, Watford, UK), 1% (w/v) sodium deoxycholate, 10% (v/v) glycerol, 1.5 mM MgCl_2_, 50 mM NaF, 1 mM Na_3_VO_4_, 5 μg mL^−1^ aprotinin, 10 μg mL^−1^ leupeptin, 1 mM PMSF, 0.025 U mL^−1^ Benzonase) using gentleMACS M tubes and a gentleMACS Dissociator on setting Protein_0.1.01 (Miltenyi Biotec, Surrey, UK). Lysates were collected by centrifugation (400 × *g*, 4 °C, 1 min) and protein concentration determined by BCA protein assay (Pierce BCA Protein Assay Kit, Thermo Fisher Scientific, Loughborough, UK) and absorbance measured at 562 nm using a PHERAstar FS plate reader (BMG Labtech, Aylesbury, UK). Samples were prepared for SDS-PAGE by boiling for 5 min in 1 × Laemmli sample buffer (50 mM Tris-HCl (pH 6.8), 2% (w/v) SDS (Bio-Rad, Watford, UK), 10% (v/v) glycerol, 1% (v/v) β-mercaptoethanol, 0.01% (w/v) bromophenol blue). Unless otherwise indicated, reagents were from Sigma-Aldrich, Dorset, UK.

### SDS-PAGE and western blotting

Lysates were separated by SDS-PAGE (Mighty small II gel apparatus, Hoefer, Massachusetts, USA). Polyacrylamide gels consisted of a resolving phase of 8–16% (w/v) acrylamide (37.5:1 acrylamide:bisacrylamide, 2.7% crosslinker; Bio-Rad, Watford, UK), 0.375 M Tris-HCl (pH 8.8), 0.2% (w/v) SDS (Bio-Rad, Watford, UK), 0.1% (w/v) ammonium persulfate, 0.1% TEMED (Bio-Rad, Watford, UK) and a stacking phase of 4.5% (w/v) acrylamide (37.5:1 acrylamide:bisacrylamide, 2.7% crosslinker), 0.125 M Tris-HCl (pH 6.8), 0.2% (w/v) SDS, 0.1% ammonium persulfate, 0.125% TEMED. Gels were run using running buffer (0.2 M glycine, 25 mM Tris, 0.1% (w/v) SDS) and a current of 15 mA per gel for 3–4 h. Gels were then blotted by wet transfer (Bio-Rad, Watford, UK) to methanol activated PVDF (Immobilon-P Membrane, Merck Millipore, Watford, UK) using transfer buffer (0.2 M glycine, 25 mM Tris, 20% (v/v) methanol) and a current of 300 mA for 90 min. Membranes were blocked in 5% milk/TBST (5% (w/v) non-fat powdered milk (Marvel, Premier Foods, St Albans, UK), 10 mM Tris-HCl (pH 7.6), 150 mM NaCl, 0.1% (v/v) Tween-20) for 1 h at room temperature. For quantification of total protein using REVERT and processing using the Odyssey system, membranes were incubated for 5 min with REVERT solution and washed following the manufacturer’s instructions (LI-COR, Cambridge, UK), and then blocked in 5% milk/PBS for 1 h. Membranes were incubated with primary antibodies against ARAF, BAD, BCL-X_L_, BID, BIM, ERK1/2, MCL1, MEK1/2, PARP, phospho-ERK1/2 (T185 Y187/T202 Y204), phospho-MEK1/2 (S218 S222/S222 S226), phospho-RSK (T359), PUMA, RSK (Cell Signalling Technology, NEB, Hitchin, UK), BAK, BAX, BCL2, BRAF, CRAF, MCL1, NRAS (Santa Cruz Biotechnology, Dallas, USA), BAX (BD Biosciences, Oxford, UK), BIM, NOXA (Merck Millipore, Watford, UK), BMF (Enzo Life Sciences, Exeter, UK), GAPDH (Abcam, Cambridge, UK), KRAS (Proteintech, Manchester, UK), β-actin and α-tubulin (Sigma-Aldrich, Dorset, UK) diluted as recommended in 5% milk/TBST or 5% BSA/TBST overnight at 4 °C with agitation. Membranes were then washed in TBST for 4 × 10 min, and incubated with horseradish peroxidase-conjugated secondary antibodies (Bio-Rad, Watford, UK) diluted 1:3000 in 5% milk/TBST, or fluorescently labelled secondary antibodies for quantification diluted 1:15,000 (Cell Signalling Technology, NEB, Hitchin, UK), for 1 h at room temperature. Membranes were again washed for 4 × 10 min in TBST. Detection was performed using Amersham ECL Western Blotting Detection Reagent (GE Healthcare Life Sciences, Buckinghamshire, UK), Clarity Western ECL Substrate (Bio-Rad, Watford, UK) or Immobilon Western Chemiluminescent HRP Substrate (Merck Millipore, Watford, UK), X-ray film and Compact X4 film developer (Xograph, Gloucestershire, UK). Quantification of fluorescently labelled membranes was performed using the Odyssey infrared imaging system (LI-COR, Cambridge, UK). Unless otherwise indicated, reagents were from Sigma-Aldrich, Dorset, UK. Uncropped western blot images of Fig. 6a, e are shown in Supplementary Data 2 and 3.

### Absolute quantification of MCL1 and BCL-X_L_

Cells seeded and cultured for 24 h were lysed in RIPA lysis buffer and protein concentrations determined as above. Colorectal and melanoma PDX tumour tissue was homogenised and protein concentrations determined as described above. Recombinant protein standards for MCL1 (Cloud-Clone Corp, Texas, USA) and BCL-X_L_ (R&D Systems, Abingdon, Oxfordshire, UK) were diluted in RIPA with 1 × Laemmli sample buffer to a concentration range of 2.5–100 nM. SDS-PAGE was then performed with 5–6 standards of 0.0375 pmol to 1.5 pmol MCL1 or BCL-X_L_ protein to determine the levels of MCL1 and BCL-X_L_ in 15 to 20 µg of total cellular protein by western blotting with anti-MCL1 antibody (Santa Cruz Biotechnology, Dallas, USA; sc-819) and anti-BCL-X_L_ antibody (Cell Signalling Technology, NEB, Hitchin, UK; 2762) and using anti-rabbit fluorescently labelled secondary antibodies (Cell Signalling Technology, NEB, Hitchin, UK) diluted 1:15,000 and infrared imaging for quantification (LI-COR, Cambridge, UK).

### Immunoprecipitation

Cells were lysed in ice-cold TG lysis buffer (20 mM Tris-HCl (pH 7.4), 137 mM NaCl, 1 mM EGTA, 1% (v/v) Triton X-100, 10% (v/v) glycerol, 1.5 mM MgCl_2_, 50 mM NaF, 1 mM Na_3_VO_4_, 5 μg mL^−1^ aprotinin, 10 μg mL^−1^ leupeptin, 1 mM PMSF), and incubated with 50 µL protein A-sepharose beads (Sigma-Aldrich, Dorset, UK; bead slurry of 10 g resuspended in 40 mL PBS) per mL of lysate for 30 min at 4 °C to preclear. At the same time 200 µL protein A-sepharose beads were incubated in 750 µL TG lysis buffer with anti-BCL-X_L_ antibody (Cell Signalling Technology; 2762 or 2764) diluted 1:50, anti-MCL1 antibody diluted 1:25 (Cell Signalling Technology; 94296) or anti-MCL1 antibody (Santa Cruz Biotechnology; sc-819) diluted 1:10 and incubated at 4 °C with rotation for 1 h. Following preclearing, lysates were centrifuged at 13,000 × *g* at 4 °C for 10 min and supernatants transferred to fresh pre-chilled tubes to remove protein A-sepharose beads and insoluble material. Lysates were then subjected to protein assay using Bradford reagent in which 5 µL cleared lysate was mixed with 795 µL ultra-pure water and 200 µL Bradford reagent (Bio-Rad Laboratories, Watford, UK), and absorbance measured at 595 nm using a PHERAstar FS plate reader (BMG Labtech, Aylesbury, UK). Seven hundred and fifty microlitres of normalised lysate were then added to washed protein A-sepharose beads bound to antibody and incubated for 4 h at 4 °C with rotation. Residual lysate was retained as input samples and prepared for SDS-PAGE by boiling for 5 min in 1 × Laemmli sample buffer. Immunoprecipitate supernatants were retained for unbound supernatant fraction analysis and boiled for 5 min in 1 × Laemmli sample buffer, while protein A-sepharose beads harbouring immunoprecipitate were washed four times in TG lysis buffer, resuspended in 1 × Laemmli sample buffer and boiled for 5 min. Lysates were then subjected to SDS-PAGE and western blotting. For western blotting of immunoprecipitates, the majority of primary antibodies were detected using protein G–horseradish peroxidase (Bio-Rad Laboratories, Watford, UK), to avoid heavy and light chain interference from the antibodies used in the immunoprecipitation reactions. Unless otherwise indicated, reagents were from Sigma-Aldrich, Dorset, UK.

### Colony formation assays

A375 cells were seeded in 12-well plates at 150 cells per well, allowed to settle for 24 h and treated as indicated in the Figure legends. Seventy-two hours later, cells were washed and the medium replaced with fresh medium only without compounds. Seven days after initial treatment colonies were fixed with 3:1 (v/v) methanol/acetic acid, washed in water, stained with 0.1% (w/v) crystal violet/PBS (Sigma-Aldrich, Dorset, UK) and counted using Adobe Photoshop (Adobe Systems Europe Ltd, Maidenhead, UK). Relative cell number was then assessed by solubilising cells in 10% (v/v) acetic acid and measuring absorbance at 590 nm.

### Cell cycle analysis by flow cytometry

Following treatment as indicated in the Figure legends, culture medium from cells growing on plates was collected, adherent cells trypsinised and cells and media then recombined. Cells were pelleted by centrifugation (400 × *g*, RT, 5 min) and resuspended in 0.2 mL PBS. Cells were fixed in 70% (v/v) ethanol/PBS at 4 °C for at least 30 min. Samples were then centrifuged (400 × *g*, 4 °C, 5 min), washed with PBS, and resuspended in 0.25 mL PBS containing 25 μg RNase A (Sigma-Aldrich, Dorset, UK) and 12.5 μg of the DNA-intercalating agent propidium iodide (PI; Sigma-Aldrich, Dorset, UK). Following 30 min incubation at 37 °C, cell cycle profiles were acquired with an LSR II flow cytometer (BD Biosciences, Oxford, UK) to measure binding of PI to DNA, and counting 10,000 cells per sample. Data was analysed using FlowJo (FlowJo LLC, Oregon, USA) software package and sub-G1, G1, S and G2-M cell cycle phases gated using the strategy shown in Supplementary Data 4.

### Annexin V-DAPI staining and flow cytometry

Following treatment as indicated in the Figure legends, culture medium from cells growing on dishes was collected, adherent cells trypsinised and cells and media then recombined. Cells were pelleted by centrifugation (400 × *g*, RT, 5 min), resuspended in 1 mL PBS and then centrifuged again before being washed further in 1 mL annexin V binding buffer (10 mM HEPES/NaOH (pH 7.4), 140 mM NaCl, 2.5 mM CaCl_2_). Cells were then resuspended in 0.2 mL annexin V binding buffer containing 1 µg mL^−1^ DAPI (Sigma-Aldrich, Dorset, UK) and 0.1 µg mL^−1^ annexin V-FITC (BioLegend, London, UK), and incubated for 10 min. Annexin V/DAPI staining was then assessed using an LSR II flow cytometer (BD Biosciences, Oxford, UK) and counting 10,000 cells per sample. Data was analysed using FlowJo (FlowJo LLC, Oregon, USA) software package to quantify annexin V and/or DAPI-positive cells using the gating strategy shown in Supplementary Data 5.

### BAX activation assay

Following treatment as indicated in the Figure legends, culture medium from cells growing on plates was collected, adherent cells trypsinised and cells and media then recombined. Cells were pelleted by centrifugation (400 × *g*, 4 °C, 5 min) and then fixed by resuspending in 1% (w/v) formaldehyde/PBS (Sigma-Aldrich, Dorset, UK) for 10 min at room temperature. Cells were then centrifuged (400 × *g*, 4 °C, 5 min), formaldehyde removed, and washed twice with PBS. Cells were permeabilised in 0.1% (w/v) saponin/PBS (Sigma-Aldrich, Dorset, UK) for 15 min at room temperature, washed once in PBS and then incubated with 10 μg mL^−1^ anti-BAX antibody (clone 6A7 specific for active BAX, BD Biosciences, Oxford, UK) diluted in PBS for 30 min on ice. Control samples incubated in PBS only (no primary anti-BAX antibody) were also included. Cells were washed three times in PBS and then incubated with 10 μg mL^−1^ anti-mouse fluorochrome-conjugated secondary antibody (anti-mouse Alexa Fluor 488, Thermo Fisher Scientific, Loughborough, UK) diluted in PBS for 30 min on ice. Following three washes in PBS, cells were resuspended in 200 μL PBS and BAX activity assessed using an LSR II flow cytometer (BD Biosciences, Oxford, UK) and counting 10,000 cells per sample. Data was analysed using FlowJo (FlowJo LLC, Oregon, USA) software package to quantify cells positive for active BAX under each condition using the gating strategy shown in Supplementary Data 6.

### Caspase activation assay

Cells were treated in 96-well plates as indicated in the Figure legends and caspase activation determined using Caspase-Glo 3/7 Assay (Promega, Southampton, UK) following the manufacturer’s instructions.

### DNA sequencing analysis

Cells were cultured in their normal growth medium containing PLX4720 and/or selumetinib as indicated in the Figure legends. Cell pellets were prepared, and DNA extracted using an AllPrep DNA/RNA/miRNA Universal Kit according to the manufacturer’s instructions, with an initial QIAShredder step (Qiagen, Manchester, UK). Two-hundred and twenty-five nanograms of purified genomic DNA was used for Next-Generation Sequencing (NGS) library construction. Sequencing libraries with 241 genes were generated using the HaloPlex Target Enrichment System for Illumina Sequencing Version D.3 (Agilent Technologies, Stockport, UK), following the manufacturer’s protocol. All libraries were visualised on the Agilent TapeStation 2200 prior to normalisation and sequencing. Libraries were sequenced on the Illumina HiSeq 2500 System using TruSeq SBS (sequencing by synthesis) reagents (Illumina, Eindhoven, Netherlands).

Raw-sequencing reads were mapped to the human reference genome hg19 using BWA-MEM aligner algorithm^57^. The VarDict pairwise algorithm^58^ was used to identify the significance of variant changes (*P*-value and odds ratio from Fisher’s exact test of allele frequency and depth) in resistant vs. parental cells. VarDict default filters applied for variant quality, where a variant needed to pass in either the parent or the resistant cell line. Significantly different variants were prioritised by removing variants with: *P*-value > 0.05 or odds ratio <1.5 and >0.66 for comparison between parental and resistant samples; AF difference in both directions <0.1; variant depth in both samples <100; PASS flag in the VarDict output not equal to TRUE or MAXRATE; silent mutations; SNPs; regions downstream or upstream of genes, intergenic regions, introns, 3′- and 5′-UTRs.

The Seq2C algorithm (https://github.com/AstraZeneca-NGS/Seq2C) was used to quantify gene level copy number changes. Depth of coverage was normalised to remove assay variability (position-specific variation in depth). Copy number is stated as a log_2_ difference in normalised depth to each of: all samples in this data set (and all measured genes); all genes in the specific sample; the gene of interest in the non-resistant sample. Copy events with log_2_ ratio change >3 were prioritised, and assessed for supporting SNP-specific changes in variant depth looking for: multiple SNPs to the gene showing coordinated grouping across samples; changes in depth of some SNPs supporting the copy number change direction, while others remain constant.

DNA sequencing data are available in the National Centre for Biotechnology Information (NCBI) Sequence Read Archive (https://www.ncbi.nlm.nih.gov/sra) under the project number PRJNA531927 [https://www.ncbi.nlm.nih.gov/bioproject/PRJNA531927].

### Statistical analyses

Results, unless otherwise indicated, are presented as mean ± standard deviation (SD) and originate from at least three independent experiments. Statistical significance was determined by two-tailed paired or unpaired *t*-test, two-tailed Mann–Whitney test, one-way ANOVA, Kruskal–Wallis test, or two-way ANOVA using GraphPad Prism 6 or 7. Multiple comparisons tests and adjustments were applied as indicated. Significance values were set at *P* > 0.05 (ns), *P* ≤ 0.05 (*), *P* ≤ 0.01 (**), *P* ≤ 0.001 (***) or *P* ≤ 0.0001 (****). Where bar charts are used, the individual data points have been overlaid, except in occasional cases where this detrimentally affects the clarity of the data.

## Supplementary information

Supplementary Information

Description of Additional Supplementary Files

Supplementary Data 1

Supplementary Data 2

Supplementary Data 3

Supplementary Data 4

Supplementary Data 5

Supplementary Data 6

## Data Availability

The DNA sequencing data have been deposited in the NCBI Sequence Read Archive (https://www.ncbi.nlm.nih.gov/sra) under the project number PRJNA531927 [https://www.ncbi.nlm.nih.gov/bioproject/PRJNA531927]. All the other data supporting the findings of this study are available within the article and Supplementary Information files and from the corresponding author upon reasonable request. A reporting summary for this article is available in the Supplementary Information.
