# Supplementary material for: Targeting melanoma’s MCL1 bias unleashes the apoptotic potential of BRAF and ERK1/2 pathway inhibitors
Source: Nat Commun. 2019 Nov 14;10:5167. doi: 10.1038/s41467-019-12409-w (PMC6856071; doi:10.1038/s41467-019-12409-w)
Supplement: Supplementary file 1 — Supplementary Information [file 41467_2019_12409_MOESM1_ESM.pdf]

Targeting melanoma's MCL1 bias unleashes the apoptotic potential of BRAF and ERK1/2 pathway inhibitors

Sale, M. J. et al.

Supplementary Information

Supplementary Information contains 14 Supplementary Figures, three Supplementary Tables and Supplementary References.

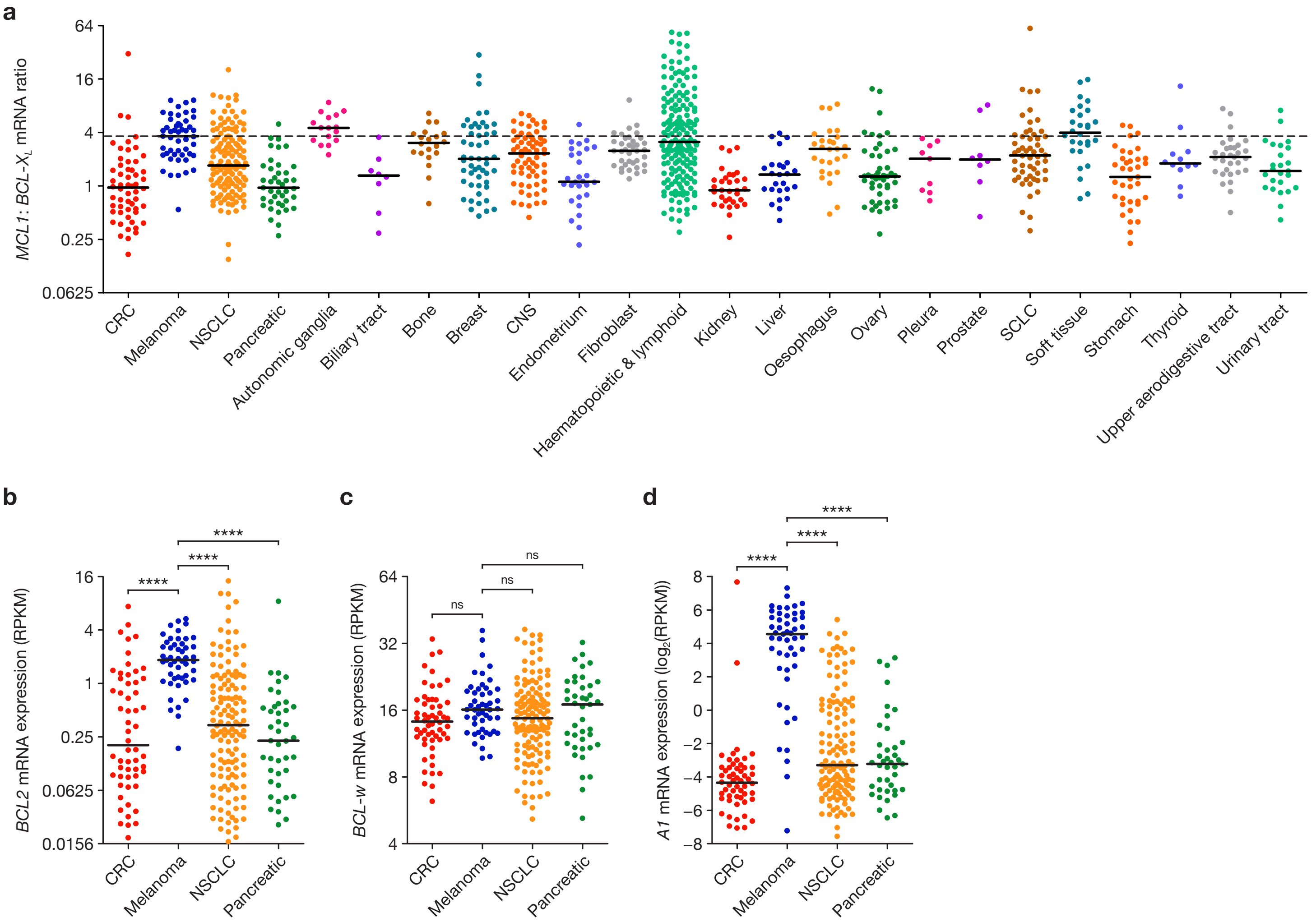

Supplementary Fig. 1 Low *BCL-X$_L$* mRNA expression in melanoma biases the pro-survival mRNA pool towards *MCL1*. **a** *MCL1*:*BCL-X$_L$* mRNA expression ratio obtained from the Cancer Cell Line Encyclopedia RNA-sequencing data for the indicated tumour lineages. CRC, colorectal cancer; NSCLC, non-small cell lung cancer; CNS, central nervous system; SCLC, small cell lung cancer. **b**-**d** RNA-sequencing mRNA expression data obtained from the Cancer Cell Line Encyclopedia for *BCL2* (**b**) *BCL-w* (*BCL2L2*) (**c**) and *A1* (*BCL2A1*) (**d**) in CRC, melanoma, NSCLC and pancreatic tumour cells measured in reads per kilobase million (RPKM). **a**-**d** Data are presented on a $\log_2$ scale as either antilog (**a**, **b**, **c**) or $\log_2$ (**d**) values, and black lines indicate median values. $P \leq 0.0001$ (****) or ns (not significant) as determined by Kruskal-Wallis and Dunn's multiple comparisons tests.

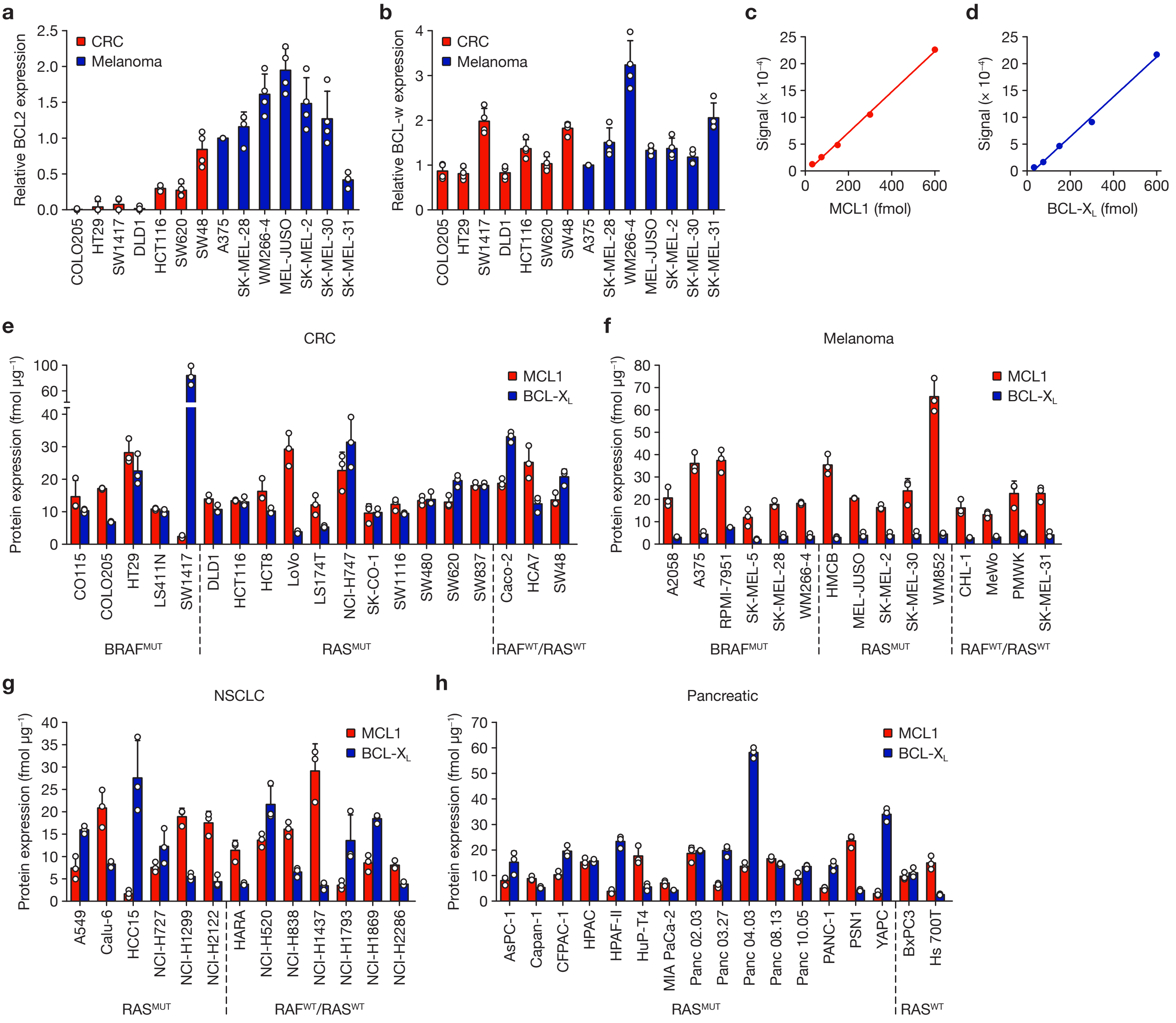

Supplementary Fig. 2 Low BCL-X_L protein expression in melanoma biases the pro-survival protein pool towards MCL1. **a**, **b** Seven colorectal cancer (CRC; left) and seven melanoma (right) cell lines were cultured for 24 hours before lysis and western blotting with the indicated antibodies. Quantification of BCL2 (**a**) and BCL-w (**b**) levels relative to A375 cells was performed by quantitative western blotting with fluorescently labelled secondary antibodies. Results are mean ± SD from three independent experiments. **c**, **d** 32.5-600 fmol of recombinant human MCL1 (**c**) or BCL-X_L (**d**) was subjected to SDS-PAGE and quantitative western blotting with fluorescently labelled secondary antibodies. Representative standard curves used for absolute quantification of MCL1 and BCL-X_L are shown. **e**-**h** MCL1 and BCL-X_L expression were quantified absolutely in the CRC (**e**), melanoma (**f**), non-small cell lung cancer (NSCLC; **g**) and pancreatic (**h**) tumour cell lines indicated using recombinant protein standards and quantitative western blotting with fluorescently labelled secondary antibodies. Results are mean ± SD from three independent experiments expressed in fmol per µg of total cellular protein (fmol µg^{-1}).

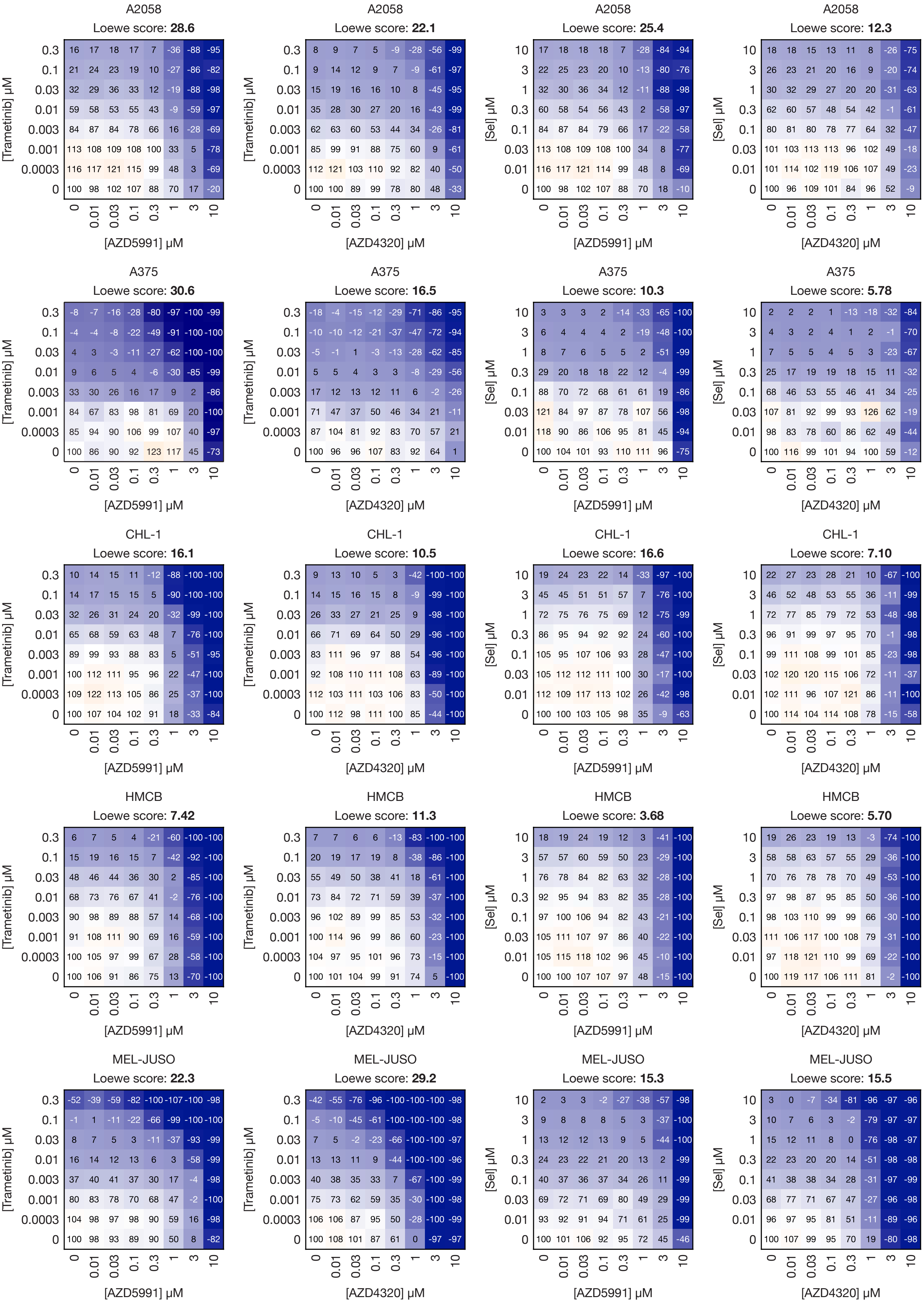

Supplementary Fig. 3 MEK1/2 inhibitors synergise with the MCL1 antagonist AZD5991 or BCL2/BCL-w/BCL-X_L antagonist AZD4320 to kill melanoma tumour cells. Melanoma cell lines were treated with the indicated concentrations of trametinib or selumetinib in combination with AZD5991 or AZD4320 for 5 days. The number of viable cells was determined at the point of treatment (day 0) and at the end of the experiment using Sytox Green. 100% represents the number of viable cells in the control wells at day 5, 0% is equivalent to the number of viable cells on day 0 and −100% indicates that no viable cells remained on day 5.

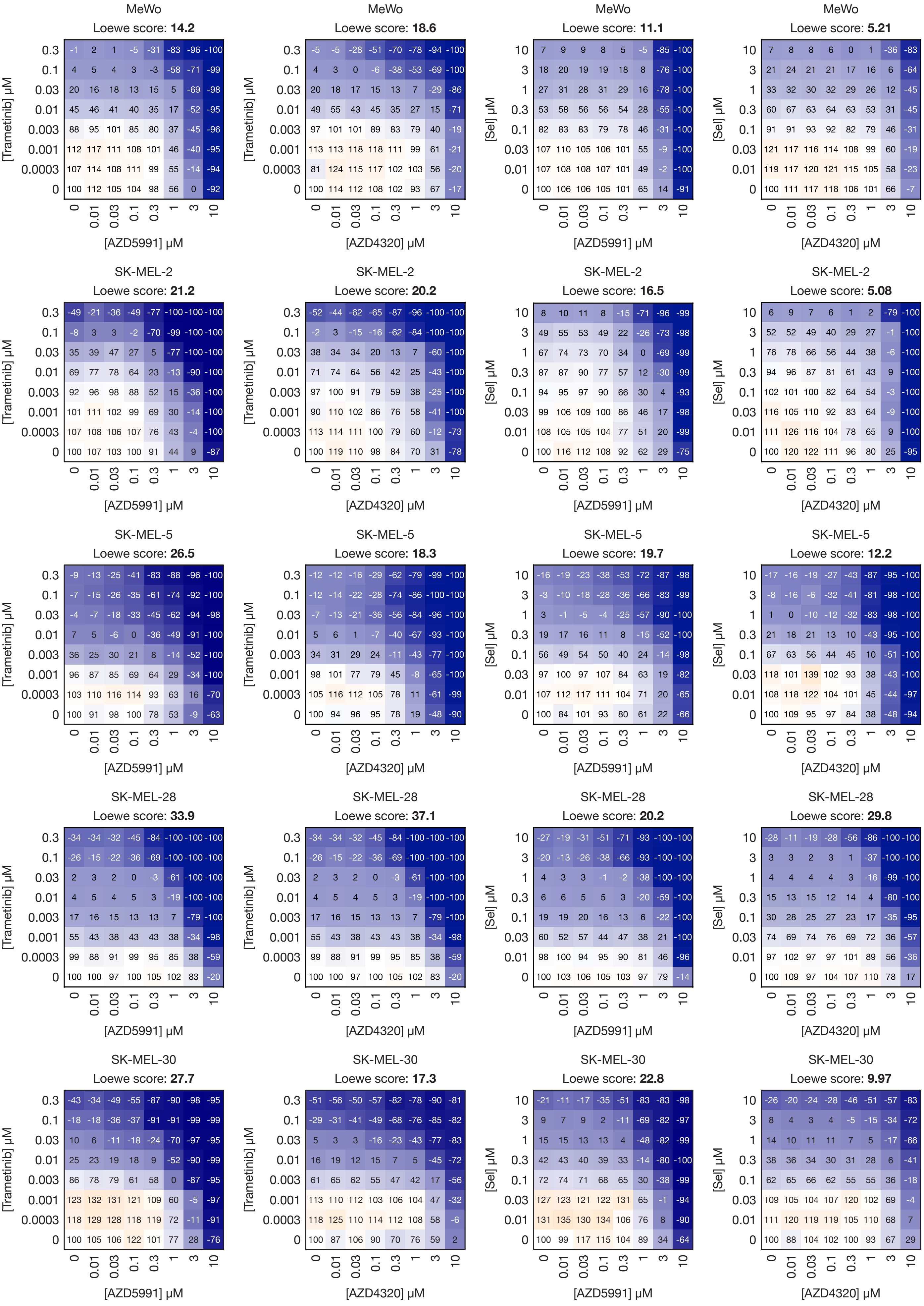

Supplementary Fig. 4 MEK1/2 inhibitors synergise with the MCL1 antagonist AZD5991 or BCL2/BCL-w/BCL-X_L antagonist AZD4320 to kill melanoma tumour cells. Melanoma cell lines were treated with the indicated concentrations of trametinib or selumetinib in combination with AZD5991 or AZD4320 for 5 days. The number of viable cells was determined at the point of treatment (day 0) and at the end of the experiment using Sytox Green. 100% represents the number of viable cells in the control wells at day 5, 0% is equivalent to the number of viable cells on day 0 and −100% indicates that no viable cells remained on day 5.

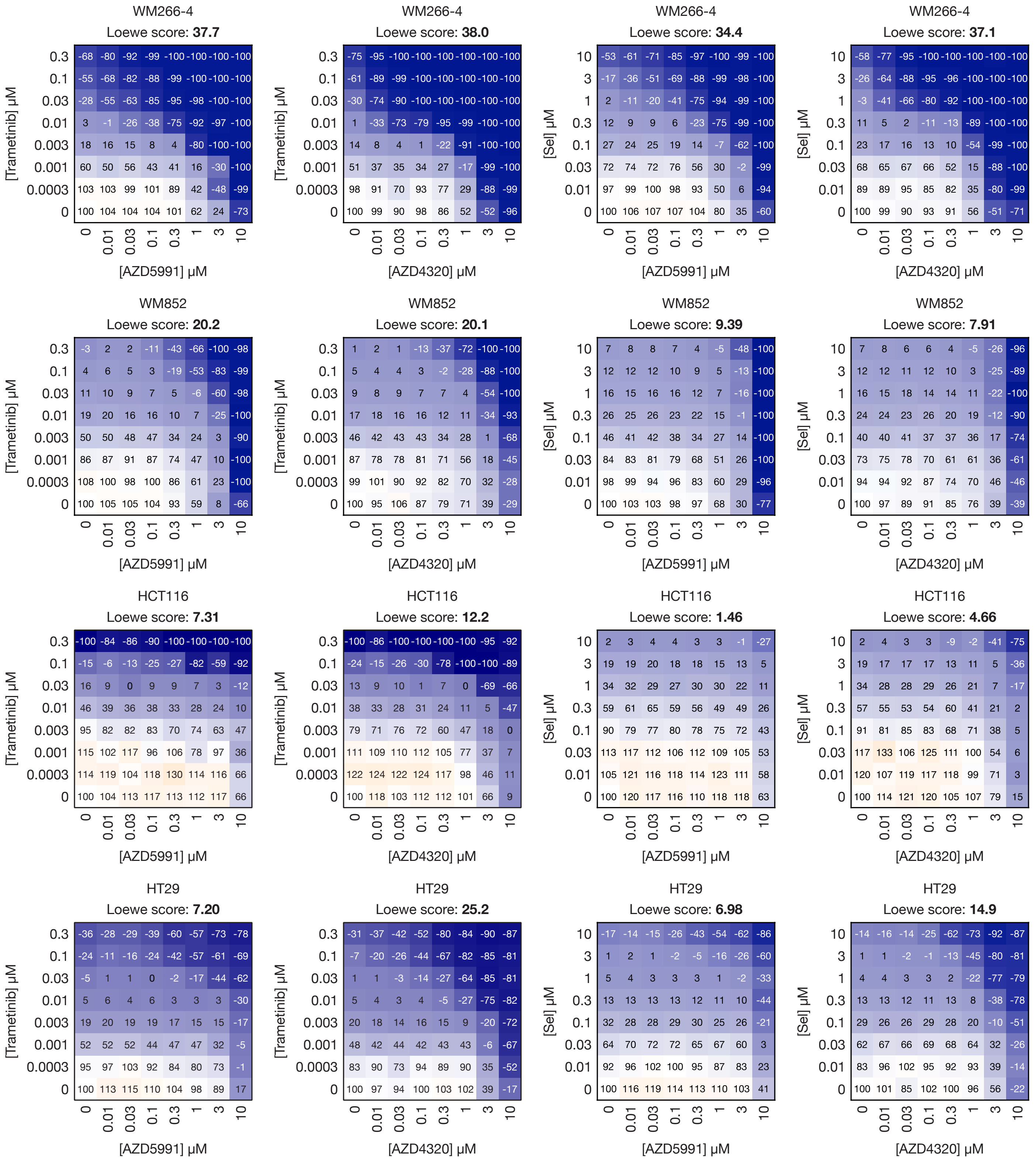

Supplementary Fig. 5 MEK1/2 inhibitors synergise with the MCL1 antagonist AZD5991 or BCL2/BCL-w/BCL-X_L antagonist AZD4320 to kill melanoma and colorectal cancer (CRC) tumour cells. Melanoma and CRC cell lines were treated with the indicated concentrations of trametinib or selumetinib in combination with AZD5991 or AZD4320 for 5 days. The number of viable cells was determined at the point of treatment (day 0) and at the end of the experiment using Sytox Green. 100% represents the number of viable cells in the control wells at day 5, 0% is equivalent to the number of viable cells on day 0 and –100% indicates that no viable cells remained on day 5.

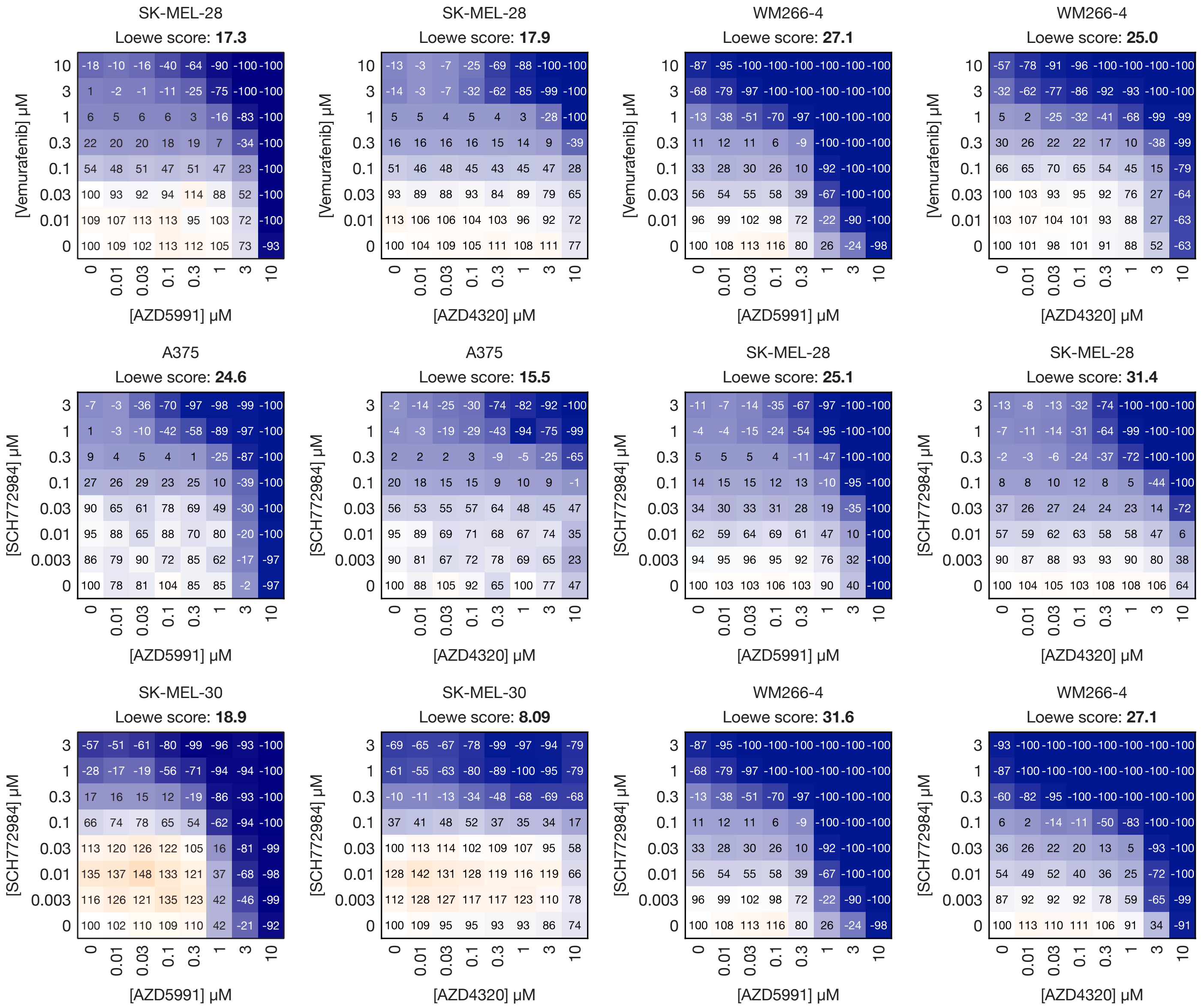

Supplementary Fig. 6 The BRAFi vemurafenib or the ERKi SCH772984 synergises with the MCL1 antagonist AZD5991 or BCL2/BCL-w/BCL-X_L antagonist AZD4320 to kill melanoma tumour cells. Melanoma cell lines were treated with the indicated concentrations of trametinib or selumetinib in combination with AZD5991 or AZD4320 for 5 days. The number of viable cells was determined at the point of treatment (day 0) and at the end of the experiment using Sytox Green. 100% represents the number of viable cells in the control wells at day 5, 0% is equivalent to the number of viable cells on day 0 and –100% indicates that no viable cells remained on day 5.

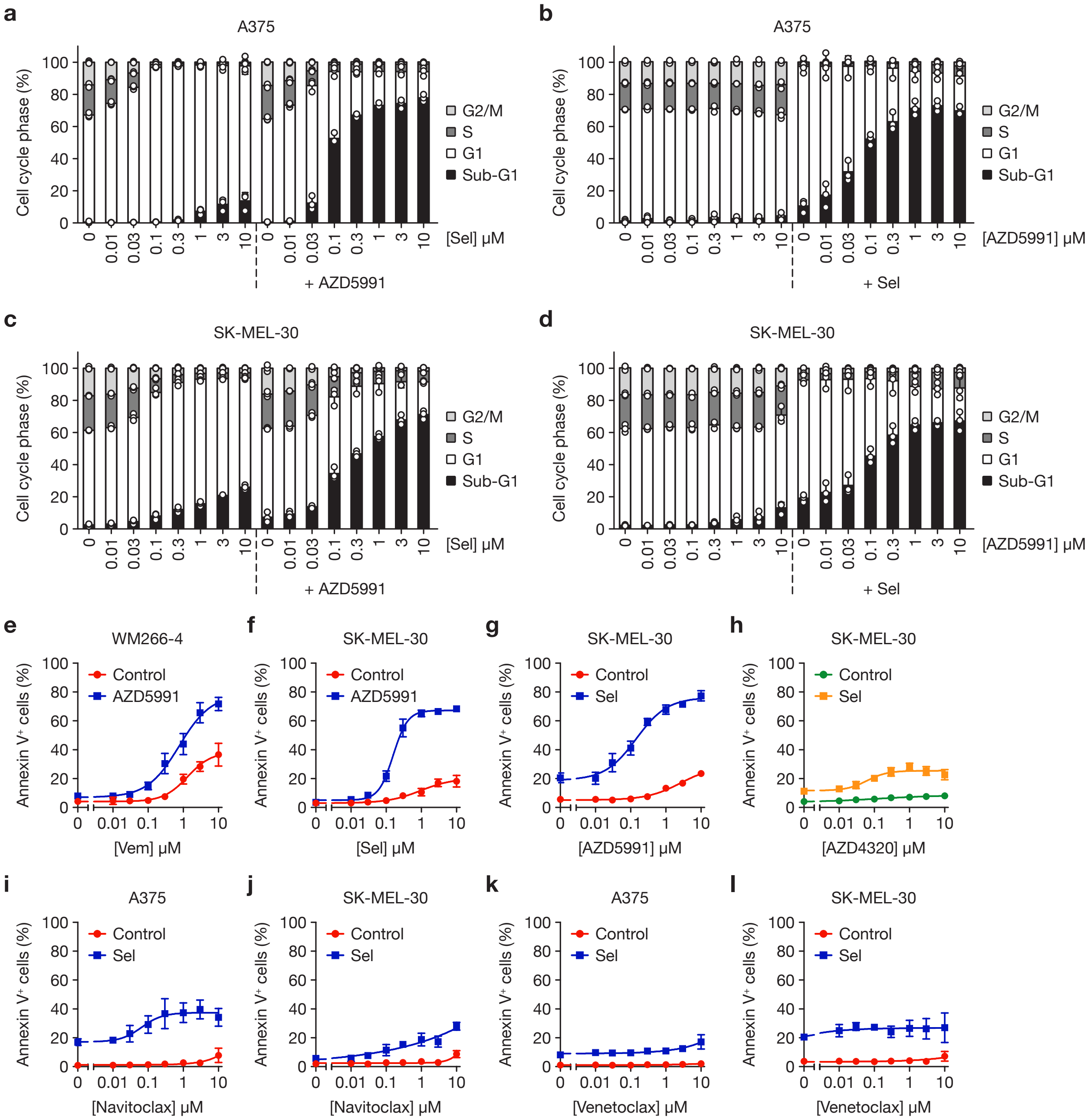

Supplementary Fig. 7 ERK1/2 pathway inhibitors combine with the MCL1 antagonist AZD5991 to selectively kill melanoma tumour cells. **a** A375 cells were treated with the indicated concentrations of selumetinib (Sel) with or without 1 µM AZD5991 for 48 hours. **b** A375 cells were treated with the indicated concentrations of AZD5991 with or without 1 µM selumetinib (Sel) for 48 hours. **c** SK-MEL-30 cells were treated with the indicated concentrations of selumetinib (Sel) with or without 1 µM AZD5991 for 48 hours. **d** SK-MEL-30 cells were treated with the indicated concentrations of AZD5991 with or without 1 µM selumetinib (Sel) for 48 hours. **a**-**d** Cell cycle profile was determined by propidium iodide staining and flow cytometry. **e** WM266-4 cells were treated with the indicated concentrations of vemurafenib (Vem) with or without 1 µM AZD5991 for 48 hours. **f** SK-MEL-30 cells were treated with the indicated concentrations of selumetinib (Sel) with or without 1 µM AZD5991 for 48 hours. **g**, **h** SK-MEL-30 cells were treated with the indicated concentrations of AZD5991 (**g**) or AZD4320 (**h**) with or without 1 µM selumetinib (Sel) for 48 hours. **i**, **j** A375 (**i**) and SK-MEL-30 (**j**) cells were treated with the indicated concentrations of navitoclax (ABT-263) with or without 1 µM selumetinib (Sel) for 48 hours. **k**, **l** A375 (**k**) and SK-MEL-30 (**l**) cells were treated with the indicated concentrations of venetoclax with or without 1 µM selumetinib (Sel) for 48 hours. **e**-**l** Apoptosis was assessed by Annexin V positivity using flow cytometry. Results (**a**-**l**) are mean ± SD of three or more independent experiments.

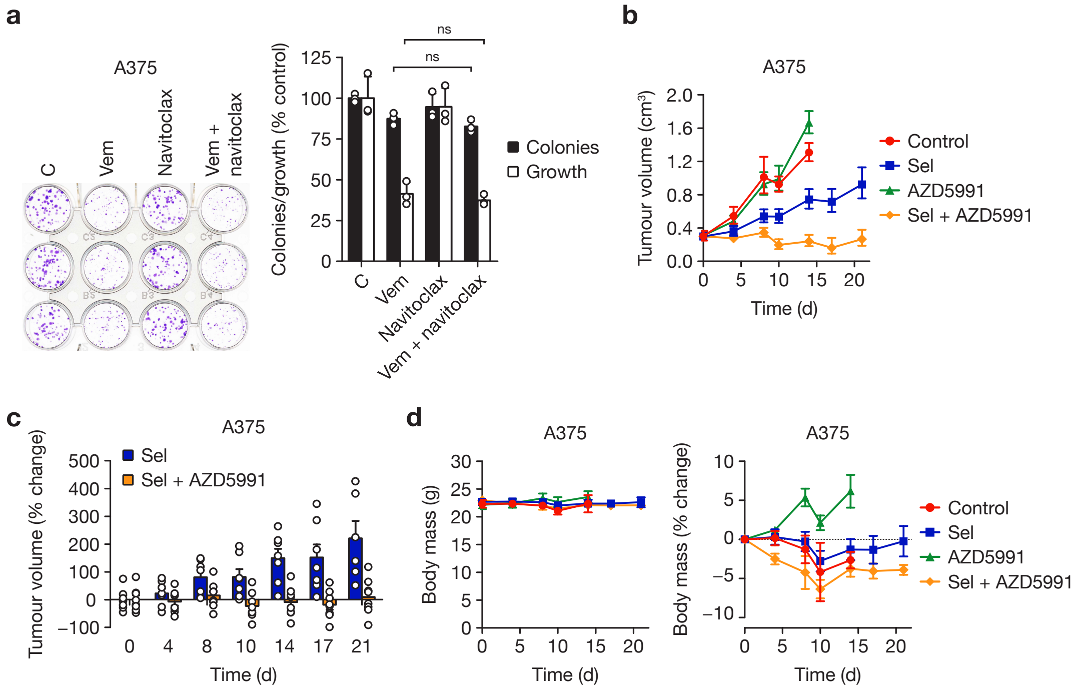

Supplementary Fig. 8 ERK1/2 pathway inhibitors combine with the MCL1 antagonist AZD5991 to inhibit clonogenic survival and tumour growth in xenografts. **a** A375 cells seeded at low density were treated with DMSO control (C), 2 μM vemurafenib (Vem), 1 μM navitoclax (ABT-263) or the combination as indicated for 72 hours, after which time cells were washed and grown for a further four days. Colonies were visualised by crystal violet staining and counted, and cell growth assessed following solubilisation. Results are mean ± SD of three independent experiments. **b** Results as described in Fig. 3d but with tumour growth shown as geometric mean ± SEM. **c** Results as described in Fig. 3e showing the individual data points overlaid. Error bars are mean ± SEM. **d** As in Fig. 3d, female nude athymic mice were implanted subcutaneously with A375 cells and randomised 21 days later for dosing with either vehicle only (Control, n = 5), 25 mg kg^{-1} selumetinib (Sel, n = 7) twice daily with an 8 hour interval, 60 mg kg^{-1} AZD5991 (n = 7) intravenously once weekly, or the combination of 25 mg kg^{-1} selumetinib and 60 mg kg^{-1} AZD5991 (Sel + AZD5991, n = 8) for a further 21 days. Mouse body mass was recorded twice weekly and results are mean ± SEM.

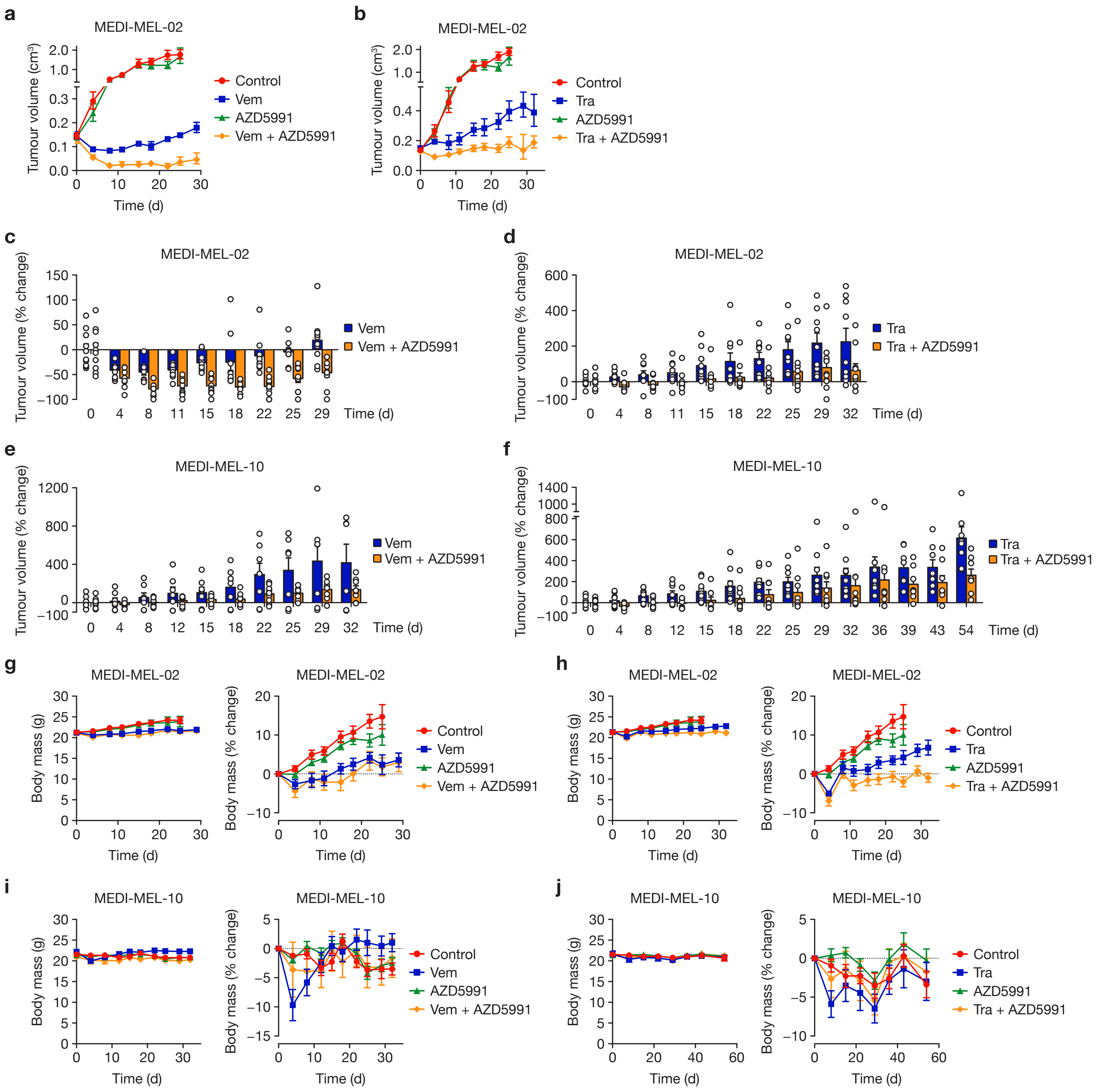

Supplementary Fig. 9 Combinations of ERK1/2 pathway inhibitors and the MCL1 antagonist AZD5991 regress or inhibit tumour growth and are tolerated in vivo. **a**, **b** Results as described in Fig. 4g, i but with tumour growth shown as geometric mean ± SEM. **c**-**f** Results as described in Fig. 4h, j, l, n showing the individual data points overlaid. Error bars are mean ± SEM. **g**-**j** As in Fig. 4g-n, NOD SCID mice implanted subcutaneously with MEDI-MEL-02 (**g**, **h**) or MEDI-MEL-10 (**i**, **j**) tumour pieces and dosed (n = 9 or 10 per group) as indicated with vehicle only (Control), 20 mg kg^{-1} vemurafenib (Vem) twice daily with an 8 hour interval, 1 mg kg^{-1} trametinib (Tra) daily, and/or 60 mg kg^{-1} AZD5991 three times per week. Mice were dosed on these schedules throughout the duration of the experiment, except in (**j**) for which dosing ceased after day 32. Mouse body mass was recorded twice weekly and results are mean ± SEM.

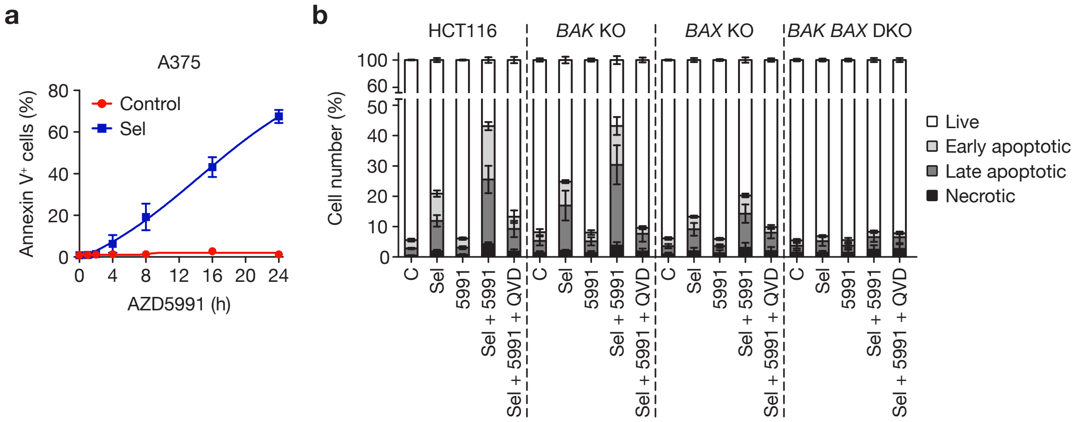

Supplementary Fig. 10 ERK1/2 pathway inhibitors prime tumour cells for rapid induction of BAK/BAX-dependent apoptosis upon MCL1 antagonism. **a** A375 cells were treated with DMSO vehicle only (Control) or 1 µM selumetinib (Sel) for 24 hours as indicated. 1 µM AZD5991 was then added for the indicated times and apoptosis assessed by Annexin V staining and flow cytometry. **b** HCT116, HCT116 *BAK* KO, HCT116 *BAX* KO and HCT116 *BAK BAX* DKO isogenic cell lines were treated with DMSO control (C), 2 µM selumetinib (Sel), 1 µM AZD5991 or 2 µM selumetinib plus 1 µM AZD5991 (Sel + AZD5991) with or without 20 µM Q-VD-OPh (Sel + AZD5991 + QVD) for 48 hours. Annexin V or DAPI positive cells were assessed by flow cytometry. **a**, **b** Results are mean ± SD of four (**a**) or six (**b**) independent experiments.

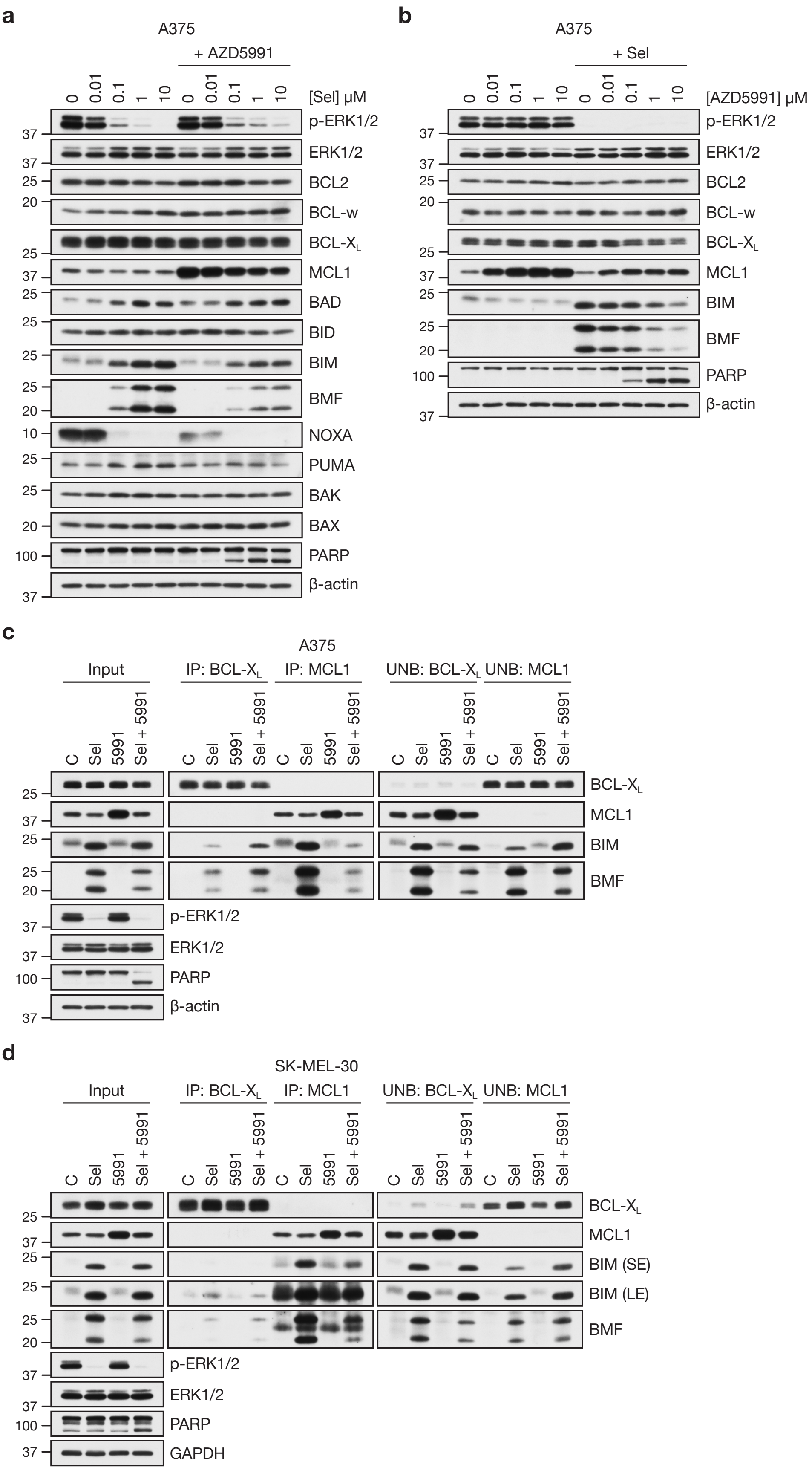

Supplementary Fig. 11 Apoptosis induced by combined ERK1/2 pathway and MCL1 inhibition causes BIM and BMF redistribution to other pro-survival proteins. **a** A375 cells were treated with the indicated concentrations of selumetinib (Sel) with or without 1 μM AZD5991 for 24 hours. Whole-cell lysates were subjected to SDS-PAGE and western blotted with the indicated antibodies. **b** A375 cells were treated with the indicated concentrations of AZD5991 with or without 1 μM selumetinib (Sel) for 24 hours. Whole-cell lysates were subjected to SDS-PAGE and western blotted with the indicated antibodies. **c** A375 cells were treated with DMSO vehicle only (C and 5991) or 1 μM selumetinib (Sel and Sel + 5991) for 24 hours followed by DMSO only (C and Sel) or 1 μM AZD5991 (5991 and Sel + 5991) for a further four hours. Lysates (input) were subjected to immunoprecipitation with antibodies to BCL-X_L or MCL1. Input lysates, immunoprecipitates (IP) and supernatant unbound fractions (UNB) were then western blotted with the indicated antibodies. **d** SK-MEL-30 cells were treated with DMSO vehicle only (C and 5991) or 1 μM selumetinib (Sel and Sel + 5991) for 24 hours followed by DMSO only (C and Sel) or 1 μM AZD5991 (5991 and Sel + 5991) for a further four hours. Lysates (input) were subjected to immunoprecipitation with antibodies to BCL-X_L or MCL1. Input lysates, immunoprecipitates (IP) and supernatant unbound fractions (UNB) were then western blotted with the indicated antibodies.

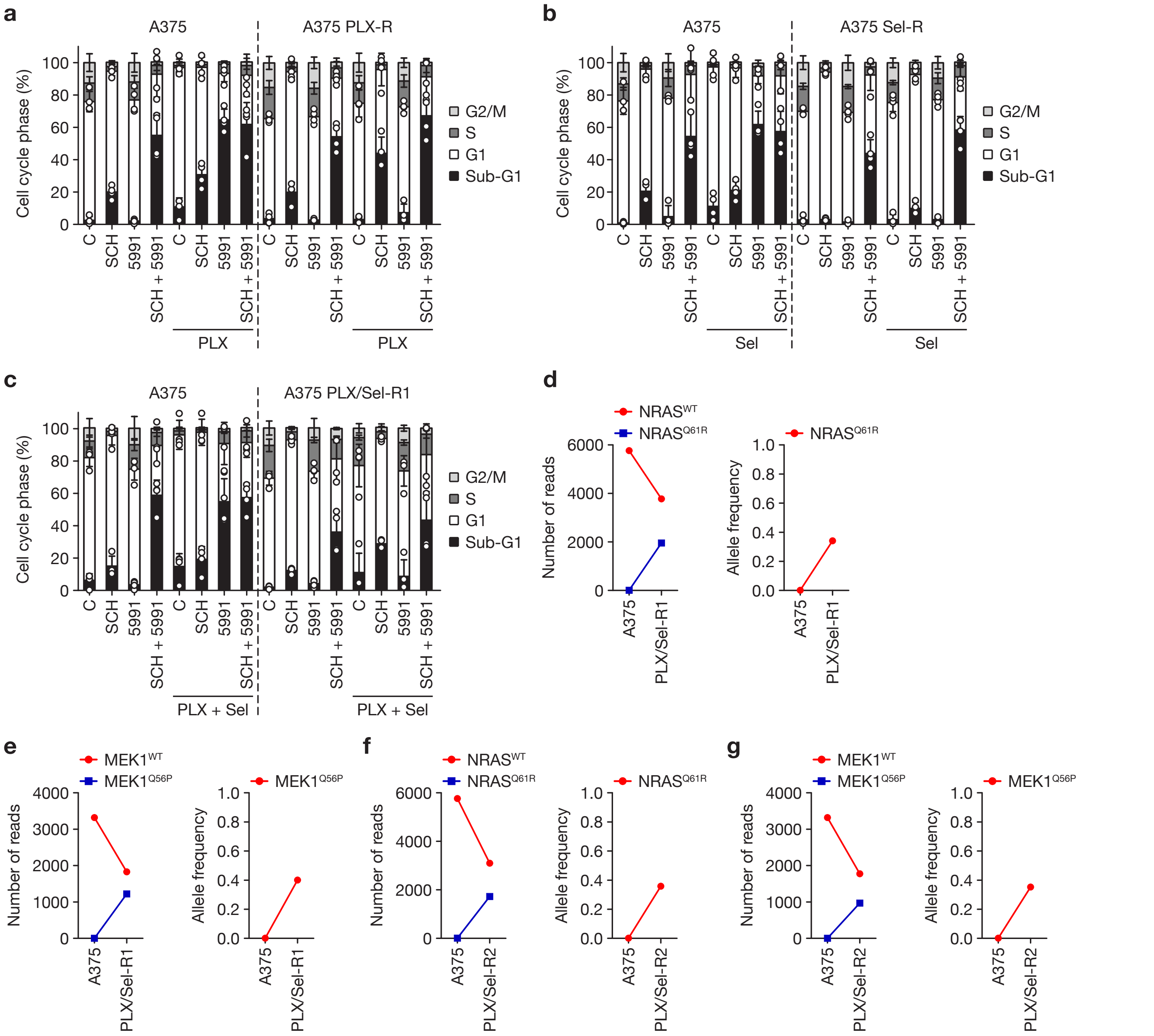

Supplementary Fig. 12 Combined ERK1/2 and MCL1 inhibition can overcome acquired resistance to BRAFi and/or MEKi. **a** A375 and PLX4720-resistant A375 (A375 PLX-R) cells were washed and treated with DMSO control only (C), 0.3 μM SCH772984 (SCH), 1 μM AZD5991 (5991) or the combination (SCH + 5991) for 48 hours, either in the presence (PLX) or absence of 0.66 μM PLX4720, the normal growth medium of A375 PLX-R. **b** A375 and selumetinib-resistant A375 (A375 Sel-R) cells were washed and treated with DMSO control only (C), 0.3 μM SCH772984 (SCH), 1 μM AZD5991 (5991) or the combination (SCH + 5991) for 48 hours, either in the presence (Sel) or absence of 0.5 μM selumetinib, the normal growth medium of A375 Sel-R. **c** A375 and selumetinib/PLX4720-resistant A375 (A375 PLX/Sel-R1) cells were washed and treated with 0.3 μM SCH772984 (SCH), 1 μM AZD5991 (5991) or the combination (SCH + 5991) for 48 hours, either in the presence (PLX + Sel) or absence of 0.33 μM PLX4720 and 0.25 μM selumetinib, the normal growth medium of A375 PLX/Sel-R1. **a**-**c** Cell cycle profile was determined by propidium iodide staining and flow cytometry. Results show mean ± SD of four independent experiments. **d**-**g** DNA extracted from A375, A375 PLX/Sel-R1 (**d**, **e**) and A375 PLX/Sel-R2 (**f**, **g**) cells was subjected to next-generation sequencing. Number of reads and allele frequencies for detected NRAS^{Q61R} (**d**, **f**) and MEK1^{Q56P} (**e**, **g**) mutations are shown.

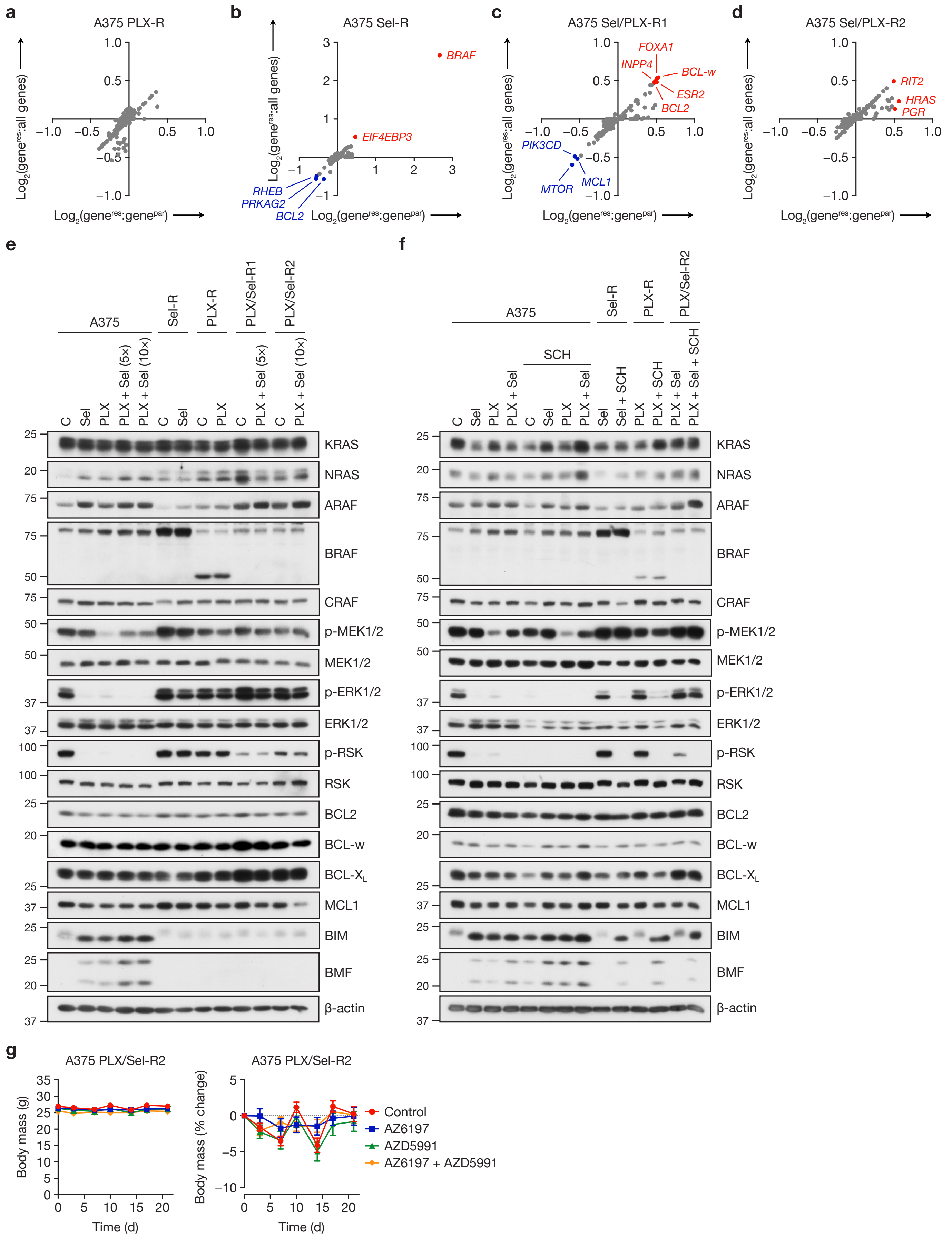

Supplementary Fig. 13 A375 cells acquire resistance to PLX4720 and/or selumetinib by reinstating ERK1/2 signalling. **a-d** DNA extracted from A375 (**a-d**) and PLX4720-resistant A375 (A375 PLX-R) cells (**a**), selumetinib-resistant A375 (A375 Sel-R) cells (**b**) and selumetinib/PLX4720-resistant A375 (A375 PLX/Sel-R1 and A375 PLX/Sel-R2) cells (**c**, **d**) was subjected to next-generation sequencing and copy number analysis. **e** A375, A375 Sel-R, A375 PLX-R, A375 PLX/Sel-R1 (5×IC_{50}) and A375 PLX/Sel-R2 (10×IC_{50}) cells were washed and treated with DMSO only control (C), 0.5 μM selumetinib (Sel), 0.66 μM PLX4720 (PLX), 0.25 μM selumetinib plus 0.33 μM PLX4720 (PLX + Sel (5×)) or 0.5 μM selumetinib plus 0.66 μM PLX4720 (PLX + Sel (10×)) as indicated. Whole-cell lysates were western blotted with the indicated antibodies. **f** A375, A375 Sel-R cells, A375 PLX-R cells and A375 PLX/Sel-R2 cells were washed and treated with DMSO only control (C), 0.5 μM selumetinib (Sel), 0.66 μM PLX4720 (PLX) or 0.5 μM selumetinib plus 0.66 μM PLX4720 (PLX + Sel), as indicated, either with or without 0.3 μM SCH772984. Whole-cell lysates were western blotted with the indicated antibodies. **g** As in Figure 8b female athymic nude mice were injected subcutaneously with A375 PLX/Sel-R2 cells and dosed with vehicle only (Control, n = 10), 50 mg kg^{-1} AZ13776197 (AZ6197, n = 10) orally once daily, 60 mg kg^{-1} AZD5991 (AZD5991, n = 10) once weekly intravenously, or the combination (AZ6197 + AZD5991, n = 10). Mouse body mass was recorded twice weekly and results are mean ± SEM.

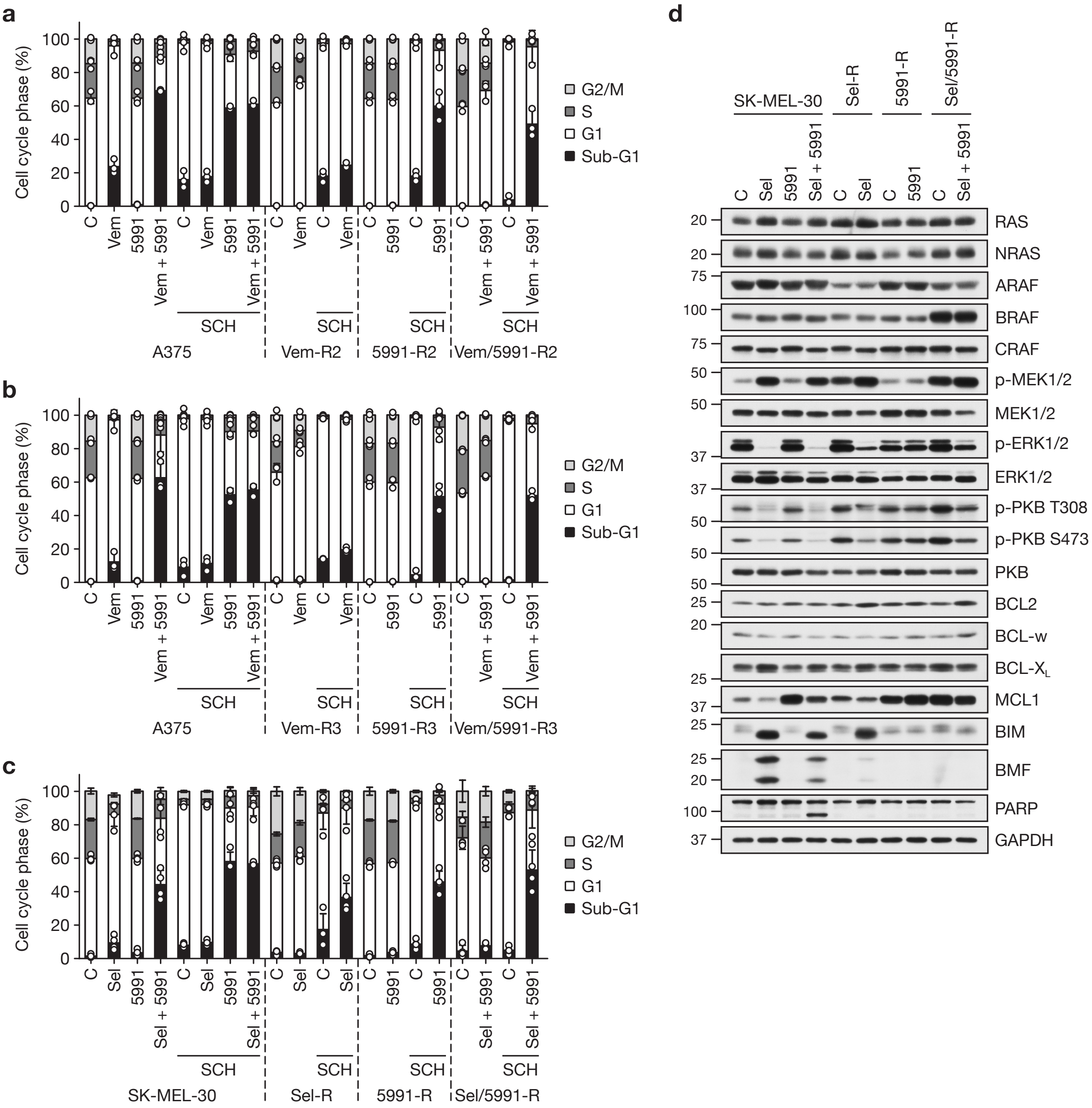

Supplementary Fig. 14 Melanoma cells acquire resistance to combined ERK1/2 pathway and MCL1 inhibition by reinstating ERK1/2 signalling. **a**, **b** Two independently derived sets (R2 and R3) of A375, vemurafenib-resistant A375 (Vem-R) cells, AZD5991-resistant A375 (5991-R) cells and vemurafenib/AZD5991-resistant A375 (Vem/5991-R) cells were washed and treated with DMSO only control (C), 2 µM vemurafenib (Vem) and/or 1 µM AZD5991 (5991) with or without 1 µM SCH772984 (SCH) as indicated for 48 hours. **c** SK-MEL-30, selumetinib-resistant SK-MEL-30 (Sel-R) cells, AZD5991-resistant SK-MEL-30 (5991-R) cells and selumetinib/AZD5991-resistant SK-MEL-30 (Sel/5991-R) cells were washed and treated with DMSO only control (C), 1 µM selumetinib (Sel) and/or 1 µM AZD5991 (5991) with or without 1 µM SCH772984 (SCH) as indicated for 48 hours. **a**-**c** Cell cycle profile was determined by propidium iodide staining and flow cytometry. Results are mean ± SD of three or four independent experiments. **d** SK-MEL-30, selumetinib-resistant SK-MEL-30 (Sel-R) cells, AZD5991-resistant SK-MEL-30 (5991-R) cells and selumetinib/AZD5991-resistant SK-MEL-30 (Sel/5991-R) cells were washed and treated with DMSO only control (C), 1 µM selumetinib (Sel) and/or 1 µM AZD5991 (5991) as indicated for 24 hours. Whole-cell lysates were subjected to SDS-PAGE and western blotted with the indicated antibodies.

Supplementary Table 1. ERK1/2 pathway component mutations in the cell lines used for high-throughput combination assays.

		Gene						
	**Cell line**	***BRAF***	***EGFR***	***KRAS***	***HRAS***	***MAP2K1***	***NF1***	***NRAS***
**Colorectal**	**CO115**	V600E	N/A	WT	N/A	N/A	N/A	N/A
	**COLO205**	V600E	WT	WT	WT	WT	WT	WT
	**DLD1**	WT	WT	G13D	WT	WT	A2617V	WT
	**HCA7**	WT	WT	WT	WT	WT	P228L	WT
	**HCT116**	WT	WT	G13D	WT	WT	P388T; P678fs*10; I679fs*21	WT
	**HT29**	T119S; V600E	WT	WT	WT	WT	WT	WT
	**LoVo**	WT	WT	G13D	WT	WT	R1695Q	WT
	**NCI-H747**	WT	WT	G13D	WT	WT	WT	WT
	**SW48**	WT	G719S	WT	WT	Q56P; H119Y; D351G	WT	WT
	**SW480**	WT	WT	G12V	WT	WT	WT	WT
	**SW620**	WT	WT	G12V	WT	WT	WT	WT
	**SW837**	WT	WT	G12C	WT	WT	WT	WT
**Melanoma**	**A2058**	V600E	WT	WT	WT	P124S	WT	WT
	**A375**	V600E	WT	WT	WT	WT	WT	WT
	**CHL-1**	WT	V1011M	WT	WT	WT	WT	WT
	**HMCB**	WT	WT	WT	WT	WT	Q535H	WT[‡]
	**MEL-JUSO**	WT	WT	WT	G13D	WT	L1779P	Q61L
	**MeWo**	WT	WT	WT	WT	WT	Q1336*	WT
	**SK-MEL-2**	WT	WT	WT	WT	WT	N1250fs*3	Q61R
	**SK-MEL-5**	V600E	WT	WT	WT	WT	WT	WT
	**SK-MEL-28**	V600E	P753S	WT	WT	WT	WT	WT
	**SK-MEL-30**	E275K; D287H	WT	WT	WT	WT	WT	Q61K
	**WM266-4**	V600E[†]	WT	WT	WT	WT	WT	WT
	**WM852**	WT	N/A	N/A	N/A	N/A	N/A	Q61R

* Introduces stop codon.
[†] Variously reported as V600D or V600E.
[‡] HMCB reported to be $NRAS^{Q61K}$ mutant in Davies et al.[1].
Data curated from Catalogue of Somatic Mutations in Cancer (COSMIC; https://cancer.sanger.ac.uk/cosmic), except SW480, HMCB, MeWo and WM266-4 which show data from the Cancer Cell Line Encyclopedia (CCLE; https://portals.broadinstitute.org/ccle); fs, frameshift; N/A, not available; WT, wild type. Predicted zygosity: heterozygous (blue); homozygous (red); unknown (black).

Supplementary Table 2. Loewe synergy scores by cell line.

	Cell line	Mutations	Loewe synergy score			
			Tra + 5991	Tra + 4320	Sel + 5991	Sel + 4320
**Colorectal**	**CO115**	BRAF	2.35	0.77	1.50	0.11
	**COLO205**	BRAF	31.85	57.41	25.17	33.46
	**DLD1**	KRAS, NF1	2.58	16.30	1.51	5.54
	**HCA7**	NF1	37.10	26.58	21.96	20.11
	**HCT116**	KRAS, NF1	7.31	12.21	1.46	4.66
	**HT29**	BRAF	7.20	25.24	6.98	14.85
	**LoVo**	KRAS, NF1	4.51	5.36	2.46	2.42
	**NCI-H747**	KRAS	1.72	14.58	0.45	9.35
	**SW48**	EGFR, MEK1	16.19	19.96	11.97	9.18
	**SW480**	KRAS	9.16	19.56	8.32	9.76
	**SW620**	KRAS	9.56	12.34	5.78	9.74
	**SW837**	KRAS	7.30	17.67	2.52	13.37
**Melanoma**	**A2058**	BRAF, MEK1	30.56	16.45	10.27	5.78
	**A375**	BRAF	28.63	22.12	25.42	12.34
	**CHL-1**	EGFR	16.10	10.48	16.55	7.14
	**HMCB**	NF1	7.42	11.26	3.68	5.70
	**MEL-JUSO**	HRAS, NRAS, NF1	22.28	29.23	15.28	15.48
	**MeWo**	NF1	14.18	18.56	11.06	5.21
	**SK-MEL-2**	NRAS, NF1	21.24	20.16	16.46	5.08
	**SK-MEL-5**	BRAF	26.53	18.31	19.72	12.17
	**SK-MEL-28**	BRAF, EGFR	33.86	37.10	20.24	29.79
	**SK-MEL-30**	NRAS, BRAF	27.65	17.32	22.78	9.97
	**WM266-4**	BRAF	37.67	37.97	34.36	37.08
	**WM852**	NRAS	20.21	20.12	9.39	7.91

Supplementary Table 3. Reagents and resources used in this study.

REAGENT or RESOURCE		SOURCE	IDENTIFIER
**Antibodies**			
α-tubulin	WB 1:500	Sigma-Aldrich	Cat#T9026
Anti-mouse Alexa Fluor 488 antibody	FC 1:200	Thermo Fisher Scientific	Cat#A-11001
Anti-mouse DyLight 800 4X PEG conjugate	WB 1:15000	Cell Signaling Technology	Cat#5257
Anti-rabbit DyLight 800 4X PEG conjugate	WB 1:15000	Cell Signaling Technology	Cat#5151
ARAF; rabbit polyclonal	WB 1:1000	Cell Signaling Technology	Cat#4432
β-actin; mouse monoclonal (clone AC-15)	WB 1:2000	Sigma-Aldrich	Cat#A5441
BAD; rabbit monoclonal (D24A9)	WB 1:1000	Cell Signaling Technology	Cat#9239
BAK; rabbit polyclonal	WB 1:500	Santa Cruz Biotechnology	Cat#sc-832
BAX; rabbit polyclonal	WB 1:500	Santa Cruz Biotechnology	Cat#sc-493
BAX; mouse monoclonal (clone 6A7)	FC 1:50<br>WB 1:500	BD Biosciences	Cat#556467
BCL2; mouse monoclonal (clone C-2)	WB 1:500	Santa Cruz Biotechnology	Cat#sc-7382
BCL-w; rabbit monoclonal (clone 31H4)	WB 1:1000	Cell Signaling Technology	Cat#2724
BCL-X_L; rabbit monoclonal (clone 54H6)	WB 1:1000	Cell Signaling Technology	Cat#2764
BCL-X_L; rabbit polyclonal	WB 1:1000	Cell Signaling Technology	Cat#2762
BID; rabbit polyclonal	WB 1:1000	Cell Signaling Technology	Cat#2002
BIM; rabbit monoclonal (clone C34C5)	WB 1:1000	Cell Signaling Technology	Cat#2933
BIM; rabbit polyclonal	WB 1:1000	Merck Millipore	Cat#AB17003
BMF; rat monoclonal (clone 9G10)	WB 1:500	Enzo Life Sciences	Cat#ALX-804-343
BRAF; rabbit polyclonal	WB 1:500	Santa Cruz Biotechnology	Cat#sc-166
CRAF; rabbit polyclonal	WB 1:500	Santa Cruz Biotechnology	Cat#sc-227
ERK1/2; rabbit monoclonal (clone 137F5)	WB 1:1000	Cell Signaling Technology	Cat#4695
GAPDH; rabbit monoclonal (clone EPR16891)	WB 1:2000	Abcam	Cat#ab181602
Goat anti-mouse IgG (H + L)-HRP conjugate	WB 1:3000	Bio-Rad	Cat#1706516
Goat anti-rabbit IgG (H + L)-HRP conjugate	WB 1:3000	Bio-Rad	Cat#1706515
KRAS; rabbit polyclonal	WB 1:500	Proteintech	Cat#12063-1-AP
MCL1; rabbit monoclonal (clone D2W9E)	WB 1:1000	Cell Signaling Technology	Cat#94296
MCL1; rabbit polyclonal	WB 1:500	Santa Cruz Biotechnology	Cat#sc-819
MEK1/2; rabbit polyclonal	WB 1:1000	Cell Signaling Technology	Cat#9122
NOXA; mouse monoclonal (clone 114C307)	WB 1:200	Merck Millipore	Cat#OP180
NRAS; rabbit polyclonal	WB 1:500	Santa Cruz Biotechnology	Cat#sc-519
PARP; rabbit polyclonal	WB 1:1000	Cell Signaling Technology	Cat#9542
RSK; rabbit monoclonal (clone 32D7)	WB 1:1000	Cell Signaling Technology	Cat#9355
Phospho-ERK1/2 T185 Y187/T202 Y204; rabbit polyclonal	WB 1:1000	Cell Signaling Technology	Cat#9101
Phospho-MEK1/2 S218 S222/S222 S226; rabbit polyclonal	WB 1:1000	Cell Signaling Technology	Cat#9121
Phospho-RSK TS359; rabbit monoclonal (D1E9)	WB 1:1000	Cell Signaling Technology	Cat#8753
PUMA; rabbit monoclonal (D30C10)	WB 1:1000	Cell Signaling Technology	Cat#12450
**Biological Samples**			
Colorectal and melanoma patient-derived xenograft tissue		AstraZeneca PDX library	N/A
**Chemicals, peptides, and recombinant proteins**			
Recombinant human BCL-X_L		R&D Systems	Cat#894-BX-050
Recombinant human MCL1		Cloud-Clone Corp	Cat#RPC615Hu01
A-1155463 BCL-X_L inhibitor		Adooq Bioscience	Cat#A16112<br>CAS: 1235034-55-5
AZ6197 ERK1/2 inhibitor		Provided by AstraZeneca	N/A
AZD4320 BCL-w/BCL-X_L/BCL2 inhibitor		Provided by AstraZeneca	N/A
AZD5991 MCL1 inhibitor		Provided by AstraZeneca	N/A
PLX4720 RAF inhibitor		Selleck Chemicals	Cat#S1152;<br>CAS: 918505-84-7
Q-VD-OPh pan-caspase inhibitor		Adooq Bioscience	Cat#A14915; CAS: 1135695-98-5
SCH772984 ERK1/2 inhibitor		Selleck Chemicals	Cat#S7101; CAS: 942183-80-4
Selumetinib (AZD6244) MEK1/2 inhibitor		Provided by AstraZeneca	CAS: 606143-52-6
Trametinib MEK1/2 inhibitor		Selleck Chemicals	Cat#S2673; CAS: 871700-17-3
Vemurafenib RAF inhibitor		Selleck Chemicals	Cat#S1267; CAS: 918504-65-1
Venetoclax BCL2 inhibitor		MedChemExpress	CAS: 1257044-40-8<br>Cat#HY-15531
**Critical commercial assays**			
Caspase-Glo 3/7 Assay		Promega	Cat#G8091
AllPrep DNA/RNA/miRNA Universal Kit		Qiagen	Cat#80224

HaloPlex Target Enrichment System for Illumina Sequencing Version D.3	Agilent Technologies	N/A
Illumina HiSeq 2500 System	Illumina	N/A
**Deposited data**		
DNA sequencing	NCBI Sequence Read Archive	PRJNA531927
**Experimental models: cell lines**		
Human: A2058	ATCC	Cat#CRL-11147; RRID: CVCL_1059
Human: A375	Laboratory of Richard Marais	RRID: CVCL_5649
Human: A549	ATCC	Cat#CCL-185; RRID: CVCL_0023
Human: AsPC-1	ATCC	Cat#CRL-1682; RRID: CVCL_0152
Human: BxPC3	ATCC	Cat#CRL-1687 RRID: CVCL_0186
Human: Caco-2	ATCC	Cat#HTB-37; RRID: CVCL_0025
Human: Calu-6	ATCC	Cat#HTB-56; RRID: CVCL_0236
Human: Capan-1	ATCC	Cat#HTB-79; RRID: CVCL_0237
Human: CFPAC-1	ATCC	Cat#CRL-1918; RRID: CVCL_1119
Human: CHL-1	ATCC	Cat#CRL-9446; RRID: CVCL_1122
Human: CO115	Laboratory of Richard Hamelin	RRID: CVCL_D102
Human: COLO205	ATCC	Cat#CCL-222; RRID: CVCL_0218
Human: DLD1	ATCC	Cat#CCL-221; RRID: CVCL_0248
Human: HARA	JCRB	Cat#JCRB1080.0 RRID: CVCL_2914
Human: HCA7	ECACC	Cat#06061902; RRID: CVCL_0289
Human: HCC15	DSMZ	Cat#ACC-496; RRID: CVCL_2057
Human: HCT116	Laboratory of Richard Youle	RRID: CVCL_0291
Human: HCT116 *BAK* KO	Laboratory of Richard Youle	N/A
Human: HCT116 *BAX* KO	Laboratory of Richard Youle	N/A
Human: HCT116 *BAK BAX* DKO	Laboratory of Richard Youle	N/A
Human: HCT8	ECACC	Cat#90032006; RRID: CVCL_2478
Human: HMCB	ATCC	Cat#CRL-9607; RRID: CVCL_3317
Human: HPAC	ATCC	Cat#CRL-2119; RRID: CVCL_3517
Human: HPAF-II	ATCC	Cat#CRL-1997; RRID: CVCL_0313
Human: Hs 700T	ATCC	Cat#HTB-147 RRID: CVCL_0858
Human: HT29	ATCC	Cat#HTB-38; RRID: CVCL_0320
Human: HuP-T4	ECACC	Cat#93121056 RRID: CVCL_1300
Human: LoVo	Laboratory of Kevin Ryan	RRID: CVCL_0399
Human: LS174T	ATCC	Cat#CL-188; RRID: CVCL_1384
Human: LS411N	ATCC	Cat#CRL-2159; RRID: CVCL_1385
Human: MEL-JUSO	Laboratory of Judith Johnson	RRID: CVCL_1403
Human: MeWo	ATCC	Cat#HTB-65; RRID: CVCL_0445
Human: MIA PaCa-2	ATCC	Cat#CRM-CRL-1420; RRID: CVCL_0428
Human: NCI-H520	ATCC	Cat#HTB-182; RRID: CVCL_1566

Human: NCI-H727	ATCC	Cat#CRL-5815 RRID: CVCL_1584
Human: NCI-H747	ATCC	Cat#CCL-252; RRID: CVCL_1587
Human: NCI-H838	ATCC	Cat#CRL-5844; RRID: CVCL_1594
Human: NCI-H1299	ATCC	Cat#CRL-5803; RRID: CVCL_0060
Human: NCI-H1473	ATCC	Cat#CRL-5872; RRID:CVCL_1472
Human: NCI-H1793	ATCC	Cat#CRL-5896; RRID: CVCL_1496
Human: NCI-H1869	ATCC	Cat#CRL-5900; RRID: CVCL_1500
Human: NCI-H2122	ATCC	Cat#CRL-5985; RRID: CVCL_1531
Human: NCI-H2286	ATCC	Cat#CRL-5938; RRID: CVCL_1545
Human: Panc 02.03	ATCC	Cat#CRL-2553; RRID: CVCL_1633
Human: Panc 03.27	ATCC	Cat#CRL-2549; RRID: CVCL_1635
Human: Panc 04.03	ATCC	Cat#CRL-2555; RRID: CVCL_1636
Human: Panc 08.13	ATCC	Cat#CRL-2551; RRID: CVCL_1638
Human: Panc 10.05	ATCC	Cat#CRL-2547; RRID: CVCL_1639
Human: PANC-1	ATCC	Cat#CRL-1469; RRID: CVCL_0480
Human: PMWK	ATCC	Cat#CRL-2624; RRID: CVCL_A665
Human: PSN1	ECACC	Cat#94060601; RRID: CVCL_1644
Human: RPMI-7951	ATCC	Cat#HTB-66; RRID: CVCL_1666
Human: SK-CO-1	ATCC	Cat#HTB-39; RRID: CVCL_0626
Human: SK-MEL-2	ATCC	Cat#HTB-68D; RRID: CVCL_0069
Human: SK-MEL-5	ATCC	Cat#HTB-70; RRID: CVCL_0527
Human: SK-MEL-28	ATCC	Cat# HTB-72; RRID: CVCL_0526
Human: SK-MEL-30	DSMZ	Cat#ACC-151; RRID: CVCL_0039
Human: SK-MEL-31	ATCC	Cat#HTB-73; RRID: CVCL_0600
Human: SW1116	ATCC	Cat#CCL-233; RRID: CVCL_0544
Human: SW1417	ATCC	Cat#CCL-238; RRID: CVCL_1717
Human: SW48	ATCC	Cat#CCL-231; RRID: CVCL_1724
Human: SW480	ATCC	Cat#CCL-228; RRID: CVCL_0546
Human: SW620	ATCC	Cat#CCL-227; RRID: CVCL_0547
Human: SW837	ECACC	Cat#91031104; RRID: CVCL_1729
Human: WM266-4	ESTDAB	Cat#ESTDAB-076; RRID: CVCL_2765
Human: WM852	ESTDAB	Cat#ESTDAB-084; RRID: CVCL_6804
Human: YAPC	DSMZ	Cat#ACC-382; RRID: CVCL_1794
Human: PLX4720-resistant A375 (A375 PLX-R) cells	AstraZeneca	N/A
Human: Selumetinib-resistant A375 (A375 Sel-R) cells	AstraZeneca	N/A
Human: 5 × IC_{50} PLX4720/selumetinib-resistant A375 (A375 PLX/Sel-R2) cells	AstraZeneca	N/A
Human: 10 × IC_{50} PLX4720/selumetinib-resistant A375 (A375 PLX/Sel-R1) cells	AstraZeneca	N/A

Human: MEDI-MEL-07	AstraZeneca	N/A
Human: MEDI-MEL-10	AstraZeneca	N/A
**Experimental models: organisms/strains**		
Mouse, female: athymic nude mice (CrTac:NCr-*Foxn1*^{nu})	Taconic Biosciences	Cat#TAC:ncrnu; RRID:IMSR_TAC:ncrnu
Mouse, female: NOD SCID (NOD.CB17-*Prkdc*^{scid}/NCrHsd)	Taconic Biosciences	Cat#17002F
Mouse, female: athymic nude mice (Hsd:Athymic Nude-*Foxn1*^{nu})	Envigo	Cat#069(nu)
**Oligonucleotides**		
BCL2L11 (*BIM*) sequencing primers:<br><br>Fwd: 5′- CTAACCCCGGGAAGTCAGAG-3′;<br>Rev: 5′- TTGACACATCCTCCATTCCC-3′.	Sigma-Aldrich	N/A
BMF sequencing primers:<br><br>5′-CGGCCTAGGTCAGAAAACGTG-3′;<br>5′-GCAGGTGGAAGTCAAGGAATC-3′.	Sigma-Aldrich	N/A
Guide RNAs:<br><br>*BCL2L11* (*BIM*) gRNA #2:<br>5′- caccGCAACCACTATCTCAGTGCAA-3′;<br>5′- aaacTTGCACTGAGATAGTGGTTGC-3′.<br><br>*BMF* gRNA #1<br>5′- caccGAAGAGCTGAAGTCGGCTGA-3′;<br>5′-aaacTCAGCCGACTTCAGCTCTTC-3′.	Sigma-Aldrich	N/A
**Recombinant DNA**		
pSpCas9(BB)-2A-GFP genome editing vector	Feng Zhang, Addgene	Cat#48138
**Software and algorithms**		
FlowJo	FlowJo LLC	https://www.flowjo.com/solutions/flowjo
Genedata Screener 12	Genedata	https://www.genedata.com/products/screener/
GraphPad Prism 6 and 7	GraphPad Software	https://www.graphpad.com/scientific-software/prism/
Ilastik	Ilastik Team	http://ilastik.org/
NIS-Elements Advanced Research	Nikon	https://www.nikoninstruments.com/en_GB/Products/Software/NIS-Elements-Advanced-Research
Study Director	Studylog Systems	https://www.studylog.com/our-products
VarDict pairwise algorithm	AstraZeneca	https://github.com/AstraZeneca-NGS/VarDict
BWA-MEM aligner algorithm	Heng Li, Broad Institute, Cambridge, MA, USA	https://arxiv.org/abs/1303.3997
Seq2C algorithm	AstraZeneca	https://github.com/AstraZeneca-NGS/Seq2C

Supplementary References

1. Davies, H., Bignell, G.R., Cox, C., Stephens, P., Edkins, S. et al. Mutations of the *BRAF* gene in human cancer. *Nature* **417**, 949-954 (2002).
