# Supplementary material for: Targeting melanoma’s MCL1 bias unleashes the apoptotic potential of BRAF and ERK1/2 pathway inhibitors
Source: Nat Commun. 2019 Nov 14;10:5167. doi: 10.1038/s41467-019-12409-w (PMC6856071; doi:10.1038/s41467-019-12409-w)
Supplement: Supplementary file 2 — Description of Additional Supplementary Files [file 41467_2019_12409_MOESM2_ESM.pdf]

Description of additional supplementary files

File name: Supplementary Data 1.

Description: Flow cytometry gating strategy for sorting of GFP-positive cells in CRISPR experiments. (**a**-**e**) SK-MEL-30 cells were transfected with the *BCL2L11* and *BMF* gRNA containing Cas9 plasmids for 48 hours before single GFP-positive live cells were sorted by flow cytometry. Cells were first gated on forward scatter area (FSC-A) versus side scatter area (SSC-A) to give population 1 (P1) that eliminated unwanted events at the extremities (**a**), followed by FSC-A versus forward scatter height (FSC-H) (P2) (**b**) and then SSC-A versus side scatter height (SSC-H) (P3) (**c**) to isolate single cells. DAPI-negative viable cells were then gated (P4) (**d**) and from this population GFP-positive cells sorted (**e**), using the lasers and filter sets indicated. Cellular autofluorescence (695/40 488 nm-A) was monitored in order to exclude apparent GFP-positive cells with high autofluorescence.

File name: Supplementary Data 2.

Description: Uncropped western blot images of Figure 6e. A375 cells and three independent clonally derived WT, BIM and/or BMF null A375 CRISPR clones were treated with 1 µM selumetinib (Sel) as indicated for 24 hours and BIM and BMF expression assessed by western blotting. Images of uncropped membranes are shown.

File name: Supplementary Data 3.

Description: Uncropped western blot images of Figure 6a. A375 cells were treated with the indicated concentrations of vemurafenib (Vem) with or without 1 µM AZD5991 for 24 hours. Whole-cell lysates were then western blotted with the indicated antibodies and images of uncropped membranes are shown.

File name: Supplementary Data 4.

Description: Flow cytometry gating strategy for cell cycle phase analysis using propidium iodide. (**a**-**d**) A375 cells were treated with DMSO only for 48 hours. Cells were then harvested, fixed, stained with propidium iodide (PI) and staining assessed by flow cytometry. Cells were first gated on forward scatter area (FSC-A) versus side scatter area (SSC-A) to give population 1 (P1) that eliminated unwanted events at the extremities (**a**), followed by FSC-A versus side scatter width (SSC-W) (P2) (**b**) and then PI area (PI-A) versus PI height (PI-H) (P3) (**c**) using the lasers and filter sets indicated to isolate single cells only and remove very small debris DNA events. A histogram of PI-A, which corresponds to the DNA content for each event, was then used to quantify the fraction of events in each phase of the cell cycle (**d**).

File name: Supplementary Data 5.

Description: Flow cytometry gating strategy for cell death and apoptosis assessment using Annexin V-DAPI. (**a**-**d**) A375 cells were treated with 1 µM selumetinib for 48 hours in combination with 1 µM AZD5991 for the last 24 hours. Cells were then harvested, stained with Annexin V and DAPI and staining assessed by flow cytometry. Cells were first gated on forward scatter area (FSC-A) versus side scatter area (SSC-A) to give population 1 (P1) that eliminated very small debris events (**a**), followed by SSC-A versus side scatter width (SSC-W) (P2) (**b**) and then FSC-A versus forward scatter width (FSC-W) (P3) (**c**) to isolate single cells only. Annexin V-FITC- and/or DAPI-positive single cells were then quantified using the

lasers and filter sets indicated (**d**). Quadrant gate positions were determined by comparing samples of viable cells to samples exhibiting cell death, which were therefore either negative or positive for Annexin V and/or DAPI staining.

File name: Supplementary Data 6.

Description: Flow cytometry gating strategy for measurement of BAX activation. (**a**-**d**) A375 cells were treated with 1 μM selumetinib for 24 hours and then in combination with 1 μM AZD5991 for a further 1 hour. Cells were then harvested, fixed and incubated with an anti-active BAX antibody followed by an Alexa Fluor 488 labelled secondary antibody. Antibody staining was assessed by flow cytometry. Cells were first gated on forward scatter area (FSC-A) versus side scatter area (SSC-A) to give population 1 (P1) that eliminated very small debris events (**a**), followed by SSC-A versus side scatter width (SSC-W) (P2) (**b**) and then FSC-A versus forward scatter width (FSC-W) (P3) (**c**) to isolate single cells only. A histogram of 530/30 488 nm area (530/30 488 nm-A) signal, which corresponds to levels of active BAX, was then used to quantify the fraction of cells with active BAX (**d**).
