# Supplementary material for: Targeting melanoma’s MCL1 bias unleashes the apoptotic potential of BRAF and ERK1/2 pathway inhibitors
Source: Nat Commun. 2019 Nov 14;10:5167. doi: 10.1038/s41467-019-12409-w (PMC6856071; doi:10.1038/s41467-019-12409-w)
Supplement: Supplementary file 3 — Supplementary Data 1 [file 41467_2019_12409_MOESM3_ESM.pdf]

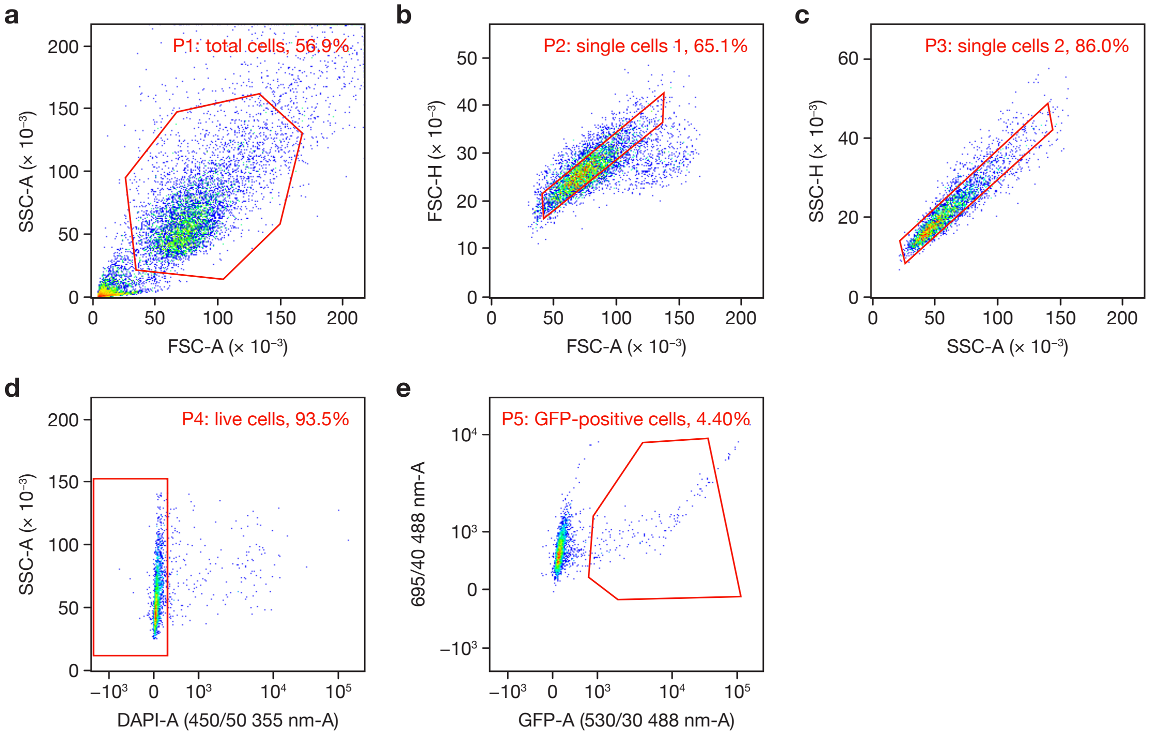

Supplementary Data 1. Flow cytometry gating strategy for sorting of GFP-positive cells in CRISPR experiments. (**a**-**e**) SK-MEL-30 cells were transfected with the *BCL2L11* and *BMF* gRNA containing Cas9 plasmids for 48 hours before single GFP-positive live cells were sorted by flow cytometry. Cells were first gated on forward scatter area (FSC-A) versus side scatter area (SSC-A) to give population 1 (P1) that eliminated unwanted events at the extremities (**a**), followed by FSC-A versus forward scatter height (FSC-H) (P2) (**b**) and then SSC-A versus side scatter height (SSC-H) (P3) (**c**) to isolate single cells. DAPI-negative viable cells were then gated (P4) (**d**) and from this population GFP-positive cells sorted (**e**), using the lasers and filter sets indicated. Cellular autofluorescence (695/40 488 nm-A) was monitored in order to exclude apparent GFP-positive cells with high autofluorescence.
