# Supplementary material for: Targeting melanoma’s MCL1 bias unleashes the apoptotic potential of BRAF and ERK1/2 pathway inhibitors
Source: Nat Commun. 2019 Nov 14;10:5167. doi: 10.1038/s41467-019-12409-w (PMC6856071; doi:10.1038/s41467-019-12409-w)
Supplement: Supplementary file 4 — Supplementary Data 2 [file 41467_2019_12409_MOESM4_ESM.pdf]

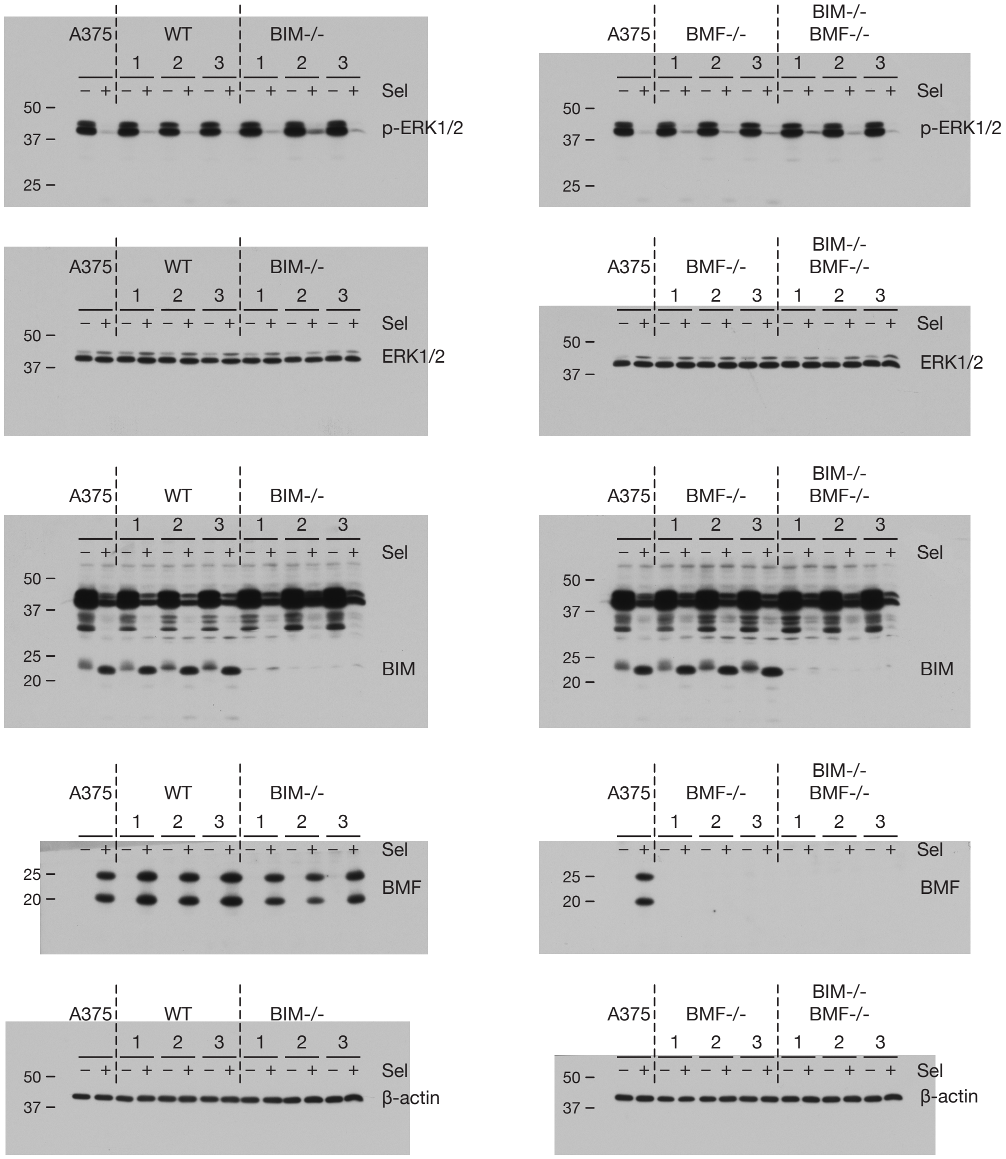

Supplementary Data 2. Uncropped western blot images of Figure 6e. A375 cells and three independent clonally derived WT, BIM and/or BMF null A375 CRISPR clones were treated with 1 μM selumetinib (Sel) as indicated for 24 hours and BIM and BMF expression assessed by western blotting. Images of uncropped membranes are shown.
