# Supplementary material for: Targeting melanoma’s MCL1 bias unleashes the apoptotic potential of BRAF and ERK1/2 pathway inhibitors
Source: Nat Commun. 2019 Nov 14;10:5167. doi: 10.1038/s41467-019-12409-w (PMC6856071; doi:10.1038/s41467-019-12409-w)
Supplement: Supplementary file 5 — Supplementary Data 3 [file 41467_2019_12409_MOESM5_ESM.pdf]

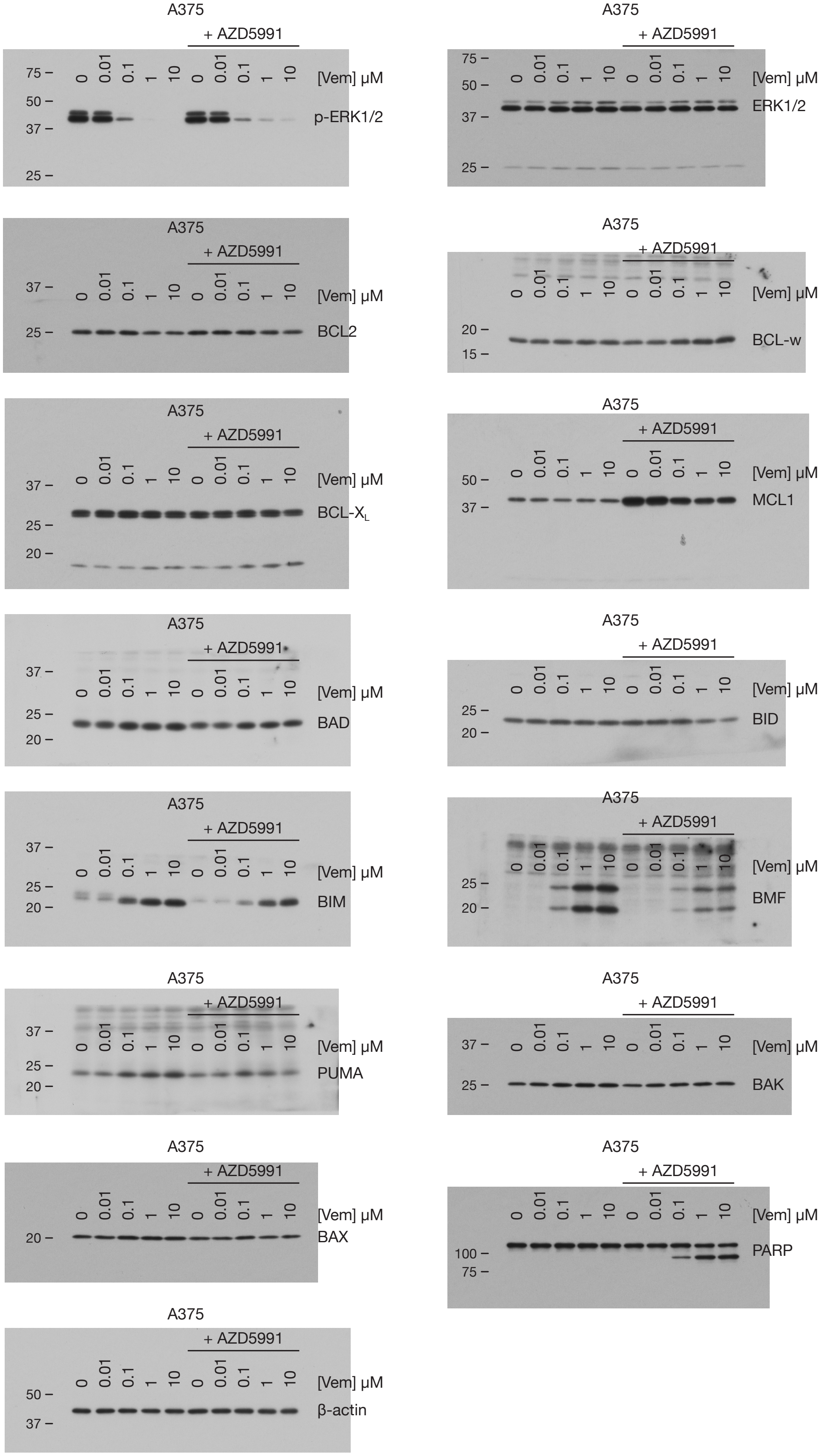

Supplementary Data 3. Uncropped western blot images of Figure 6a. A375 cells were treated with the indicated concentrations of vemurafenib (Vem) with or without 1 μM AZD5991 for 24 hours. Whole-cell lysates were then western blotted with the indicated antibodies and images of uncropped membranes are shown.
