# Supplementary material for: Targeting melanoma’s MCL1 bias unleashes the apoptotic potential of BRAF and ERK1/2 pathway inhibitors
Source: Nat Commun. 2019 Nov 14;10:5167. doi: 10.1038/s41467-019-12409-w (PMC6856071; doi:10.1038/s41467-019-12409-w)
Supplement: Supplementary file 6 — Supplementary Data 4 [file 41467_2019_12409_MOESM6_ESM.pdf]

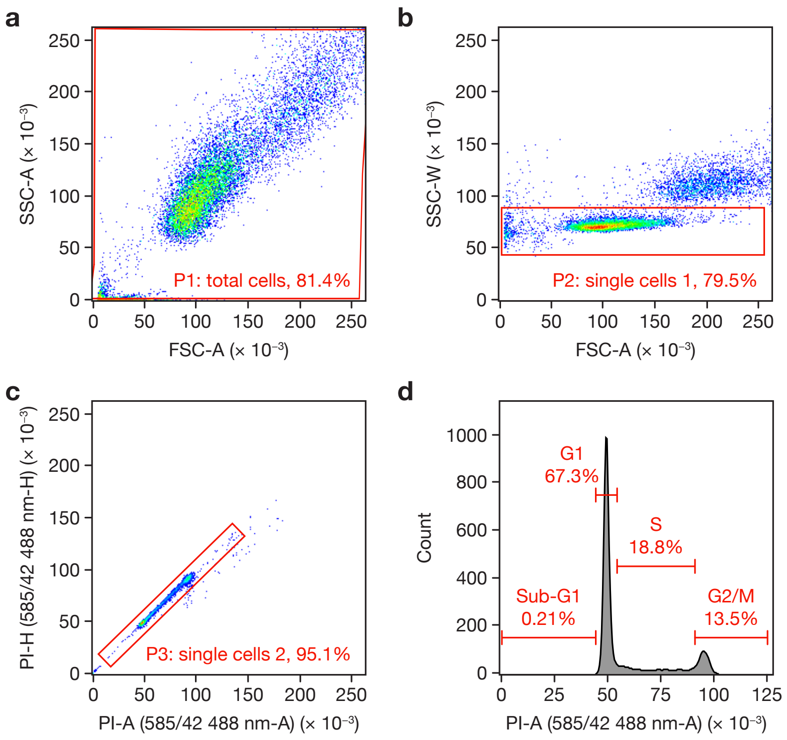


**Supplementary Data 4.** Flow cytometry gating strategy for cell cycle phase analysis using propidium iodide. (**a**-**d**) A375 cells were treated with DMSO only for 48 hours. Cells were then harvested, fixed, stained with propidium iodide (PI) and staining assessed by flow cytometry. Cells were first gated on forward scatter area (FSC-A) versus side scatter area (SSC-A) to give population 1 (P1) that eliminated unwanted events at the extremities (**a**), followed by FSC-A versus side scatter width (SSC-W) (P2) (**b**) and then PI area (PI-A) versus PI height (PI-H) (P3) (**c**) using the lasers and filter sets indicated to isolate single cells only and remove very small debris DNA events. A histogram of PI-A, which corresponds to the DNA content for each event, was then used to quantify the fraction of events in each phase of the cell cycle (**d**).
