# Supplementary material for: Targeting melanoma’s MCL1 bias unleashes the apoptotic potential of BRAF and ERK1/2 pathway inhibitors
Source: Nat Commun. 2019 Nov 14;10:5167. doi: 10.1038/s41467-019-12409-w (PMC6856071; doi:10.1038/s41467-019-12409-w)
Supplement: Supplementary file 7 — Supplementary Data 5 [file 41467_2019_12409_MOESM7_ESM.pdf]

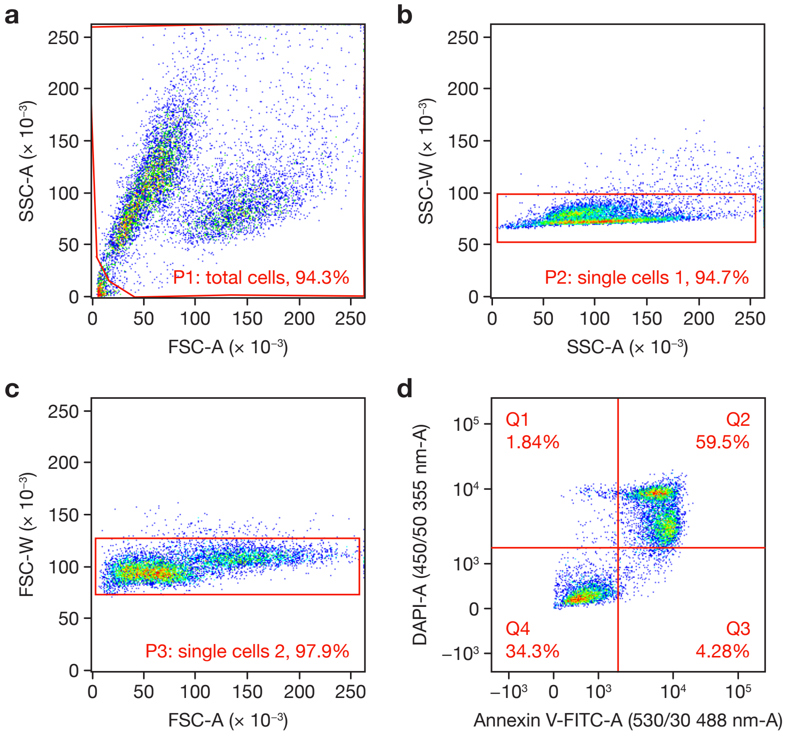

Supplementary Data 5. Flow cytometry gating strategy for cell death and apoptosis assessment using Annexin V-DAPI. (**a**-**d**) A375 cells were treated with 1 µM selumetinib for 48 hours in combination with 1 µM AZD5991 for the last 24 hours. Cells were then harvested, stained with Annexin V and DAPI and staining assessed by flow cytometry. Cells were first gated on forward scatter area (FSC-A) versus side scatter area (SSC-A) to give population 1 (P1) that eliminated very small debris events (**a**), followed by SSC-A versus side scatter width (SSC-W) (P2) (**b**) and then FSC-A versus forward scatter width (FSC-W) (P3) (**c**) to isolate single cells only. Annexin V-FITC- and/or DAPI-positive single cells were then quantified using the lasers and filter sets indicated (**d**). Quadrant gate positions were determined by comparing samples of viable cells to samples exhibiting cell death, which were therefore either negative or positive for Annexin V and/or DAPI staining.
