# Supplementary material for: Targeting melanoma’s MCL1 bias unleashes the apoptotic potential of BRAF and ERK1/2 pathway inhibitors
Source: Nat Commun. 2019 Nov 14;10:5167. doi: 10.1038/s41467-019-12409-w (PMC6856071; doi:10.1038/s41467-019-12409-w)
Supplement: Supplementary file 8 — Supplementary Data 6 [file 41467_2019_12409_MOESM8_ESM.pdf]

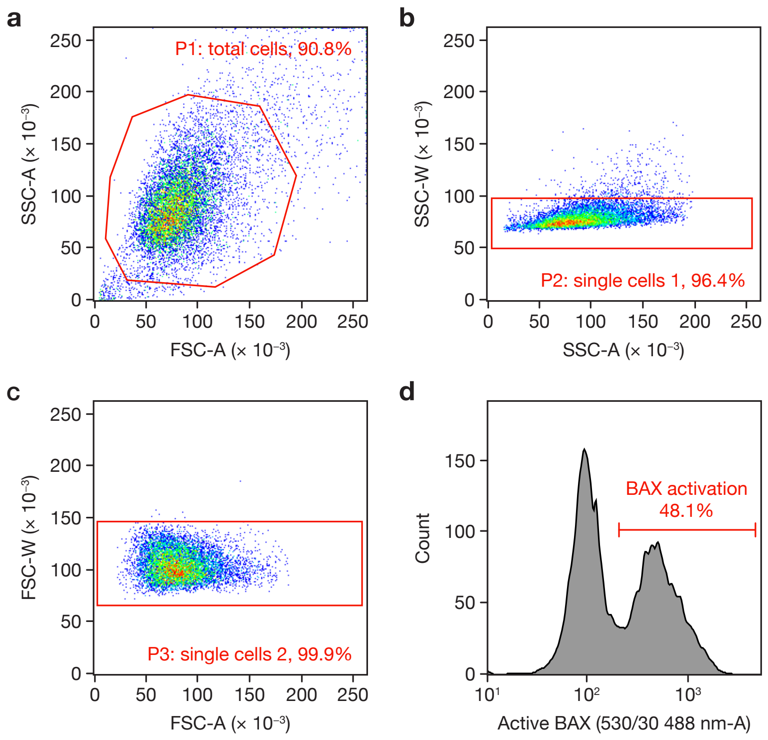

Supplementary Data 6. Flow cytometry gating strategy for measurement of BAX activation. (**a**-**d**) A375 cells were treated with 1 μM selumetinib for 24 hours and then in combination with 1 μM AZD5991 for a further 1 hour. Cells were then harvested, fixed and incubated with an anti-active BAX antibody followed by an Alexa Fluor 488 labelled secondary antibody. Antibody staining was assessed by flow cytometry. Cells were first gated on forward scatter area (FSC-A) versus side scatter area (SSC-A) to give population 1 (P1) that eliminated very small debris events (**a**), followed by SSC-A versus side scatter width (SSC-W) (P2) (**b**) and then FSC-A versus forward scatter width (FSC-W) (P3) (**c**) to isolate single cells only. A histogram of 530/30 488 nm area (530/30 488 nm-A) signal, which corresponds to levels of active BAX, was then used to quantify the fraction of cells with active BAX (**d**).
